# Transcriptional and Functional Analysis Shows Sodium Houttuyfonate-Mediated Inhibition of Autolysis in *Staphylococcus aureus*

**DOI:** 10.3390/molecules16108848

**Published:** 2011-10-21

**Authors:** Guoxing Liu, Hua Xiang, Xudong Tang, Kaiyu Zhang, Xiuping Wu, Xuelin Wang, Na Guo, Haihua Feng, Guangming Wang, Lihui Liu, Qiyun Shi, Fengge Shen, Mingxun Xing, Peng Yuan, Mingyuan Liu, Lu Yu

**Affiliations:** 1 Key Laboratory for Zoonosis Research, Ministry of Education, Institute of Zoonosis, College of Animal Science and Veterinary Medicine, Jilin University, Changchun 130062, China; 2 Department of Food Quality and Safety, College of Quartermaster Technology, Jilin University, Changchun 130062, China; 3 State Key Laboratory for Molecular Virology and Genetic Engineering, Chinese Center for Disease Control and Prevention, Beijing 100176, China; 4 Key Lab for New Drug Research of TCM, Research Institute of Tsinghua University in Shenzhen, Shenzhen 518057, China; 5 Department of Infectious Diseases, First Hospital of Jilin University, Changchun 130021, China

**Keywords:** *Staphylococcus aureus*, sodium houttuyfonate, GeneChip, transcription

## Abstract

Sodium houttuyfonate (SH), an addition compound of sodium bisulfite and houttuynin,**s**howed *in vitro* antibacterial activity against 21 *Staphylococcus aureus *(*S. aureus*) strains grown in planktonic cultures. Microarray results showed decreased levels of autolysin *atl*, *sle1*, *cidA* and *lytN* transcripts in the SH-treated strain as compared to the control strain, consistent with the induction of the autolytic repressors *lrgAB *and *sarA* and with the downregulation of the positive regulators *agrA* and *RNAIII*. Triton X-100-induced autolysis was significantly decreased by SH in *S. aureus* ATCC 25923, and quantitative bacteriolytic assays and zymographic analysis demonstrated SH-mediated reduction of extracellular murein hydrolase activity in these cells. Anti-biofilm assay showed that SH is poorly active against *S. aureus *grown in biofilm cultures, whereas SH diminished the amounts of extracellular DNA (eDNA) of *S. aureus* in a dose-dependent manner, which suggested that SH may impede biofilm formation by reducing the expression of *cidA* to inhibit autolysis and eDNA release in the early phase. Some of the microarray results were confirmed by real-time RT-PCR.

## 1. Intrduction

*Staphylococcus aureus *(*S. aureus*) is an important pathogen in both hospitals and the community that can cause a wide range of infections including sepsis, wound infections, pneumonia, and catheter-related infections. More than 60% of nosocomial *S. aureus* isolates are now resistant to methicillin, and some strains have developed resistance to more than 20 different antimicrobial agents in some countries [1]. One important character of *S. aureus* is the ability to form biofilms on damaged tissues and implanted biomaterials [2]. The biofilm structures are inherently resistant to many different antimicrobial agents and are difficult to eradicate from the infected host [3]. Thus, potential antimicrobial agents with new mechanisms of action and potent activity are widely investigated.

Sodium houttuyfonate (SH, decanoyl acetaldehyde sodium sulfite) is the addition compound of sodium bisulfite and houttuynin, which is the easily polymerized major constituent of the volatile oil of the plant *Houttuynia cordata* Thunb, a wild perennial herb with creeping rootstocks and swollen nodes [4]. SH is easily dissolved in hot water, slightly soluble in water and ethanol and insoluble in chloroform and benzene; it is soluble in alkaline solutions such as sodium hydroxide and potassium hydroxide where it simultaneously decomposes [5]. SH has been clinically used in China as an antimicrobial medicine for many years, and has been reported to effectively inhibit the growth of G^+^-bacteria such as *S. aureus*, *M. catarrhalis*, *B. influenzae*, and *S. pneumoniae* [6]. SH is primarily used by oral administration in clinical practice [5]. Fluorescence experiments indicated that houttuyfonate homologues (HOU-C_n_) directly bind to membrane proteins of G^+^-bacteria by hydrophobic interactions, and exert a stronger antibacterial activity in G^+^-bacteria than in G^−^-bacteria [7]. However, few reports are available that characterize the response mechanisms of *S. aureus* to SH using molecular biology approaches.

Recent reports have shown that the overexpression of *cidA*, a murein hydrolase regulator, may promote autolysis and extracellular DNA (eDNA) release to facilitate biofilm formation in *S. aureus in vitro* and *in vivo* [8]. Our preliminary experiments showed that SH inhibited Triton X-100-induced autolysis in *S. aureus*. Previous reports showed that treatment of *S. aureus *with chloramphenicol [9] and tetracycline [10] or vancomycin [11] and teicoplanin [12] led to decreased autolysis. On the contrary, β-lactam antibiotics can increase autolysis in *S. aureus*, presumably due to altered proteolytic processing [13]. Staphylococcal murein hydrolases include N-acetyl muramidase, N-acetyl glucosaminidase, N-acetylmuramyl-L-alanine amidase, endopeptidase and transglycosylases. These hydrolases degrade peptidoglycan saccules, resulting in cell lysis. If uncontrolled, these hydrolases can destroy the cell wall, resulting in cell lysis. Thus, we wanted to investigate whether SH reduces *S. aureus *biofilms *in vitro* by inhibiting autolysis. 

In this paper, we investigated the antimicrobial activity of SH against clinical and standard *S. aureus* strains grown in planktonic and bioﬁlm cultures, and we explored the molecular basis of the markedly reduced autolytic phenotype triggered by SH using transcriptomic analysis and validated the transcription of autolysis-related genes by real-time RT-PCR, and we used quantitative bacteriolytic assays to evaluate autolysis inhibition and we used propidium iodide (PI) staining to measure the inhibition of eDNA by treatment.

## 2. Results

### 2.1. Antimicrobial Activities of SH and Growth Curve of S. aureus under SH Stress

**Figure 1:**
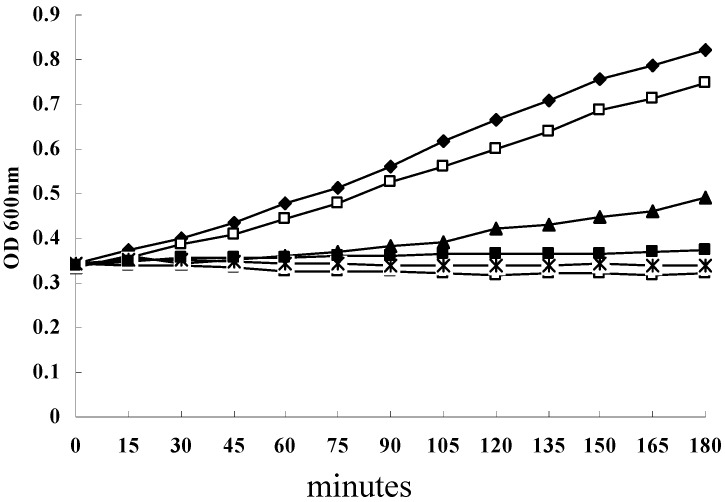
Growth curve for *S. aureus* strain ATCC 25923 in the presence or absence of SH. ◆, untreated *S. aureus*; □, *S. aureus* plus 4 µg/mL SH; ▲, *S. aureus* plus 8 µg/mL SH; ■, *S. aureus* plus 16 µg/mL SH; and *, *S. aureus* plus 32 µg/mL SH; △, *S. aureus* plus 64 µg/mL SH.

In this study, the MICs of SH against 21 *S. aureus* strains (17 of which are multidrug resistant) grown in planktonic cultures ranged from 4 to 128 µg/mL, and the MIC_90_ was 32 µg/mL. The MIC value of SH against *S. aureus* strain ATCC 25923 was 16 µg/mL (Table 1). Especially, against 16 of 17 multidrug resistant *S. aureus* strains tested, the MIC values of SH were lower than that of two conventional antimicrobial agents ciprofloxacin and oxacillin (Table 1), this result shows that SH is an effective antimicrobial agent against *S. aureus *grown in planktonic cultures. The MBIC and MBBC values of SH for 21 strains grown in biofilm cultures were all >1024 μg/mL, this result shows that SH is poorly active against *S. aureus *grown in biofilm cultures. The growth curve of *S. aureus* ATCC 25923 showed that SH concentrations of 16, 32 and 64 mg/L strongly inhibited the growth of *S. aureus* ATCC 25923 grown in planktonic cultures (Figure 1).

**Table 1:** Antibiograms of 21 *S. aureus* strainsusedin this study.

Strains	Source	Date of isolation (mo/yr)	Antibiotic resistances	VAN MIC (µg/mL)	CIP MIC (µg/mL)	OX MIC (µg/mL)	SH MIC (µg/mL)	SH MBIC (µg/mL)	SH MBBC (µg/mL)
SA003	Blood	2/2003	P	1	0.12	0.12	4	>1024	>1024
SA006	Skin	2/2003	M, P, CI, CM, E	1	64	128	16	>1024	>1024
SA009	Skin abscess	3/2003	M, CI, CM, E	0.5	128	256	16	>1024	>1024
SA017	Skin abscess	5/2003	P, T	2	0.5	0.5	4	>1024	>1024
SA018	Broncheal swab	6/2003	M, P, CI, CM, E, G	2	128	512	32	>1024	>1024
SA025	Sputum	10/2003	—	0.25	0.25	0.12	4	>1024	>1024
SA039	Broncheal swab	2/2004	M, P, CI, CM, E, G, RI, T, TMP/SXT	1	512	512	128	>1024	>1024
SA059	Broncheal swab	6/2004	M, P, CI, T, TMX/SXT	2	128	512	32	>1024	>1024
SA079	Sputum	9/2004	M, P, CI, CL, CM, E, G, TMP/SXT	4	128	256	64	>1024	>1024
SA092	Abscess	10/2004	M, P, OX, E	1	16	128	32	>1024	>1024
SA106	Wound isolate	1/2005	M, P, CI, CM, E, G, T, TMP/SXT	0.5	256	512	32	>1024	>1024
SA118	Blood	1/2005	M, P, E, G	1	64	256	8	>1024	>1024
SA121	Wound isolate	3/2005	M, P, CM, E, G	1	128	256	16	>1024	>1024
SA142	Abscess	5/2005	P, T, E	1	1	0.5	8	>1024	>1024
SA146	Skin abscess	5/2005	M, P, CL, CM, E, T	2	64	128	16	>1024	>1024
SA165	Urine	6/2006	P, CI, CM, E, G, RI, T	2	256	512	32	>1024	>1024
SA173	Broncheal swab	8/2005	M, P, T	0.25	32	128	8	>1024	>1024
SA179	Blood	9/2005	M, CI, CM, E	1	128	512	32	>1024	>1024
SA192	Urine	10/2005	M, P, CI, CM, E, G	1	64	512	32	>1024	>1024
SA203	Wound isolate	12/2005	M, P, CL, CM, T	2	64	512	16	>1024	>1024
ATCC25923	CMCC		—	1	0.25	0.25	16	>1024	>1024

Abbreviations: M, methicillin; P, penicillin; CI, clindamycin; CM, chloramphenicol; E, erythromycin; G, gentamicin; RI, rifampin; T, tetracycline; TMP/SXT, trimethoprimsulfamethoxazole; CT, cryptotanshinone; SH, sodium houttuyfonate; VAN, vancomycin; CIP, ciprofloxacin; OX, oxacillin.

### 2.2. Overview of SH-Triggered Transcriptional Profiles of S. aureus ATCC 25923 Cells

GeneChip analysis revealed that 764 genes were differentially regulated in response to SH challenge; 318 showed significant upregulation and 446 showed significant downregulation. Microarray-related data were submitted to the Gene Expression Omnibus (GEO) under the accession number GSE 13233. A complete list of all genes differentially expressed by SH can be found in the supplementary material (Table S1).

In previous studies, global transcriptional profiles have been used to evaluate autolysis genes, and their regulators [14,15,16]. In the present study, we compared genes differentially regulated by SH with those identified in the studies mentioned above.

### 2.3. Expression Levels of Autolysis-Associated Genes Treated by SH

We analyzed the expression levels of 67 genes printed on a microarray that were involved in autolysis (Table 2) or known as related autolysis regulators (Table S2). Some of the microarray results were evaluated by real-time RT-PCR (Table 2 and Table 3). 

**Table 2:** Microarray and real-time RT-PCR analysis of genes involved in autolysis affected by SH.

N315 SA no.	Gene	Description	Microarray *^a,c^*		RT-PCR *^b^*^,*c,d*^
			Fold change ± SD	*q* value (%)	Fold change ± SD
SA0905	*atl*	Bifunctional precursor autolysin (Atl)	−2.2 ± 0.5 **	0.11	−2.4 ± 0.8 ^§^
SA0423	*sle1*	N-Acetylmuramyl-L-alanine amidase	−12.4 ± 3.0 **	0.08	−31.2 ± 9.6 ^§§^
SA2329	*cidA*	Hypothetical protein, similar to transcription regulator	−2.0 ± 0.6 **	0.38	−3.7 ± 0.5 ^§^
SA0265	*lytM*	Peptidoglycan hydrolase	1.2 ± 0.6	29.61	1.0 ± 0.6
SA1090	*lytN*	LytN protein	−1.4 ± 0.5	9.33	−2.2 ± 0.9 ^§^
SA0252	*lrgA*	Murein hydrolase regulator LrgA	24.2 ± 5.2 **	0.00	80.4 ± 18.9 ^§§^
SA0253	*lrgB*	Antiholin-like protein LrgB	21.5 ± 6.4 **	0.00	76.5 ±14.7 ^§§^
SA0573	*sarA*	Staphylococcal accessory regulator A	1.8 ± 0.7 **	2.69	2.6 ± 0.9 ^§^
SA2328	*cidB*	Conserved hypothetical protein	1.5 ± 0.4 *	4.53	2.5 ± 1.0 ^§§^
SA2327	*cidC*	Pyruvate oxidase	2.4 ± 0.5 **	0.13	2.3 ±0.7 ^§^
SA1844	*agrA*	Accessory gene regulator A	−2.2 ± 0.7 **	0.19	−3.5 ± 0.6 ^§§^
SAS065	*RNAIII*	Delta hemolysin	−8.3 ± 1.4 **	0.00	−21.9 ± 5.3 ^§§^
SA0251	*lytR*	Two-component response regulator	−3.9 ± 0.9 **	0.00	−9.1 ± 1.4 ^§§^
SA0250	*lytS*	Two-component sensor histidine kinase	−1.9 ± 0.5 **	0.25	−2.4 ± 1.1 ^§^
SA0641	*mgrA*	Transcriptional regulator MgrA	−2.3 ± 0.8 **	0.11	−3.2 ± 0.9 ^§§^
SA1246	*arlS*	Sensor histidine kinase ArlS	−1.6 ± 0.4 **	0.83	−2.2 ± 0.7 ^§^
SA1248	*arlR*	Truncated (putative response regulator ArlR	−1.1 ± 0.5	29.61	−1.2 ± 0.5
SA0650	*norA*	Quinolone resistance protein	1.2 ± 0.38	27.61	ND
SA0904		Hypothetical protein, probable ATL autolysin transcription regulator	−1.5 ± 0.11 *	3.70	ND
SA1091	*eprH*	Endopeptidase resistance gene	−1.3 ± 0.10	13.98	ND

*^a^* Microarray data were analyzed using SAM, ** significantly differentially regulated genes after filtering at 5% FDR and fold change greater than 2; * Significantly differentially regulated genes after filtering at 5% FDR and fold change greater than 1.5; *^b^* The data of Real-time RT-PCR analysis of gene expression shown are the mean value of 2^−ΔΔ^^Ct^ ± standard deviation (SD), significant differences between three SH treatment samples and three control samples by *t*-test analysis are reported (^§^* P* < 0.05; ^§§^ P < 0.01); *^c^* − indicates reduction, and + indicates increase; *^d^* ND, not determined.

**Table 3:** Primers used in real-time RT-PCR with SYBR green probes

Primer	N315 ORF *^a^*	Sequence
16S rRNA for	SArRNA01	CGTGCTACAATGGACAATACAAA
16S rRNA rev	SArRNA01	ATCTACGATTACTAGCGATTCCA
atl for	N315-SA0905	TACCGTAACGGCGTAGGTCGT
atl rev	N315-SA0905	CATAGTCGTGTGTGTGTACGA
Sle1for	N315-SA0423	GTAGCCGTCCATCAACGAACT
Sle1 rev	N315-SA0423	CTATTGCTCGCAGCGTTACT
cidA for	N315-SA2329	CTTAGCCGGCAGTATTGTTG
cidA rev	N315-SA2329	TGAAGATAATGCAACGATAC
lytM for	N315-SA0265	ATGCCAATGGAAGCGGCCA
lytM rev	N315-SA0265	TTCGCATGACCACTAGCTGT
lytN for	N315-SA1090	GGAGACACACTTAGTGCT
lytN rev	N315-SA1090	CTAATGGTGTCATTGGCACT
lrgA for	N315-SA0252	CTGGTGCTGTTAAGTTAGGCG
lrgA rev	N315-SA0252	GTGACATAGCCAGTACAAAT
lrgB for	N315-SA0253	CGGTACAGTTGTAGCGTTATTA
lrgB rev	N315-SA0253	AGTGCTAATCCTCGGGCAATA
sarA for	N315-SA0573	CGCTGTATTGACATACATCAGCG
sarA rev	N315-SA0573	CTCGACTCAATAATGATTCGA
cidB for	N315-SA2328	GACGTCATTGTAACGTTATTGC
cidB rev	N315-SA2328	TGAACTAAATGCACCGGATTC
cidC for	N315-SA2327	GAGACTCAATCGACGCAGTTGT
cidC rev	N315-SA2327	TCCAAGTGCTGTACTATTCG
agrA for	N315-SA1844	TGAAATTCGTAAGCATGACCC
agrA rev	N315-SA1844	CATCGCTGCAACTTTGTAGAC
RNAIII for	N315-SAS065	ATGAGTTGTTTAATTTTAAGAAT
RNAIII rev	N315-SAS065	CACTGTGTCGATAATCCA
lytS for	N315-SA0250	GCATATGCGGTTGACCTCATAT
lytS rev	N315-SA0250	GCTTGTAATTGCTACGGCAGA
mgrA for	N315-SA0641	CAAGTTAATCGCTACTACTC
mgrA rev	N315-SA0641	CTTCACGTTGATCGACTTCG
arlS for	N315-SA1246	GTATATCATTGCGCTGGCA
arlS rev	N315-SA1246	GTGTTCGTAATTCATGTGACGC

*^a^* ORF, open reading frame.

Transcript levels of *atl*, *sle1*, and *cidA* were significantly decreased by 2.2, 12.4, and 2.0, respectively, whereas transcript levels of *lytN* were only slightly decreased by a factor of 1.4. The transcript levels of the positive regulators *agrA* and *RNAIII* were decreased by 2.2- and 8.3-fold, respectively. Expression of *lrgAB*, a negative regulator of autolysis, was significantly increased by 24.2- and 21.5-fold, and *sarA* was slightly increased 1.8-fold. Decreased *atl*, *sle1*, and *cidA *transcript levels were consistent with the induction of the autolytic repressors *lrgAB *and *sarA* and decreasedexpression of the positive regulators *agrA* and *RNAIII* in the SH-treated strain as compared to the control strain. This individually or collectively may contribute to the autolysis-inhibited phenotype. Real-time RT-PCR confirmed decreased *atl*, *sle1*, *cidA*, *agrA* and *RNAIII* mRNA levels and increased levels of *lrgAB* and *sarA* (Table 3).

*SceD* and *IsaA* share a conserved transglycosylase/muramidase domain, and *SsaA* (SA2093) and four structurally related proteins (SA0620, SA0710, SA2097, and SA2353) all share a common CHAP (cysteine, histidine-dependent amidohydrolase/peptidase) domain. All of these proteins have amino-terminal signal sequences, indicating that they are likely to be exported or targeted to the cell wall or membrane [17]. In present study, *sceD *(SA1898), *ssaA *(SA2093), *isaA *(SA2356), SA0620, SA2097, and SA2353 were downregulated by 3.0-, 7.8-, 11.5-, 5.9-, 13.3- and 5.8-fold, respectively, but we did not find any significant effects of SH on SA0710.

Expression of the protease genes *sspA*, *sspB*, *sspC*, *scpA*, *scpB*, and *htrA* was decreased by >2 fold. A *S. aureus *V8 protease, the major serine protease in *S. aureus*, is encoded by *sspA*. Loss of serine protease function has been shown to result in a pleiotropic effect on the profile of secreted proteins, including autolytic activity and proteolytic maturation of SspB (cysteine protease) [18]. Decreased levels of *sspA*, might result in altered processing of autolysins (e.g., Atl) that could affect autolytic activity [19]. The decreased transcription levels of protease-encoding genes suggest significant involvement in the autolytic changes triggered by SH.

We analyzed the expression of additional global regulators of autolysis exposed to SH. Transcript levels of *fmtA* were low in cells treated with SH and control strains. The *tagO* gene showed no significant changes in transcription levels, whereas *sarV* was upregulated by more than 3.2-fold. These genes were recently described as positive regulators of autolysis [20], although *fmtB* was only upregulated by 1.6-fold. There was a 1.5-fold decrease in the expression of *murE* (UDP-N-acetylmuramoylalanyl-D-glutamate-2,6-diaminopimelate ligase), and a 1.5-fold increase in that of *murI* (glutamate racemase) and *murZ *(functional homolog to *murA*). An enzyme that catalyzes further steps of peptidoglycan synthesis (*mraY*) was downregulated two-fold by SH treatment. We found that the expression of *rsbU rsbV*, *rsbW*, and *sigB* was significantly decreased by SH, that of their target genes *asp23 *and *sarS* was increased by 1.3-fold and decreased by 2.0-fold, respectively.

### 2.4. SH Decreases Triton X-100-Induced Autolysis

**Figure 2:**
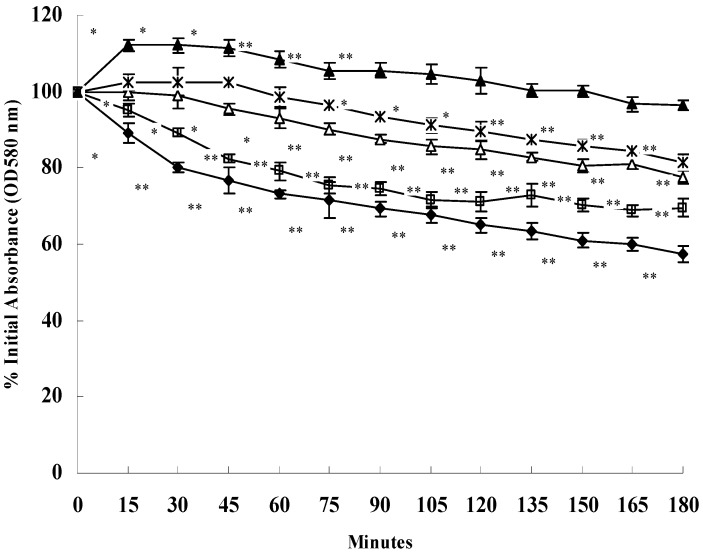
Effect of SH on Triton X-100-induced autolysis. Triton X-100 was used to stimulate autolysis in *S. aureus* ATCC 25923 cells grown in the absence or presence of various concentration of SH. The four concentration investigated were: ▲, 2× MIC; *, MIC; △, 1/2 MIC; □, 1/4 MIC; ◆, untreated. Data points are the mean ± SD of the mean of three replicate samples. * represents *P* < 0.05 (*t*-test analysis); ** represents *P *< 0.01 (*t*-test analysis), different from 0-minutes time points.

Since the results obtained from our microarray assay showed the expression levels of autolysis-associated genes were affected by SH treatment, we further determined the effect of SH on the autolytic activity of *S. aureus* ATCC 25923 in the presence of the non-ionic detergent Triton X-100. All cells from SH-exposed cultures showed a decreased rate of autolysis as compared to control cultures, and this inhibition was strengthened with increased concentrations of SH. After 3 h, a 30.4%, 22.2%, 18.6%, 3.5%, and 42.5% reduction of the original OD_600_ was observed for 1/4× MIC, 1/2× MIC, 1× MIC, 2× MIC, and no SH treatment, respectively. These findings indicate that Triton X-100-induced autolysis in *S. aureus* ATCC 25923 was inhibited by SH (Figure 2).

**Figure 3:**
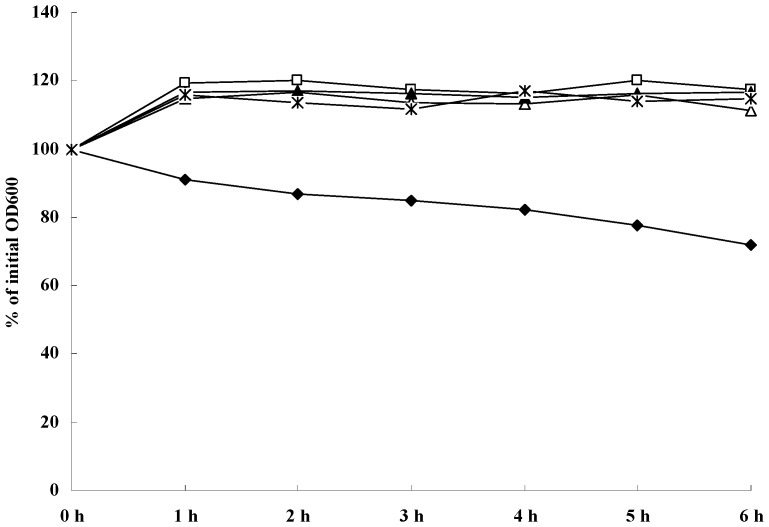
Quantitative analysis of extracellular bacteriolytic hydrolase activities in *S. aureus* ATCC 25923 cells treated with various concentration of SH. The four concentration investigated were □, 2× MIC; ▲, MIC; *, 1/2 MIC; △, 1/4 MIC; ◆, untreated.

Furthermore, quantitative bacteriolytic assays of extracellular proteins of *S. aureus *ATCC 25923 against lyophilized *S. aureus *suspensions were performed (Figure 3). SH treatment (1/4× MIC, 1/2× MIC, 1× MIC, and 2× MIC SH) of the extracellular murein hydrolases of *S. aureus* resulted in no significant decrease in the turbidity of *S. aureus *cells after 8 h of incubation, compared to extracellular murein hydrolases in untreated cells. These results demonstrate that SH reduces the extracellular murein hydrolase activity of *S. aureus*.

### 2.5. SH Alters the Cellular Peptidoglycan Hydrolase Profile

**Figure 4:**
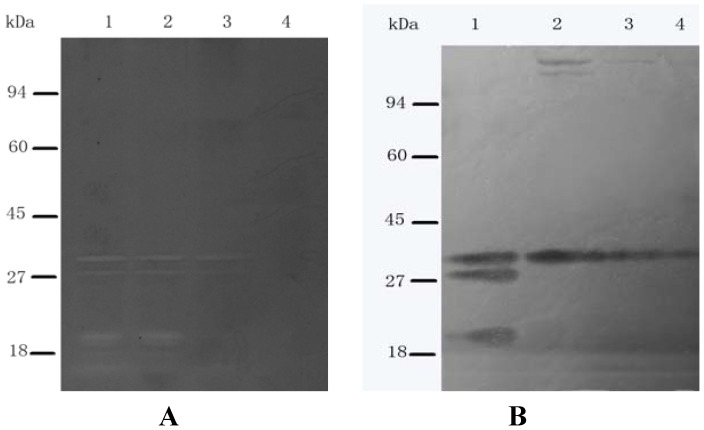
Zymographic analysis of bacteriolytic hydrolase activities of *S. aureus* ATCC 25923 cells treated with SH. LiCl (**A**) and SDS (**B**) autolysin extracts containing *S. aureus* ATCC 25923 cell walls treated with various concentrations of SH. Lane 1, no SH treatment; lane 2, 1/4× MIC SH; lane 3, 1/2× MIC SH; lane 4, 1× MIC SH. Molecular size markers are indicated on the left. The data shown are from a single representative experiment and were reproduced several times.

To investigate the mechanism of SH-inhibited autolysis, the peptidoglycan hydrolase profiles of SDS or LiCl autolysin extracts of *S. aureus* cell walls treated with 1/4× MIC, 1/2× MIC, or 1× MIC SH for 30 min were determined and compared with those of untreated ATCC 25923 cells. In LiCl autolysin extracts of *S. aureus* cell walls, 1/2× MIC SH treatment led to a significant decrease at 32 and two lower molecular weight bands. Additionally, MIC SH led to a complete loss of all 32 and two lower molecular weight bands Figure 4A. In the SDS group, 1/4× MIC SH treatment led to a slight induction at 143 kDa and 113 kDa; and a whole loss of two lower molecular weight bands; 1/2× MIC and MIC SH led to significant decrease in 143 kDa and 32 kDa, and a whole loss in estimated molecular sizes of 113 kDa and two lower molecular weight bands Figure 4B. These changes indicate altered proteolytic processing of Atl and other autolysins, and demonstrate that SH significantly inhibits autolysis, especially at concentrations >1/2× MIC SH.

### 2.6. Role of SH on DNA Release by *S. aureus*

**Figure 5:**
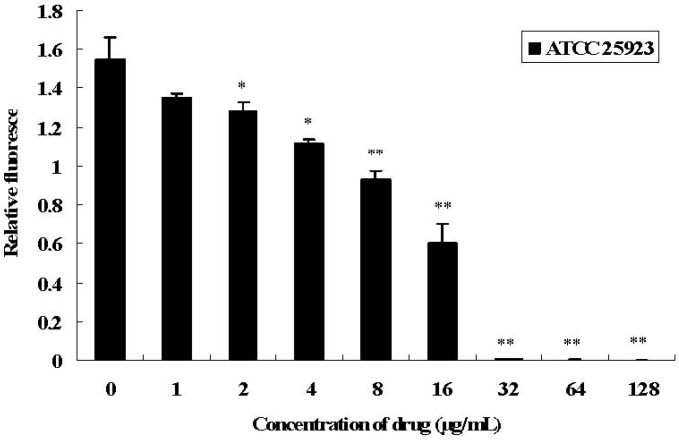
Role of SH on DNA release of *S. aureus*. The amount of eDNA stained by PI in *S. aureus* strain ATCC 25923 cultures treated with SH at concentration of 1/16× MIC, 1/8× MIC, 1/4× MIC, 1/2× MIC, 1× MIC and 2× MIC, 4× MIC, 8× MIC was measured. The drug-free culture was used as calibrator. Values represent the mean ± SD for three independent experiments. * represents *p *< 0.05 and ** represents *p *< 0.01.

The amounts of eDNA in the cultures of SH-treated (SH was added before biofilm formation) or control strains were measured by PI staining in microtiter plates. As shown in Figure 5, treatment with SH resulted in a dose-dependent decrease in the amount of eDNA in *S. aureus* and ATCC 25923. Compared with the level in cultures of control strain (without SH treatment), cultures with more than 1/8× MIC of SH displayed a significant reduction in the amount of eDNA for strains ATCC 25923; in the cultures with more than 2× MIC of SH, little or no eDNA could be detected in strains ATCC 25923.

## 3. Discussion

Transcriptional profiling and functional analysis revealed that SH treatment inhibits autolysis in *S. aureus*. Previous reports showed that oxacillin increased autolysis and led to decreased *atl *transcription, but did not affect on *cidA*, whereas tetracycline led to decreased autolysis and increase *atl *and *cidA* transcription [13]. These results indicate that the inhibition of autolysis by SH is different from that by vancomycin or tetracycline. Tetracycline, chloramphenicol and SH can lead to cell deathand lysis, which are believed to be separate and distinguishable events [13]. Surprisingly, microarray results showed SH significantly decreased the transcription levels of autolysin *cidA*, however, transcription of the *cidBC* autolysin gene showed a significant increase. We agree with Rice *et al*. that the *cidB* and *cidC* genes are also co-expressed as a transcript separate from *cidABC*, and this *cidBC* transcript is regulated by signals (*i.e*., *sigB*) that are independent of *cidABC* regulation [21].

In *S. aureus*, the regulation of autolytic activity is generally complex and can occur at a number of different levels including autolysin enzymatic activity, posttranslational processing of autolysins, and autolysin gene transcription [22]. The expression of virulence factors is controlled by a network of regulatory genes that can be grouped into two major classes: two-component systems (TCSs) and small transcription regulators. Virulence factor regulation is modulated by at least seven TCSs (ArlRS, SaeRS, AgrAC, SrrAB, LytRS, YycFG, and VraRS), the DNA-binding protein SarA, the SarA family of homologues (SarS, SarR, SarU, SarT, SarV, MgrA, and TcaR [13], and an alternative sigma factor (B). In addition, the SarA homologue Rot has been identified as a repressor of toxins; Rot and agr have opposing effects on selected target genes [23]. The two-component system *lytSR *is involved in regulation of peptidoglycan hydrolases. In *S. aureus*, a *lytS *mutant showed increased autolysis, altered levels of hydrolase activity, and a rough cell surface [24]. *lrgA *and *lrgB *are positively regulated by *lytSR*, and their products show similarities to a bacteriophage murein hydrolase transporter family of proteins known as holins; these proteins negatively affect peptidoglycan hydrolases [24]. Recent reports indicate a strong impact of ClpP proteases on virulence, stress response, and physiology in *S. aureus* [23]. In the present study, *clpP* was significantly induced by 3.5-fold. Recent DNA microarray analysis revealed a down-regulation of *lytSR *and *lrgAB *by ArlRS [26]. Moreover, the transcriptional regulator *mgrA (rat) *has been described as a repressor of autolysis and belongs to the MarR and SarA protein families [18]. In this study, *vraR*, *vraS*, *SarA*, *lrgA*, *lrgB*, *sarV*, and *clpP* were upregulated by >1.5 fold, whereas *agrA*, *RNAIII sarS*, *sigB*, *lytS*, *lytR arlS*, *agrC*, and *mgrA* were downregulated by >1.5 fold. Additionally, *saeR*, *saeS*, *yycF*, *yycG* and *tcaR *were slightly upregulated and *arlR*, *srrB*, and *srrA* were slightly downregulated, but *sarR*, *sarU*, and *sarT* were expressed at low levels. This suggests that SH affects the expression of two-component signal transduction systems and transcriptional regulators, and also suggests that the target genes of these regulators are differentially regulated.

As mentioned above, SH was poorly active against *S. aureus *grown in biofilm cultures (SH was added after 48 h of cultivation (mature phase of biofilm formation)), although SH**s**howed *in vitro* antibacterial activity against *S. aureus* grown in planktonic cultures (SH was added before biofilm formation), whereas SH diminished the amounts of eDNA of *S. aureus* in a dose-dependent manner during biofilm formation. Indeed, eDNA is an essential matrix molecule that is produced during biofilm development by many bacterial species, including *P. aeruginosa* and *S. aureus* [27], and the overexpression of *cidA* may promote autolysis and eDNA release [28]. We concluded that SH may impede biofilm formation by reducing eDNA release in the early phase, and SH has no anti-biofilm effect in the mature phase of biofilm formation.

Taken together, the *in vitro* antibacterial activities and growth curve experiments showed that SH significantly inhibited the growth of *S. aureus*, simultaneously, SH diminished the amounts of eDNA of *S. aureus* in a dose-dependent manner during biofilm formation, our results suggests that SH may contribute to the antimicrobial effect by inhibition of autolysis of the *S. aureus*, and this may be part of the mode of action of SH.

## 4. Experimental

### 4.1. Bacterial Strains and Materials

*S. aureus* strains included 20 clinical *S. aureus* isolates from the First Hospital of Jilin University that had different antimicrobial susceptibility patterns (Table 1), as well as the standard strain ATCC25923, which was obtained from the China Medical Culture Collection Center (CMCC). Mueller-Hinton broth II (MHB II) was purchased from BD Biosciences, Inc. (Sparks, MD, USA). SH was purchased from CMCC, and stock solutions of varying concentrations were prepared in dimethyl sulfoxide (DMSO, Sigma-Aldrich, St. Louis, MO, USA).

### 4.2. Antibiotic Susceptibility Testing

The minimum inhibitory concentrations (MICs) of SH against the 21 *S. aureus* strains were determined in triplicate by broth microdilution or macrodilution, using two-fold serial dilutions in MHB II according to standard CLSI procedures [29]. MICs were defined as the lowest concentration at which no visible growth was observed. The minimum concentration of SH that inhibited 90% of the isolates tested was defined as the MIC_90_.

### 4.3. Growth Curves

Growth curves of different concentrations of SH against *S. aureus* strain ATCC 25923 were assayed according previous study [30].

### 4.4. Treatment with SH for Microarrays

*S. aureus* strain ATCC 25923 was grown overnight at 200 rpm in a rotary shaker at 37 °C in MHB II (10 mL). Two 250 mL Erlenmeyer flasks, each containing MHB II (100 mL), were cultured overnight to an initial OD_600_ of 0.05. Bacteria were grown at 37 °C and 200 rpm to an OD_600_ of 0.3. Subsequently, an aliquot (62.5 µL) of a 12,800 µg/mL SH stock solution prepared in dimethyl sulfoxide (DMSO) was added to one of the cultures (experimental culture), giving a final concentration of 1/2× MIC. Hence, the final concentration of solvent was 1% (vol/vol) DMSO; this amount of DMSO did not alter the pH of the medium. The other culture containing 1% (vol/vol) DMSO without SH was used as the control. All bacterial suspensions (both experimental and control) were further incubated for 30 min at 37 °C [31]. RNA isolation was then performed at this time. Three independent bacterial cultures for each SH treatment or control condition were prepared as biological replicates for RNA isolation on different days.

### 4.5. RNA Isolation and cDNA Labeling

Bacterial cells were treated with RNA Protect bacterial reagent (QIAGEN, Inc., Valencia, CA, USA) to minimize RNA degradation immediately before harvesting. Cells were collected by centrifugation and kept at −80 °C. RNA isolation and cDNA labeling were carried out as described previously [30]. Three independent RNA preparations and cDNA labelings were carried out on different days.

GeneChip *S. aureus* genome array (antisense) used here was provided by CapitalBio Corporation (http://www.capitalbio.com/en/index.asp, Beijing, China), a service provider authorized by Affymetrix Inc. (Santa Clara, CA, USA). This GeneChip includes N315, Mu50, NCTC 8325, and COL. The array contains probe sets to over 3,300 *S. aureus* ORFs and over 4,800 intergenic regions. GeneChip hybridization, washing, staining and scanning were performed according to previously described techniques [30].

Images were processed with Microarray Analysis Suite 5.0 (Affymetrix). The raw data from the array scans were normalized against median-centering genes for each array, and then log transformed. Differentially expressed genes were identified using Affymetrix GeneChip Operating Software (GCOS, Ver.1.0), which uses statistical criteria to generate a “present” or “absent” call for genes represented by each probe set in the array. Additionally, genes with ‘absent’ scores were filtered out and the remaining genes were analyzed. To identify genes differentially expressed in RH samples as compared to controls, Significance Analysis of Microarrays (SAM) software [32] was used. SAM one-class option with estimated false discovery rate (FDR) <5% and fold change cutoff of 2 was used to identify significantly differentially regulated genes between three SH treatment samples and three control samples; SAM two-class option with estimated FDR <5% and fold change cutoff of 1.5 was used to identify significantly differentially regulated genes involved in autolysis affected by SH between three SH treatment samples and three control samples [33]. 

### 4.6. Quantitative Real-Time RT-PCR

Quantitative real-time reverse transcription (RT)-PCR was used to verify the microarray results. Aliquots of the RNA preparations from SH untreated and control samples used in the microarray experiments were saved for quantitative real-time RT-PCR follow-up studies. Quantitative real time PCR was performed in triplicate using the 7000 Sequence Detection System (Applied Biosystems, Foster City, CA, USA) according to a previously described procedure [30]. The relative levels of expression of the genes of interest were calculated using the 2^−ΔΔ^^Ct^ method described by Livak and Schmittgen [34], where Ct is the fractional threshold cycle. The Ct obtained for each gene of interest was normalized with that of 16S rRNA. The statistical significance of the fold differences in expression levels of the genes of interest was calculated using the two-tailed t-test analysis. A minimum 2-fold difference and a *P* value of <0.05 were considered significant between three SH treatment samples and three control samples, unless otherwise stated [35,36].

### 4.7. Triton X-100-Induced Autolysis

*S. aureus *strain ATCC 25923 was grown to an optical density of 0.3 at 600 nm (OD_600_), at which point SH was added to the cultures, typically at concentrations of 1/4× MIC, 1/2× MIC, MIC, or 2× MIC. Growth with shaking at 37 °C was allowed to continue to OD_580_ = 0.7 in control cultures, and antibiotic-exposed cultures were incubated for the same length of time as the control cultures. The cells were harvested by centrifugation and washed once by re-suspension in cold distilled water. The cell pellet was re-suspended to an OD_600_ of 1.0 in 0.05M Tris-HCl pH 7.0 containing 0.05% (v/v) Triton X-100. The cell suspension was incubated at 30 °C with shaking and the OD_580_ was determined at various intervals [37].

### 4.8. Quantitative Bacteriolytic Hydrolase Assays

Bacteriolysis of lyophilized *S. aureus *by extracellular hydrolases was quantified as previously described [38]. Equivalent amounts (100 µg) of concentrated supernatants of strain ATCC 25923 from various concentrations of drug treatment or control were added to a suspension of autoclaved and lyophilized *S. aureus* ATCC 25923 (1 mg/mL) in 100 mM Tris-HCl (pH 8.0) and incubated at 37 °C with shaking.Lytic activity was recorded by measuring the progressive decrease in absorbance (OD_600_).

### 4.9. Autolytic Enzyme Extracts

Cultures of *S. aureus* ATCC 25923 were grown to mid-exponential phase in TSB (250 mL) at 37 °C with aeration, at which time MOL was added to the cultures, typically at concentrations of 1/4× MIC, 1/2× MIC, 1× MIC or 2× MIC. The cultures were incubated with shaking at 37 °C for another 30 min, after which they were rapidly chilled, harvested by centrifugation, washed once in ice-cold 50 mM Tris-Cl, pH 7.5, and extracted with 4% sodium dodecyl sulfate (SDS, 250 μL) at room temperature or 3 M LiCl at 4 °C for 30 min with stirring. Protein concentrations were determined using the BCA protein assay kit (Pierce, Rockford, IL, USA) with bovine serum albumin as the standard [39].

### 4.10. Preparation of Crude Cell Walls

Cultures of *S. aureus* ATCC 25923 were grown in TSB to mid-logarithmic phase at 37 °C with aeration, after which they were rapidly chilled, harvested by centrifugation and resuspended in boiling 8% SDS. After boiling for 30 min, the samples were washed in distilled water to remove the SDS, mechanically disrupted, washed again and lyophilized.

### 4.11. Bacteriolytic Enzyme Profiles after SDS-Polyacrylamide Gel Electrophoresis

The separation of proteins was carried out using the technique of Laemmli [40]. Resolving gels (7.5% acrylamide–0.2% bisacrylamide) contained crude cell walls (1 mg dry weight per mL). Samples were separated by electrophoresis at a constant current of 20 mA at room temperature until the blue dye reached the bottom of the separating gel. The visualization of bacteriolytic enzymes was carried out as follows: the gels were initially washed in distilled water four times for 15 min each, washed in buffer composed of 50 mM Tris-Cl (pH 7.5), 0.1% Triton X-100, 10 mM CaCl_2_ and 10 mM MgCl_2_ and then incubated for 24 h at 37 °C with gentle agitation in the same buffer as described above [41].

### 4.12. Establishment of Microbial Biofilms

For each microorganism, the OD of the overnight suspension was determined at 600 nm and diluted in MHB as required to a ﬁnal concentration of 1 × 10^5^ cfu/mL. Using previously established optimum conditions for bioﬁlm development (data not shown) bioﬁlms of each microorganism were grown in clear ﬂat-bottomed, tissue culture-treated 6-well microtitre plates. To conﬁrm slime production, microorganisms were cultured onto Congo Red agar [42]. Conﬂuent biofilms were subsequently generated in wells of white-walled, clear bottom, tissue culture-treated 96-well microtitre plates, inoculated with a 1 × 10^5^ cfu/mL suspension of each microorganism (200 µL). Empty wells were reserved for use as negative controls. Microtitre plates were then incubated for 48 h at 37 °C.

### 4.13. Biofilm Antimicrobial Susceptibility Testing

Wells containing bioﬁlm were gently washed with PBS (250 µL) to remove any unbound cells. Serial double dilutions of SH were prepared in either MHB (SH 0.25~1024 µg/mL). In triplicate wells, MHB (100 µL) was added to antimicrobial agent (100 µL) in decreasing concentrations across the rows. After 24 h of incubation at 37 °C, the antimicrobial agents were removed and the wells washed once with PBS. PBS (250 µL) was added to each well and the plate sonicated at 50 Hz in a water bath for 30 min at room temperature. Bioﬁlms were recovered from the wells by a scrape and wash procedure [43] and the entire contents of the well (250 µL) mixed with MHA, cooled to 50 °C. Following incubation of the set plates at 37 °C for 24 h, The minimum biofilm inhibition concentration (MBIC) was determined as the lowest concentration to show growth below or equal to that of the control (bioﬁlm in saline). The minimum biofilm bactericidal concentration (MBBC) was identiﬁed as the lowest concentration demonstrating no bacterial growth.

### 4.14. Measurement of eDNA in Microtiter Plate Cultures Treated with SH

Overnight cultures of *S. aureus* ATCC 25923 grown in TSB medium containing 0.25% glucose were diluted to an OD_600_ of 0.001 in AB medium supplemented with 0.5% glucose, 0.05 mM PI and 10% TSB, and SH (dissolved in DMSO) was added to eight of the cultures to obtain final concentrations of 1/16× MIC, 1/8× MIC, 1/4× MIC, 1/2× MIC, 1× MIC and 2× MIC, 4× MIC, 8× MIC, respectively. These cultures were transferred to the wells of polystyrene microtiter plates (150 mL per well) and incubated for 24 h at 37 °C, upon which the PI absorbance was measured at 480 nm [44].

## 5. Conclusion

In summary, SH inhibited autolysis in *S. aureus*, presumably due to altered proteolytic processing. To our knowledge, this is the first report regarding the effects of SH on autolysis in *S. aureus*; this lays the groundwork for further study of the action mechanism of SH.

## Supplementary Materials

Supplementary materials can be accessed at: http://www.mdpi.com/1420-3049/16/10/8848/s1.

## Supplementary Materials

Supplementary File 1
