# Supplementary material for: Transcriptional and Functional Analysis Shows Sodium Houttuyfonate-Mediated Inhibition of Autolysis in Staphylococcus aureus
Source: Molecules. 2011 Oct 21;16(10):8848–65. doi: 10.3390/molecules16108848 (PMC6264171; doi:10.3390/molecules16108848)
Supplement: Supplementary File 1 [file molecules-16-08848-s001.doc]

**Supplementary Material**

**Table S1. Genes with expression changes of at least twofold in *S. aureus* ATCC25923 exposed to sodium houttuyfonate.**

| **ORF no. *^a,b^*** | **Gene** | **Product or putative function** | **Fold change***^c,d^* | ***q* value (%)** | **Functional category** |
| --- | --- | --- | --- | --- | --- |
| SA2405 | *betA* | Choline dehydrogenase | -33.0 | 0.00 | [Adaption to atypical conditions](http://www.bio.nite.go.jp/dogan/GeneSearchResult?GENE_LIST_TYPE=1&type=504&GENOME_LIST=n315G1&CLASS_ID=34.01&WITH_GENE_MAP=1) |
| SA2406 | *gbsA* | Glycine betaine aldehyde dehydrogenase gbsA | -14.9 | 0.00 | [Adaption to atypical conditions](http://www.bio.nite.go.jp/dogan/GeneSearchResult?GENE_LIST_TYPE=1&type=504&GENOME_LIST=n315G1&CLASS_ID=34.01&WITH_GENE_MAP=1) |
| SA0755 |  | Hypothetical protein, similar to general stress protein 170 | -2.2 | 0.13 | [Adaption to atypical conditions](http://www.bio.nite.go.jp/dogan/GeneSearchResult?GENE_LIST_TYPE=1&type=504&GENOME_LIST=n315G1&CLASS_ID=34.01&WITH_GENE_MAP=1) |
| SA0144 | *capA* | Capsular polysaccharide synthesis enzyme Cap5A | -2.1 | 0.13 | [Adaption to atypical conditions](http://www.bio.nite.go.jp/dogan/GeneSearchResult?GENE_LIST_TYPE=1&type=504&GENOME_LIST=n315G1&CLASS_ID=34.01&WITH_GENE_MAP=1) |
| SA1408 | *dnaJ* | DnaJ protein (HSP40) | 2.1 | 0.38 | [Adaption to atypical conditions](http://www.bio.nite.go.jp/dogan/GeneSearchResult?GENE_LIST_TYPE=1&type=504&GENOME_LIST=n315G1&CLASS_ID=34.01&WITH_GENE_MAP=1) |
| SA1096 | *clpQ* | Heat shock protein HslV | 2.2 | 0.19 | [Adaption to atypical conditions](http://www.bio.nite.go.jp/dogan/GeneSearchResult?GENE_LIST_TYPE=1&type=504&GENOME_LIST=n315G1&CLASS_ID=34.01&WITH_GENE_MAP=1) |
| SA1549 |  | Hypothetical protein, similar to serine proteinase Do, heat-shock protein htrA | 2.6 | 0.11 | [Adaption to atypical conditions](http://www.bio.nite.go.jp/dogan/GeneSearchResult?GENE_LIST_TYPE=1&type=504&GENOME_LIST=n315G1&CLASS_ID=34.01&WITH_GENE_MAP=1) |
| SA1410 | *grpE* | GrpE protein (HSP-70 Cofactor HSP20) | 2.9 | 0.00 | [Adaption to atypical conditions](http://www.bio.nite.go.jp/dogan/GeneSearchResult?GENE_LIST_TYPE=1&type=504&GENOME_LIST=n315G1&CLASS_ID=34.01&WITH_GENE_MAP=1) |
| SA1146 | *bsaA* | Glutathione peroxidase | 3.2 | 0.06 | [Adaption to atypical conditions](http://www.bio.nite.go.jp/dogan/GeneSearchResult?GENE_LIST_TYPE=1&type=504&GENOME_LIST=n315G1&CLASS_ID=34.01&WITH_GENE_MAP=1) |
| SA0723 | *clpP* | ATP-dependent Clp protease proteolyticsubunit homologue | 3.5 | 0.00 | [Adaption to atypical conditions](http://www.bio.nite.go.jp/dogan/GeneSearchResult?GENE_LIST_TYPE=1&type=504&GENOME_LIST=n315G1&CLASS_ID=34.01&WITH_GENE_MAP=1) |
| SA0483 | *clpC* | Endopeptidase | 3.8 | 0.00 | [Adaption to atypical conditions](http://www.bio.nite.go.jp/dogan/GeneSearchResult?GENE_LIST_TYPE=1&type=504&GENOME_LIST=n315G1&CLASS_ID=34.01&WITH_GENE_MAP=1) |
| SA0483 | *clpC* | Endopeptidase | 4.0 | 0.00 | [Adaption to atypical conditions](http://www.bio.nite.go.jp/dogan/GeneSearchResult?GENE_LIST_TYPE=1&type=504&GENOME_LIST=n315G1&CLASS_ID=34.01&WITH_GENE_MAP=1) |
| SA0835 | *clpB* | ClpB chaperone homologue | 8.0 | 0.00 | [Adaption to atypical conditions](http://www.bio.nite.go.jp/dogan/GeneSearchResult?GENE_LIST_TYPE=1&type=504&GENOME_LIST=n315G1&CLASS_ID=34.01&WITH_GENE_MAP=1) |
| SA1941 | *dps* | General stress protein 20U | 8.4 | 0.00 | [Adaption to atypical conditions](http://www.bio.nite.go.jp/dogan/GeneSearchResult?GENE_LIST_TYPE=1&type=504&GENOME_LIST=n315G1&CLASS_ID=34.01&WITH_GENE_MAP=1) |
| SA0173 |  | Hypothetical protein, similar to surfactin synthetase | 2.2 | 0.40 | [Antibiotic production](http://www.bio.nite.go.jp/dogan/GeneSearchResult?GENE_LIST_TYPE=1&type=504&GENOME_LIST=n315G1&CLASS_ID=34.03&WITH_GENE_MAP=1) |
| SA0905 | *atl* | Autolysin (N-acetylmuramyl-L-alanine amidase and endo-b-N-acetylglucosaminidase) | -2.2 | 0.11 | [Cell division](http://www.bio.nite.go.jp/dogan/GeneSearchResult?GENE_LIST_TYPE=1&type=504&GENOME_LIST=n315G1&CLASS_ID=31.07&WITH_GENE_MAP=1) |
| SA0423 | *sle1* | Hypothetical protein, similar to autolysin (N-acetylmuramyl-L-alanine amidase) | -12.4 | 0.00 | [Cell wall](http://www.bio.nite.go.jp/dogan/GeneSearchResult?GENE_LIST_TYPE=1&type=504&GENOME_LIST=n315G1&CLASS_ID=31.01&WITH_GENE_MAP=1) |
| SA0793 | *dltA* | D-alanine-D-alanyl carrier protein ligase | -6.5 | 0.00 | [Cell wall](http://www.bio.nite.go.jp/dogan/GeneSearchResult?GENE_LIST_TYPE=1&type=504&GENOME_LIST=n315G1&CLASS_ID=31.01&WITH_GENE_MAP=1) |
| SA0795 | *dltC* | D-alanyl carrier protein | -5.7 | 0.00 | [Cell wall](http://www.bio.nite.go.jp/dogan/GeneSearchResult?GENE_LIST_TYPE=1&type=504&GENOME_LIST=n315G1&CLASS_ID=31.01&WITH_GENE_MAP=1) |
| SA1458 | *lytH* | N-acetylmuramoyl-L-alanine amidase | -3.1 | 0.00 | [Cell wall](http://www.bio.nite.go.jp/dogan/GeneSearchResult?GENE_LIST_TYPE=1&type=504&GENOME_LIST=n315G1&CLASS_ID=31.01&WITH_GENE_MAP=1) |
| SA2199 | *fmhA* | FmhA protein | -2.0 | 0.13 | [Cell wall](http://www.bio.nite.go.jp/dogan/GeneSearchResult?GENE_LIST_TYPE=1&type=504&GENOME_LIST=n315G1&CLASS_ID=31.01&WITH_GENE_MAP=1) |
| SA1291 |  | Hypothetical protein, similar to lipopolysaccharide biosynthesis-related pr homolog | 2.2 | 0.19 | [Cell wall](http://www.bio.nite.go.jp/dogan/GeneSearchResult?GENE_LIST_TYPE=1&type=504&GENOME_LIST=n315G1&CLASS_ID=31.01&WITH_GENE_MAP=1) |
| SA2316 | *srtA* | Sortase | 3.3 | 0.06 | [Cell wall](http://www.bio.nite.go.jp/dogan/GeneSearchResult?GENE_LIST_TYPE=1&type=504&GENOME_LIST=n315G1&CLASS_ID=31.01&WITH_GENE_MAP=1) |
| SA1691 | *sgtB* | Hypothetical protein, similar to penicillin-binding protein 1A/1B | 4.9 | 0.00 | [Cell wall](http://www.bio.nite.go.jp/dogan/GeneSearchResult?GENE_LIST_TYPE=1&type=504&GENOME_LIST=n315G1&CLASS_ID=31.01&WITH_GENE_MAP=1) |
| SA2354 |  | Hypothetical protein, similar to acyltransferase | -5.9 | 0.00 | [Cell wall](http://www.bio.nite.go.jp/dogan/GeneSearchResult?GENE_LIST_TYPE=1&type=504&GENOME_LIST=n315G1&CLASS_ID=31.01&WITH_GENE_MAP=1) |
| SA2230 |  | Hypothetical protein, similar to beta-lactamase | 2.4 | 0.13 | [Cell wall](http://www.bio.nite.go.jp/dogan/GeneSearchResult?GENE_LIST_TYPE=1&type=504&GENOME_LIST=n315G1&CLASS_ID=31.01&WITH_GENE_MAP=1) |
| SA0875 |  | Hypothetical protein, similar to cell wall synthesis protein | 2.8 | 0.06 | [Cell wall](http://www.bio.nite.go.jp/dogan/GeneSearchResult?GENE_LIST_TYPE=1&type=504&GENOME_LIST=n315G1&CLASS_ID=31.01&WITH_GENE_MAP=1) |
| SA2316 | *srtA* | Sortase | 3.3 | 0.06 | [Cell wall](http://www.bio.nite.go.jp/dogan/GeneSearchResult?GENE_LIST_TYPE=1&type=504&GENOME_LIST=n315G1&CLASS_ID=31.01&WITH_GENE_MAP=1) |
| SA0205 |  | Hypothetical protein, similar to lysostaphin precursor | 14.9 | 0.00 | [Cell wall](http://www.bio.nite.go.jp/dogan/GeneSearchResult?GENE_LIST_TYPE=1&type=504&GENOME_LIST=n315G1&CLASS_ID=31.01&WITH_GENE_MAP=1) |
| SAV1017  SA0875 | *ypfP* | Hypothetical protein,similar to cell wall synthesis protein | 2.8 | 0.06 | Cell wall |
| SA0595 | *tagB* | Teichoic acid biosynthesis protein B | 2.2 | 0.20 | Cell wall |
| SA1025 | *mraY* | Phospho-N-muramic acid-pentapeptide translocase | -2.0 | 0.20 | Cell wall |
| SA2124 | *fosB* | Fosfomycin resistance protein fofB – Staphylococcus sp. Plasmid | -2.6 | 0.07 | [Detoxification](http://www.bio.nite.go.jp/dogan/GeneSearchResult?GENE_LIST_TYPE=1&type=504&GENOME_LIST=n315G1&CLASS_ID=34.02&WITH_GENE_MAP=1) |
| SA1238 |  | Hypothetical protein, similar to tellurite resistance protein | 2.6 | 0.06 | [Detoxification](http://www.bio.nite.go.jp/dogan/GeneSearchResult?GENE_LIST_TYPE=1&type=504&GENOME_LIST=n315G1&CLASS_ID=34.02&WITH_GENE_MAP=1) |
| SA0312 |  | Hypothetical protein, similar to alkanal monooxygenase alpha chain | 4.0 | 0.00 | [Detoxification](http://www.bio.nite.go.jp/dogan/GeneSearchResult?GENE_LIST_TYPE=1&type=504&GENOME_LIST=n315G1&CLASS_ID=34.02&WITH_GENE_MAP=1) |
| SA0878 |  | Toxic anion resistance protein homologue | 4.4 | 0.00 | [Detoxification](http://www.bio.nite.go.jp/dogan/GeneSearchResult?GENE_LIST_TYPE=1&type=504&GENOME_LIST=n315G1&CLASS_ID=34.02&WITH_GENE_MAP=1) |
| SA1853 |  | Hypothetical protein, 2imilar to DNA mismatch repair protein MutS | -6.1 | 0.00 | [DNA modification and repair](http://www.bio.nite.go.jp/dogan/GeneSearchResult?GENE_LIST_TYPE=1&type=504&GENOME_LIST=n315G1&CLASS_ID=33.02&WITH_GENE_MAP=1) |
| SA0391 | *hsdM* | Probable type I site-specific deoxyribonuclease (EC 3.1.21.3) LldI chain hsdM [Pathogenicity island SaPIn2] | -4.0 | 0.00 | [DNA modification and repair](http://www.bio.nite.go.jp/dogan/GeneSearchResult?GENE_LIST_TYPE=1&type=504&GENOME_LIST=n315G1&CLASS_ID=33.02&WITH_GENE_MAP=1) |
| SA0392 | *hsdS* | Probable restriction modification system specificity subunit [Pathogenicity island SaPIn2] | -3.2 | 0.00 | [DNA modification and repair](http://www.bio.nite.go.jp/dogan/GeneSearchResult?GENE_LIST_TYPE=1&type=504&GENOME_LIST=n315G1&CLASS_ID=33.02&WITH_GENE_MAP=1) |
| SA1386 |  | Hypothetical protein, similar to endonuclease IV | -3.0 | 0.00 | [DNA modification and repair](http://www.bio.nite.go.jp/dogan/GeneSearchResult?GENE_LIST_TYPE=1&type=504&GENOME_LIST=n315G1&CLASS_ID=33.02&WITH_GENE_MAP=1) |
| SA1138 | *mutL* | DNA mismatch repair protein | -2.9 | 0.00 | [DNA modification and repair](http://www.bio.nite.go.jp/dogan/GeneSearchResult?GENE_LIST_TYPE=1&type=504&GENOME_LIST=n315G1&CLASS_ID=33.02&WITH_GENE_MAP=1) |
| SA0538 | *ung* | Uracil-DNA glycosylase | -2.8 | 0.00 | [DNA modification and repair](http://www.bio.nite.go.jp/dogan/GeneSearchResult?GENE_LIST_TYPE=1&type=504&GENOME_LIST=n315G1&CLASS_ID=33.02&WITH_GENE_MAP=1) |
| SA0189 | *hsdR* | Probable type I restriction enzyme restriction chain | -2.8 | 0.00 | [DNA modification and repair](http://www.bio.nite.go.jp/dogan/GeneSearchResult?GENE_LIST_TYPE=1&type=504&GENOME_LIST=n315G1&CLASS_ID=33.02&WITH_GENE_MAP=1) |
| SA2335 | *adaB* | Probable methylated DNA-protein cysteine methyltransferase | 2.0 | 0.61 | [DNA modification and repair](http://www.bio.nite.go.jp/dogan/GeneSearchResult?GENE_LIST_TYPE=1&type=504&GENOME_LIST=n315G1&CLASS_ID=33.02&WITH_GENE_MAP=1) |
| SA1285 | *nth* | Endonuclease-like protein | 2.7 | 0.06 | [DNA modification and repair](http://www.bio.nite.go.jp/dogan/GeneSearchResult?GENE_LIST_TYPE=1&type=504&GENOME_LIST=n315G1&CLASS_ID=33.02&WITH_GENE_MAP=1) |
| SA0827 |  | Hypothetical protein, similar to ATP-dependent nuclease subunit B | 2.8 | 0.06 | [DNA modification and repair](http://www.bio.nite.go.jp/dogan/GeneSearchResult?GENE_LIST_TYPE=1&type=504&GENOME_LIST=n315G1&CLASS_ID=33.02&WITH_GENE_MAP=1) |
| SA1711 |  | Hypothetical protein, similar to DNA-damage inducible protein P | 11.6 | 0.00 | [DNA modification and repair](http://www.bio.nite.go.jp/dogan/GeneSearchResult?GENE_LIST_TYPE=1&type=504&GENOME_LIST=n315G1&CLASS_ID=33.02&WITH_GENE_MAP=1) |
| SA2278 |  | Hypothetical protein, imilar to mutator protein mutT | -2.1 | 0.19 | [DNA modification and repair](http://www.bio.nite.go.jp/dogan/GeneSearchResult?GENE_LIST_TYPE=1&type=504&GENOME_LIST=n315G1&CLASS_ID=33.02&WITH_GENE_MAP=1) |
| SA1137 | *mutS* | DNA mismatch repair protein | -2.1 | 0.13 | [DNA modification and repair](http://www.bio.nite.go.jp/dogan/GeneSearchResult?GENE_LIST_TYPE=1&type=504&GENOME_LIST=n315G1&CLASS_ID=33.02&WITH_GENE_MAP=1) |
| SA1489 | *tag* | DNA-3-methyladenine glycosidase | 2.3 | 0.20 | [DNA modification and repair](http://www.bio.nite.go.jp/dogan/GeneSearchResult?GENE_LIST_TYPE=1&type=504&GENOME_LIST=n315G1&CLASS_ID=33.02&WITH_GENE_MAP=1) |
| SA1092 |  | Hypothetical protein, similar to DNA processing Smf protein | -3.5 | 0.00 | [DNA packaging and segregation](http://www.bio.nite.go.jp/dogan/GeneSearchResult?GENE_LIST_TYPE=1&type=504&GENOME_LIST=n315G1&CLASS_ID=33.04&WITH_GENE_MAP=1) |
| SA1189 | *parC* | Topoisomerase IV subunit A | -2.6 | 0.00 | [DNA packaging and segregation](http://www.bio.nite.go.jp/dogan/GeneSearchResult?GENE_LIST_TYPE=1&type=504&GENOME_LIST=n315G1&CLASS_ID=33.04&WITH_GENE_MAP=1) |
| SA1462 |  | Hypothetical protein, similar to single-strand DNA-specific exonuclease | -5.5 | 0.00 | [DNA recombination](http://www.bio.nite.go.jp/dogan/GeneSearchResult?GENE_LIST_TYPE=1&type=504&GENOME_LIST=n315G1&CLASS_ID=33.03&WITH_GENE_MAP=1) |
| SA1070 | *recG* | ATP-dependent DNA helicase | 2.6 | 0.06 | [DNA recombination](http://www.bio.nite.go.jp/dogan/GeneSearchResult?GENE_LIST_TYPE=1&type=504&GENOME_LIST=n315G1&CLASS_ID=33.03&WITH_GENE_MAP=1) |
| SA1720 | *lig* | DNA ligase (polydeoxyribonucleotide syntase [NAD+]) | -2.2 | 0.19 | DNA replication |
| SA1721 | *pcrA* | ATP-depentend DNA helicase | -2.1 | 0.13 | DNA replication |
| SA1513 | *polA* | DNA polymerase I | 2.4 | 0.13 | DNA replication |
| SA1710 |  | Hypothetical protein, similar to DNA polymerase III, alpha chain PolC type | 2.9 | 0.06 | DNA replication |
| SA1286 |  | Hypothetical protein, similar to chromosome replication initiation protein dnaD | 3.0 | 0.00 | DNA replication |
| SA0964 |  | Hypothetical protein, similar to heme synthase | -2.4 | 0.07 | [Membrane bioenergetics (electron transport chain and ATP synthase)](http://www.bio.nite.go.jp/dogan/GeneSearchResult?GENE_LIST_TYPE=1&type=504&GENOME_LIST=n315G1&CLASS_ID=31.04&WITH_GENE_MAP=1) |
| SA0965 | *ctaB* | Cytochrome caa3 oxidase (assembly factor) homolog | -2.3 | 0.07 | [Membrane bioenergetics (electron transport chain and ATP synthase)](http://www.bio.nite.go.jp/dogan/GeneSearchResult?GENE_LIST_TYPE=1&type=504&GENOME_LIST=n315G1&CLASS_ID=31.04&WITH_GENE_MAP=1) |
| SA1241 |  | Hypothetical protein, similar to nitric-oxide reductase | -2.3 | 0.13 | [Membrane bioenergetics (electron transport chain and ATP synthase)](http://www.bio.nite.go.jp/dogan/GeneSearchResult?GENE_LIST_TYPE=1&type=504&GENOME_LIST=n315G1&CLASS_ID=31.04&WITH_GENE_MAP=1) |
| SA0719 | *trxB* | Thioredoxine reductase | 2.0 | 0.38 | [Membrane bioenergetics (electron transport chain and ATP synthase)](http://www.bio.nite.go.jp/dogan/GeneSearchResult?GENE_LIST_TYPE=1&type=504&GENOME_LIST=n315G1&CLASS_ID=31.04&WITH_GENE_MAP=1) |
| SA1565 |  | Thioredoxin homolog | 2.2 | 0.20 | [Membrane bioenergetics (electron transport chain and ATP synthase)](http://www.bio.nite.go.jp/dogan/GeneSearchResult?GENE_LIST_TYPE=1&type=504&GENOME_LIST=n315G1&CLASS_ID=31.04&WITH_GENE_MAP=1) |
| SA0367 |  | Hypothetical protein, similar to nitro/flavin reductase | 2.8 | 0.06 | [Membrane bioenergetics (electron transport chain and ATP synthase)](http://www.bio.nite.go.jp/dogan/GeneSearchResult?GENE_LIST_TYPE=1&type=504&GENOME_LIST=n315G1&CLASS_ID=31.04&WITH_GENE_MAP=1) |
| SA0411 | *ndhF* | NADH dehydrogenase subunit 5 | 2.9 | 0.06 | [Membrane bioenergetics (electron transport chain and ATP synthase)](http://www.bio.nite.go.jp/dogan/GeneSearchResult?GENE_LIST_TYPE=1&type=504&GENOME_LIST=n315G1&CLASS_ID=31.04&WITH_GENE_MAP=1) |
| SA0992 | *trxA* | Thioredoxin | 3.0 | 0.00 | [Membrane bioenergetics (electron transport chain and ATP synthase)](http://www.bio.nite.go.jp/dogan/GeneSearchResult?GENE_LIST_TYPE=1&type=504&GENOME_LIST=n315G1&CLASS_ID=31.04&WITH_GENE_MAP=1) |
| SA0758 |  | Hypothetical protein, similar to thioredoxin | 3.2 | 0.00 | [Membrane bioenergetics (electron transport chain and ATP synthase)](http://www.bio.nite.go.jp/dogan/GeneSearchResult?GENE_LIST_TYPE=1&type=504&GENOME_LIST=n315G1&CLASS_ID=31.04&WITH_GENE_MAP=1) |
| SA1989 |  | Hypothetical protein, similar to imilar oxidoreductase | 5.4 | 0.00 | [Membrane bioenergetics (electron transport chain and ATP synthase)](http://www.bio.nite.go.jp/dogan/GeneSearchResult?GENE_LIST_TYPE=1&type=504&GENOME_LIST=n315G1&CLASS_ID=31.04&WITH_GENE_MAP=1) |
| SA0817 |  | Hypothetical protein, similar to NADH-dependent flavin oxidoreductase | 5.9 | 0.00 | [Membrane bioenergetics (electron transport chain and ATP synthase)](http://www.bio.nite.go.jp/dogan/GeneSearchResult?GENE_LIST_TYPE=1&type=504&GENOME_LIST=n315G1&CLASS_ID=31.04&WITH_GENE_MAP=1) |
| SA2162 |  | Hypothetical protein, similar to thioredoxin reductase | 7.0 | 0.00 | [Membrane bioenergetics (electron transport chain and ATP synthase)](http://www.bio.nite.go.jp/dogan/GeneSearchResult?GENE_LIST_TYPE=1&type=504&GENOME_LIST=n315G1&CLASS_ID=31.04&WITH_GENE_MAP=1) |
| SA2324 |  | Hypothetical protein, similar to thioredoxin | 7.1 | 0.00 | [Membrane bioenergetics (electron transport chain and ATP synthase)](http://www.bio.nite.go.jp/dogan/GeneSearchResult?GENE_LIST_TYPE=1&type=504&GENOME_LIST=n315G1&CLASS_ID=31.04&WITH_GENE_MAP=1) |
| SA0008 | *hutH* | Histidine ammonia-lyase | -14.6 | 0.00 | [Metabolism of amino acids and related molecules](http://www.bio.nite.go.jp/dogan/GeneSearchResult?GENE_LIST_TYPE=1&type=504&GENOME_LIST=n315G1&CLASS_ID=32.02&WITH_GENE_MAP=1) |
| SA0419 | *metB* | Cystathionine gamma-synthase | -11.1 | 0.00 | [Metabolism of amino acids and related molecules](http://www.bio.nite.go.jp/dogan/GeneSearchResult?GENE_LIST_TYPE=1&type=504&GENOME_LIST=n315G1&CLASS_ID=32.02&WITH_GENE_MAP=1) |
| SA0418 | *cysM* | Cysteine synthase homologue | -7.8 | 0.00 | [Metabolism of amino acids and related molecules](http://www.bio.nite.go.jp/dogan/GeneSearchResult?GENE_LIST_TYPE=1&type=504&GENOME_LIST=n315G1&CLASS_ID=32.02&WITH_GENE_MAP=1) |
| SA1012 | *argF* | Ornithine carbamoyltransferase | -6.4 | 0.00 | [Metabolism of amino acids and related molecules](http://www.bio.nite.go.jp/dogan/GeneSearchResult?GENE_LIST_TYPE=1&type=504&GENOME_LIST=n315G1&CLASS_ID=32.02&WITH_GENE_MAP=1) |
| SA1298 | *aroB* | 3-dehydroquinate synthase | -3.6 | 0.00 | [Metabolism of amino acids and related molecules](http://www.bio.nite.go.jp/dogan/GeneSearchResult?GENE_LIST_TYPE=1&type=504&GENOME_LIST=n315G1&CLASS_ID=32.02&WITH_GENE_MAP=1) |
| SA1297 | *aroA* | 3-phosphoshikimate 1-carboxyvinyltransferase | -3.6 | 0.00 | [Metabolism of amino acids and related molecules](http://www.bio.nite.go.jp/dogan/GeneSearchResult?GENE_LIST_TYPE=1&type=504&GENOME_LIST=n315G1&CLASS_ID=32.02&WITH_GENE_MAP=1) |
| SA2121 | *hutI* | Imidazolonepropionase | -3.5 | 0.00 | [Metabolism of amino acids and related molecules](http://www.bio.nite.go.jp/dogan/GeneSearchResult?GENE_LIST_TYPE=1&type=504&GENOME_LIST=n315G1&CLASS_ID=32.02&WITH_GENE_MAP=1) |
| SA1310 | *ansA* | Probable L-asparaginase | -3.1 | 0.00 | [Metabolism of amino acids and related molecules](http://www.bio.nite.go.jp/dogan/GeneSearchResult?GENE_LIST_TYPE=1&type=504&GENOME_LIST=n315G1&CLASS_ID=32.02&WITH_GENE_MAP=1) |
| SA1608 | *metK* | S-adenosylmethionine synthetase | -2.8 | 0.00 | [Metabolism of amino acids and related molecules](http://www.bio.nite.go.jp/dogan/GeneSearchResult?GENE_LIST_TYPE=1&type=504&GENOME_LIST=n315G1&CLASS_ID=32.02&WITH_GENE_MAP=1) |
| SA1424 | *aroE* | Shikimate dehydrogenease | -2.8 | 0.00 | [Metabolism of amino acids and related molecules](http://www.bio.nite.go.jp/dogan/GeneSearchResult?GENE_LIST_TYPE=1&type=504&GENOME_LIST=n315G1&CLASS_ID=32.02&WITH_GENE_MAP=1) |
| SA2095 |  | Hypothetical protein, similar to D-octopine dehydrogenase | -2.8 | 0.00 | [Metabolism of amino acids and related molecules](http://www.bio.nite.go.jp/dogan/GeneSearchResult?GENE_LIST_TYPE=1&type=504&GENOME_LIST=n315G1&CLASS_ID=32.02&WITH_GENE_MAP=1) |
| SA1423 |  | Conserved hypothetical protein | -2.7 | 0.00 | [Metabolism of amino acids and related molecules](http://www.bio.nite.go.jp/dogan/GeneSearchResult?GENE_LIST_TYPE=1&type=504&GENOME_LIST=n315G1&CLASS_ID=32.02&WITH_GENE_MAP=1) |
| SA0169 |  | Hypothetical protein, similar to acyl-CoA dehydrogenase family protein | -2.5 | 0.07 | [Metabolism of amino acids and related molecules](http://www.bio.nite.go.jp/dogan/GeneSearchResult?GENE_LIST_TYPE=1&type=504&GENOME_LIST=n315G1&CLASS_ID=32.02&WITH_GENE_MAP=1) |
| SA2099 |  | Hypothetical protein, similar to monooxygenase | -2.4 | 0.07 | [Metabolism of amino acids and related molecules](http://www.bio.nite.go.jp/dogan/GeneSearchResult?GENE_LIST_TYPE=1&type=504&GENOME_LIST=n315G1&CLASS_ID=32.02&WITH_GENE_MAP=1) |
| SA1121 |  | Hypothetical protein, similar to processing proteinase homolog | -2.3 | 0.13 | [Metabolism of amino acids and related molecules](http://www.bio.nite.go.jp/dogan/GeneSearchResult?GENE_LIST_TYPE=1&type=504&GENOME_LIST=n315G1&CLASS_ID=32.02&WITH_GENE_MAP=1) |
| SA2189 |  | Hypothetical protein, 4imilar to NirR | -2.2 | 0.13 | [Metabolism of amino acids and related molecules](http://www.bio.nite.go.jp/dogan/GeneSearchResult?GENE_LIST_TYPE=1&type=504&GENOME_LIST=n315G1&CLASS_ID=32.02&WITH_GENE_MAP=1) |
| SA1271 |  | Threonine deaminase IlvA homolog | -2.1 | 0.13 | [Metabolism of amino acids and related molecules](http://www.bio.nite.go.jp/dogan/GeneSearchResult?GENE_LIST_TYPE=1&type=504&GENOME_LIST=n315G1&CLASS_ID=32.02&WITH_GENE_MAP=1) |
| SA0011 |  | Hypothetical protein, similar to homoserine-o-acetyltransferase | 2.1 | 0.38 | [Metabolism of amino acids and related molecules](http://www.bio.nite.go.jp/dogan/GeneSearchResult?GENE_LIST_TYPE=1&type=504&GENOME_LIST=n315G1&CLASS_ID=32.02&WITH_GENE_MAP=1) |
| SA2088 | *ureD* | urease accessory protein UreD | 2.1 | 0.38 | [Metabolism of amino acids and related molecules](http://www.bio.nite.go.jp/dogan/GeneSearchResult?GENE_LIST_TYPE=1&type=504&GENOME_LIST=n315G1&CLASS_ID=32.02&WITH_GENE_MAP=1) |
| SA1199 |  | Hypothetical protein, similar to anthranilate synthase component I | 2.5 | 0.20 | [Metabolism of amino acids and related molecules](http://www.bio.nite.go.jp/dogan/GeneSearchResult?GENE_LIST_TYPE=1&type=504&GENOME_LIST=n315G1&CLASS_ID=32.02&WITH_GENE_MAP=1) |
| SA0313 |  | Hypothetical protein, similar to glycine cleavage system H protein | 2.7 | 0.06 | [Metabolism of amino acids and related molecules](http://www.bio.nite.go.jp/dogan/GeneSearchResult?GENE_LIST_TYPE=1&type=504&GENOME_LIST=n315G1&CLASS_ID=32.02&WITH_GENE_MAP=1) |
| SA1343 |  | Hypothetical protein, similar to tripeptidase | 2.8 | 0.00 | [Metabolism of amino acids and related molecules](http://www.bio.nite.go.jp/dogan/GeneSearchResult?GENE_LIST_TYPE=1&type=504&GENOME_LIST=n315G1&CLASS_ID=32.02&WITH_GENE_MAP=1) |
| SA2397 |  | Hypothetical protein, similar to pyridoxal-phosphate dependent aminotransferase | 2.9 | 0.00 | [Metabolism of amino acids and related molecules](http://www.bio.nite.go.jp/dogan/GeneSearchResult?GENE_LIST_TYPE=1&type=504&GENOME_LIST=n315G1&CLASS_ID=32.02&WITH_GENE_MAP=1) |
| SA0507 |  | Hypothetical protein, similar to N-acyl-L-amino acid amidohydrolase | 2.9 | 0.00 | [Metabolism of amino acids and related molecules](http://www.bio.nite.go.jp/dogan/GeneSearchResult?GENE_LIST_TYPE=1&type=504&GENOME_LIST=n315G1&CLASS_ID=32.02&WITH_GENE_MAP=1) |
| SA1858 | *ilvD* | Dihydroxy-acid dehydratase | 2.9 | 0.00 | [Metabolism of amino acids and related molecules](http://www.bio.nite.go.jp/dogan/GeneSearchResult?GENE_LIST_TYPE=1&type=504&GENOME_LIST=n315G1&CLASS_ID=32.02&WITH_GENE_MAP=1) |
| SA1545 | *serA* | D-3-phosphoglycerate dehydrogenase | 3.0 | 0.00 | [Metabolism of amino acids and related molecules](http://www.bio.nite.go.jp/dogan/GeneSearchResult?GENE_LIST_TYPE=1&type=504&GENOME_LIST=n315G1&CLASS_ID=32.02&WITH_GENE_MAP=1) |
| SA1861 | *ilvC* | Alpha-keto-beta-hydroxylacil reductoisomerase | 5.3 | 0.00 | [Metabolism of amino acids and related molecules](http://www.bio.nite.go.jp/dogan/GeneSearchResult?GENE_LIST_TYPE=1&type=504&GENOME_LIST=n315G1&CLASS_ID=32.02&WITH_GENE_MAP=1) |
| SA1165 | *thrC* | Threonine synthase | 6.3 | 0.00 | [Metabolism of amino acids and related molecules](http://www.bio.nite.go.jp/dogan/GeneSearchResult?GENE_LIST_TYPE=1&type=504&GENOME_LIST=n315G1&CLASS_ID=32.02&WITH_GENE_MAP=1) |
| SA1862 | *leuA* | 2-isopropylmalate synthase | 6.6 | 0.00 | [Metabolism of amino acids and related molecules](http://www.bio.nite.go.jp/dogan/GeneSearchResult?GENE_LIST_TYPE=1&type=504&GENOME_LIST=n315G1&CLASS_ID=32.02&WITH_GENE_MAP=1) |
| SA1205 | *trpA* | Tryptophan synthase alpha chain | 6.7 | 0.00 | [Metabolism of amino acids and related molecules](http://www.bio.nite.go.jp/dogan/GeneSearchResult?GENE_LIST_TYPE=1&type=504&GENOME_LIST=n315G1&CLASS_ID=32.02&WITH_GENE_MAP=1) |
| SA1859 | *ilvB* | Acetolactate synthase large subunit | 7.0 | 0.00 | [Metabolism of amino acids and related molecules](http://www.bio.nite.go.jp/dogan/GeneSearchResult?GENE_LIST_TYPE=1&type=504&GENOME_LIST=n315G1&CLASS_ID=32.02&WITH_GENE_MAP=1) |
| SA1860 |  | Hypothetical protein, similar to acetolactate synthase small subunit | 8.2 | 0.00 | [Metabolism of amino acids and related molecules](http://www.bio.nite.go.jp/dogan/GeneSearchResult?GENE_LIST_TYPE=1&type=504&GENOME_LIST=n315G1&CLASS_ID=32.02&WITH_GENE_MAP=1) |
| SA1203 | *trpF* | Phosphoriborylanthranilate isomerase | 9.6 | 0.00 | [Metabolism of amino acids and related molecules](http://www.bio.nite.go.jp/dogan/GeneSearchResult?GENE_LIST_TYPE=1&type=504&GENOME_LIST=n315G1&CLASS_ID=32.02&WITH_GENE_MAP=1) |
| SA1202 | *trpC* | Indole-3-glycerol phosphate synthase | 10.3 | 0.00 | [Metabolism of amino acids and related molecules](http://www.bio.nite.go.jp/dogan/GeneSearchResult?GENE_LIST_TYPE=1&type=504&GENOME_LIST=n315G1&CLASS_ID=32.02&WITH_GENE_MAP=1) |
| SA1204 | *trpB* | Tryptophan synthase beta chain | 11.8 | 0.00 | [Metabolism of amino acids and related molecules](http://www.bio.nite.go.jp/dogan/GeneSearchResult?GENE_LIST_TYPE=1&type=504&GENOME_LIST=n315G1&CLASS_ID=32.02&WITH_GENE_MAP=1) |
| SA0419 | *metB* | Cystathionine gamma-synthase | -11.0 | 0.00 | [Metabolism of amino acids and related molecules](http://www.bio.nite.go.jp/dogan/GeneSearchResult?GENE_LIST_TYPE=1&type=504&GENOME_LIST=n315G1&CLASS_ID=32.02&WITH_GENE_MAP=1) |
| SA1272 |  | Alanine dehydrogenase | -5.0 | 0.13 | [Metabolism of amino acids and related molecules](http://www.bio.nite.go.jp/dogan/GeneSearchResult?GENE_LIST_TYPE=1&type=504&GENOME_LIST=n315G1&CLASS_ID=32.02&WITH_GENE_MAP=1) |
| SA2122 | *hutU* | Urocanate hydratase | -4.5 | 0.00 | [Metabolism of amino acids and related molecules](http://www.bio.nite.go.jp/dogan/GeneSearchResult?GENE_LIST_TYPE=1&type=504&GENOME_LIST=n315G1&CLASS_ID=32.02&WITH_GENE_MAP=1) |
| SA0822 | *argG* | Argininosuccinate synthase | -2.7 | 0.00 | [Metabolism of amino acids and related molecules](http://www.bio.nite.go.jp/dogan/GeneSearchResult?GENE_LIST_TYPE=1&type=504&GENOME_LIST=n315G1&CLASS_ID=32.02&WITH_GENE_MAP=1) |
| SA1122 |  | Hypothetical protein, similar to precessing proteinase | -2.3 | 0.07 | [Metabolism of amino acids and related molecules](http://www.bio.nite.go.jp/dogan/GeneSearchResult?GENE_LIST_TYPE=1&type=504&GENOME_LIST=n315G1&CLASS_ID=32.02&WITH_GENE_MAP=1) |
| SA0098 |  | Hypothetical protein, similar to aminoacylase | 2.2 | 0.20 | [Metabolism of amino acids and related molecules](http://www.bio.nite.go.jp/dogan/GeneSearchResult?GENE_LIST_TYPE=1&type=504&GENOME_LIST=n315G1&CLASS_ID=32.02&WITH_GENE_MAP=1) |
| SA1864 | *leuC* | 3-Isopropylmalate dehydratase large subunit | 2.8 | 0.06 | [Metabolism of amino acids and related molecules](http://www.bio.nite.go.jp/dogan/GeneSearchResult?GENE_LIST_TYPE=1&type=504&GENOME_LIST=n315G1&CLASS_ID=32.02&WITH_GENE_MAP=1) |
| SA1865 | *leuD* | 3-Isopropylmalate dehydratase small subunit | 2.9 | 0.06 | [Metabolism of amino acids and related molecules](http://www.bio.nite.go.jp/dogan/GeneSearchResult?GENE_LIST_TYPE=1&type=504&GENOME_LIST=n315G1&CLASS_ID=32.02&WITH_GENE_MAP=1) |
| SA1163 |  | Aspartate kinase homolog | 4.8 | 0.00 | [Metabolism of amino acids and related molecules](http://www.bio.nite.go.jp/dogan/GeneSearchResult?GENE_LIST_TYPE=1&type=504&GENOME_LIST=n315G1&CLASS_ID=32.02&WITH_GENE_MAP=1) |
| SA1863 | *leuB* | 3-Isopropylmalate dehydrogenase | 4.8 | 0.00 | [Metabolism of amino acids and related molecules](http://www.bio.nite.go.jp/dogan/GeneSearchResult?GENE_LIST_TYPE=1&type=504&GENOME_LIST=n315G1&CLASS_ID=32.02&WITH_GENE_MAP=1) |
| SA2347 |  | Hypothetical protein, similar to aspartate aminotransferase | 5.0 | 0.00 | [Metabolism of amino acids and related molecules](http://www.bio.nite.go.jp/dogan/GeneSearchResult?GENE_LIST_TYPE=1&type=504&GENOME_LIST=n315G1&CLASS_ID=32.02&WITH_GENE_MAP=1) |
| SA1164 | *dhoM* | Homoserine dehydrogenase | 5.7 | 0.00 | [Metabolism of amino acids and related molecules](http://www.bio.nite.go.jp/dogan/GeneSearchResult?GENE_LIST_TYPE=1&type=504&GENOME_LIST=n315G1&CLASS_ID=32.02&WITH_GENE_MAP=1) |
| SA1166 | *thrB* | Homoserine kinase homolog | 7.3 | 0.00 | [Metabolism of amino acids and related molecules](http://www.bio.nite.go.jp/dogan/GeneSearchResult?GENE_LIST_TYPE=1&type=504&GENOME_LIST=n315G1&CLASS_ID=32.02&WITH_GENE_MAP=1) |
| SA0304 | *nanA* | N-acetylneuraminate lyase subunit | -5.5 | 0.00 | [Metabolism of carbohydrates and related molecules](http://www.bio.nite.go.jp/dogan/GeneSearchResult?GENE_LIST_TYPE=1&type=504&GENOME_LIST=n315G1&CLASS_ID=32.01&WITH_GENE_MAP=1) |
| SA0232 | *lctE* | L-lactate dehydrogenase | -4.0 | 0.00 | [Metabolism of carbohydrates and related molecules](http://www.bio.nite.go.jp/dogan/GeneSearchResult?GENE_LIST_TYPE=1&type=504&GENOME_LIST=n315G1&CLASS_ID=32.01&WITH_GENE_MAP=1) |
| SA1945 |  | Hypothetical protein, similar to imilar-6 phospate isomelase pmi | -2.8 | 0.00 | [Metabolism of carbohydrates and related molecules](http://www.bio.nite.go.jp/dogan/GeneSearchResult?GENE_LIST_TYPE=1&type=504&GENOME_LIST=n315G1&CLASS_ID=32.01&WITH_GENE_MAP=1) |
| SA0299 |  | Hypothetical protein, similar to carbohydrate kinase, PfkB family | -2.6 | 0.00 | [Metabolism of carbohydrates and related molecules](http://www.bio.nite.go.jp/dogan/GeneSearchResult?GENE_LIST_TYPE=1&type=504&GENOME_LIST=n315G1&CLASS_ID=32.01&WITH_GENE_MAP=1) |
| SA1945 |  | Hypothetical protein, similar to imilar-6 phospate isomelase pmi | -2.6 | 0.07 | [Metabolism of carbohydrates and related molecules](http://www.bio.nite.go.jp/dogan/GeneSearchResult?GENE_LIST_TYPE=1&type=504&GENOME_LIST=n315G1&CLASS_ID=32.01&WITH_GENE_MAP=1) |
| SA2007 |  | Hypothetical protein, similar to alpha-acetolactate decarboxylase | -2.4 | 0.07 | [Metabolism of carbohydrates and related molecules](http://www.bio.nite.go.jp/dogan/GeneSearchResult?GENE_LIST_TYPE=1&type=504&GENOME_LIST=n315G1&CLASS_ID=32.01&WITH_GENE_MAP=1) |
| SA1724 | *purB* | Adenylosuccinate lyase | -2.2 | 0.11 | [Metabolism of carbohydrates and related molecules](http://www.bio.nite.go.jp/dogan/GeneSearchResult?GENE_LIST_TYPE=1&type=504&GENOME_LIST=n315G1&CLASS_ID=32.01&WITH_GENE_MAP=1) |
| SA0790 |  | Hypothetical protein, similar to N-acetyl-glucosamine catabolism homologue | -2.1 | 0.19 | [Metabolism of carbohydrates and related molecules](http://www.bio.nite.go.jp/dogan/GeneSearchResult?GENE_LIST_TYPE=1&type=504&GENOME_LIST=n315G1&CLASS_ID=32.01&WITH_GENE_MAP=1) |
| SA0433 |  | Alpha-glucosidase | -2.1 | 0.11 | [Metabolism of carbohydrates and related molecules](http://www.bio.nite.go.jp/dogan/GeneSearchResult?GENE_LIST_TYPE=1&type=504&GENOME_LIST=n315G1&CLASS_ID=32.01&WITH_GENE_MAP=1) |
| SA2399 |  | Fructose-bisphosphate aldolase homolog | 2.1 | 0.25 | [Metabolism of carbohydrates and related molecules](http://www.bio.nite.go.jp/dogan/GeneSearchResult?GENE_LIST_TYPE=1&type=504&GENOME_LIST=n315G1&CLASS_ID=32.01&WITH_GENE_MAP=1) |
| SA1342 | *gnd* | Phosphogluconate dehydrogenase (decarboxylating) | 2.1 | 0.25 | [Metabolism of carbohydrates and related molecules](http://www.bio.nite.go.jp/dogan/GeneSearchResult?GENE_LIST_TYPE=1&type=504&GENOME_LIST=n315G1&CLASS_ID=32.01&WITH_GENE_MAP=1) |
| SA0219 | *pflA* | Formate acetyltransferase activating enzyme | 2.2 | 0.20 | [Metabolism of carbohydrates and related molecules](http://www.bio.nite.go.jp/dogan/GeneSearchResult?GENE_LIST_TYPE=1&type=504&GENOME_LIST=n315G1&CLASS_ID=32.01&WITH_GENE_MAP=1) |
| SA2327 | *cidC* | Hypothetical protein, similar to pyruvate oxidase | 2.4 | 0.38 | [Metabolism of carbohydrates and related molecules](http://www.bio.nite.go.jp/dogan/GeneSearchResult?GENE_LIST_TYPE=1&type=504&GENOME_LIST=n315G1&CLASS_ID=32.01&WITH_GENE_MAP=1) |
| SA0943 | *pdhA* | Pyrubate dehydrogenase E1 component alpha subunit | 2.3 | 0.20 | [Metabolism of carbohydrates and related molecules](http://www.bio.nite.go.jp/dogan/GeneSearchResult?GENE_LIST_TYPE=1&type=504&GENOME_LIST=n315G1&CLASS_ID=32.01&WITH_GENE_MAP=1) |
| SA1599 |  | Hypothetical protein, similar to transaldolase | 2.4 | 0.13 | [Metabolism of carbohydrates and related molecules](http://www.bio.nite.go.jp/dogan/GeneSearchResult?GENE_LIST_TYPE=1&type=504&GENOME_LIST=n315G1&CLASS_ID=32.01&WITH_GENE_MAP=1) |
| SA1566 |  | Endo-1,4-beta-glucanase homolog | 2.4 | 0.13 | [Metabolism of carbohydrates and related molecules](http://www.bio.nite.go.jp/dogan/GeneSearchResult?GENE_LIST_TYPE=1&type=504&GENOME_LIST=n315G1&CLASS_ID=32.01&WITH_GENE_MAP=1) |
| SA1177 | *tkt* | Transketolase | 2.6 | 0.06 | [Metabolism of carbohydrates and related molecules](http://www.bio.nite.go.jp/dogan/GeneSearchResult?GENE_LIST_TYPE=1&type=504&GENOME_LIST=n315G1&CLASS_ID=32.01&WITH_GENE_MAP=1) |
| SA1988 |  | Hypothetical protein, similar to alginate lyase | 2.6 | 0.06 | [Metabolism of carbohydrates and related molecules](http://www.bio.nite.go.jp/dogan/GeneSearchResult?GENE_LIST_TYPE=1&type=504&GENOME_LIST=n315G1&CLASS_ID=32.01&WITH_GENE_MAP=1) |
| SA0697 |  | Hypothetical protein, similar to glycerate kinase | 2.8 | 0.00 | [Metabolism of carbohydrates and related molecules](http://www.bio.nite.go.jp/dogan/GeneSearchResult?GENE_LIST_TYPE=1&type=504&GENOME_LIST=n315G1&CLASS_ID=32.01&WITH_GENE_MAP=1) |
| SA2244 |  | Hypothetical protein, similar to endo-1,4-beta-glucanase | 3.1 | 0.00 | [Metabolism of carbohydrates and related molecules](http://www.bio.nite.go.jp/dogan/GeneSearchResult?GENE_LIST_TYPE=1&type=504&GENOME_LIST=n315G1&CLASS_ID=32.01&WITH_GENE_MAP=1) |
| SA2075 | *narQ* | FdhD protein homolog | 4.2 | 0.00 | [Metabolism of carbohydrates and related molecules](http://www.bio.nite.go.jp/dogan/GeneSearchResult?GENE_LIST_TYPE=1&type=504&GENOME_LIST=n315G1&CLASS_ID=32.01&WITH_GENE_MAP=1) |
| SA2346 |  | Hypothetical protein, similar to D-specific D-2-hydroxyacid dehydrogenase ddh homolog | 4.3 | 0.00 | [Metabolism of carbohydrates and related molecules](http://www.bio.nite.go.jp/dogan/GeneSearchResult?GENE_LIST_TYPE=1&type=504&GENOME_LIST=n315G1&CLASS_ID=32.01&WITH_GENE_MAP=1) |
| SA2008 | *alsS* | Alpha-acetolactate synthase | -3.4 | 0.00 | [Metabolism of carbohydrates and related molecules](http://www.bio.nite.go.jp/dogan/GeneSearchResult?GENE_LIST_TYPE=1&type=504&GENOME_LIST=n315G1&CLASS_ID=32.01&WITH_GENE_MAP=1) |
| SA2312 | *ddh* | D-specific D-2-hydroxyacid dehydrogenase | -3.4 | 0.00 | [Metabolism of carbohydrates and related molecules](http://www.bio.nite.go.jp/dogan/GeneSearchResult?GENE_LIST_TYPE=1&type=504&GENOME_LIST=n315G1&CLASS_ID=32.01&WITH_GENE_MAP=1) |
| SAS020 |  | Hypothetical protein, similar to phosphoglycerate mutase | -2.3 | 0.11 | [Metabolism of carbohydrates and related molecules](http://www.bio.nite.go.jp/dogan/GeneSearchResult?GENE_LIST_TYPE=1&type=504&GENOME_LIST=n315G1&CLASS_ID=32.01&WITH_GENE_MAP=1) |
| SA2231 |  | Hypothetical protein, similar to glucose epimerase | 2.2 | 0.25 | [Metabolism of carbohydrates and related molecules](http://www.bio.nite.go.jp/dogan/GeneSearchResult?GENE_LIST_TYPE=1&type=504&GENOME_LIST=n315G1&CLASS_ID=32.01&WITH_GENE_MAP=1) |
| SA2304 | *fbp* | Fructose-bisphosphatase | 2.3 | 0.19 | [Metabolism of carbohydrates and related molecules](http://www.bio.nite.go.jp/dogan/GeneSearchResult?GENE_LIST_TYPE=1&type=504&GENOME_LIST=n315G1&CLASS_ID=32.01&WITH_GENE_MAP=1) |
| SA2402 |  | Acetate-CoA ligase (EC 6.2.1.1) | 2.5 | 0.06 | [Metabolism of carbohydrates and related molecules](http://www.bio.nite.go.jp/dogan/GeneSearchResult?GENE_LIST_TYPE=1&type=504&GENOME_LIST=n315G1&CLASS_ID=32.01&WITH_GENE_MAP=1) |
| SA0958 |  | Myo-inositol-1(or 4)-monophosphatase homolog | 2.6 | 0.11 | [Metabolism of carbohydrates and related molecules](http://www.bio.nite.go.jp/dogan/GeneSearchResult?GENE_LIST_TYPE=1&type=504&GENOME_LIST=n315G1&CLASS_ID=32.01&WITH_GENE_MAP=1) |
| SA0527 | *nagB* | Probable glucosamine-6-phosphate isomerase | 2.9 | 0.00 | [Metabolism of carbohydrates and related molecules](http://www.bio.nite.go.jp/dogan/GeneSearchResult?GENE_LIST_TYPE=1&type=504&GENOME_LIST=n315G1&CLASS_ID=32.01&WITH_GENE_MAP=1) |
| SA2294 | *gntK* | Gluconokinase | 5.7 | 0.00 | [Metabolism of carbohydrates and related molecules](http://www.bio.nite.go.jp/dogan/GeneSearchResult?GENE_LIST_TYPE=1&type=504&GENOME_LIST=n315G1&CLASS_ID=32.01&WITH_GENE_MAP=1) |
| SA0122 | *butA* | Acetoin(diacetyl)reductase | 11.8 | 0.00 | [Metabolism of carbohydrates and related molecules](http://www.bio.nite.go.jp/dogan/GeneSearchResult?GENE_LIST_TYPE=1&type=504&GENOME_LIST=n315G1&CLASS_ID=32.01&WITH_GENE_MAP=1) |
| SA2077 |  | Hypothetical protein, similar to biotin biosynthesis protein | -4.3 | 0.00 | [Metabolism of coenzymes and prosthetic groups](http://www.bio.nite.go.jp/dogan/GeneSearchResult?GENE_LIST_TYPE=1&type=504&GENOME_LIST=n315G1&CLASS_ID=32.05&WITH_GENE_MAP=1) |
| SA1588 | *ribB* | Riboflavin synthase alpha chain | -3.6 | 0.00 | [Metabolism of coenzymes and prosthetic groups](http://www.bio.nite.go.jp/dogan/GeneSearchResult?GENE_LIST_TYPE=1&type=504&GENOME_LIST=n315G1&CLASS_ID=32.05&WITH_GENE_MAP=1) |
| SA1587 | *ribA* | Riboflavin biosynthesis protein | -3.5 | 0.00 | [Metabolism of coenzymes and prosthetic groups](http://www.bio.nite.go.jp/dogan/GeneSearchResult?GENE_LIST_TYPE=1&type=504&GENOME_LIST=n315G1&CLASS_ID=32.05&WITH_GENE_MAP=1) |
| SA0473 | *folB* | 7,8-Dihydroneopterin aldolase | -3.3 | 0.00 | [Metabolism of coenzymes and prosthetic groups](http://www.bio.nite.go.jp/dogan/GeneSearchResult?GENE_LIST_TYPE=1&type=504&GENOME_LIST=n315G1&CLASS_ID=32.05&WITH_GENE_MAP=1) |
| SA0474 | *folK* | 2-Amino-4-hydroxy-6-hydroxymethyldihydropteridine pyrophosphokinase | -3.3 | 0.00 | [Metabolism of coenzymes and prosthetic groups](http://www.bio.nite.go.jp/dogan/GeneSearchResult?GENE_LIST_TYPE=1&type=504&GENOME_LIST=n315G1&CLASS_ID=32.05&WITH_GENE_MAP=1) |
| SA0472 | *folP* | Dihydropteroate synthase chain A synthetase | -2.8 | 0.00 | [Metabolism of coenzymes and prosthetic groups](http://www.bio.nite.go.jp/dogan/GeneSearchResult?GENE_LIST_TYPE=1&type=504&GENOME_LIST=n315G1&CLASS_ID=32.05&WITH_GENE_MAP=1) |
| SA1586 | *ribH* | 6,7-Dimethyl-8-ribityllumazine synthase | -2.6 | 0.07 | [Metabolism of coenzymes and prosthetic groups](http://www.bio.nite.go.jp/dogan/GeneSearchResult?GENE_LIST_TYPE=1&type=504&GENOME_LIST=n315G1&CLASS_ID=32.05&WITH_GENE_MAP=1) |
| SA0894 |  | Hypothetical protein, similar to 1,4-dihydroxy-2-naphthodate octaprenyltransferase | -2.3 | 0.13 | [Metabolism of coenzymes and prosthetic groups](http://www.bio.nite.go.jp/dogan/GeneSearchResult?GENE_LIST_TYPE=1&type=504&GENOME_LIST=n315G1&CLASS_ID=32.05&WITH_GENE_MAP=1) |
| SA2214 | *bioA* | Adenosylmethionine-8-amino-7-oxononanoate aminotransferase | -2.2 | 0.11 | [Metabolism of coenzymes and prosthetic groups](http://www.bio.nite.go.jp/dogan/GeneSearchResult?GENE_LIST_TYPE=1&type=504&GENOME_LIST=n315G1&CLASS_ID=32.05&WITH_GENE_MAP=1) |
| SA1493 | *hemD* | Uroporphyrinogen III synthase | -2.2 | 0.11 | [Metabolism of coenzymes and prosthetic groups](http://www.bio.nite.go.jp/dogan/GeneSearchResult?GENE_LIST_TYPE=1&type=504&GENOME_LIST=n315G1&CLASS_ID=32.05&WITH_GENE_MAP=1) |
| SA1494 | *hemC* | Porphobilinogen deaminase | -2.2 | 0.13 | [Metabolism of coenzymes and prosthetic groups](http://www.bio.nite.go.jp/dogan/GeneSearchResult?GENE_LIST_TYPE=1&type=504&GENOME_LIST=n315G1&CLASS_ID=32.05&WITH_GENE_MAP=1) |
| SA1495 | *hemX* | HemA concentration negative effector hemX | -2.0 | 0.20 | [Metabolism of coenzymes and prosthetic groups](http://www.bio.nite.go.jp/dogan/GeneSearchResult?GENE_LIST_TYPE=1&type=504&GENOME_LIST=n315G1&CLASS_ID=32.05&WITH_GENE_MAP=1) |
| SA0895 |  | Hypothetical protein, similar to menaquinone-specific isochorismate synthase | 2.1 | 0.38 | [Metabolism of coenzymes and prosthetic groups](http://www.bio.nite.go.jp/dogan/GeneSearchResult?GENE_LIST_TYPE=1&type=504&GENOME_LIST=n315G1&CLASS_ID=32.05&WITH_GENE_MAP=1) |
| SA2392 | *panB* | 3-Methyl-2-oxobutanoate hydroxymethyltransferase | 2.1 | 0.38 | [Metabolism of coenzymes and prosthetic groups](http://www.bio.nite.go.jp/dogan/GeneSearchResult?GENE_LIST_TYPE=1&type=504&GENOME_LIST=n315G1&CLASS_ID=32.05&WITH_GENE_MAP=1) |
| SA2065 | *moaD* | Probable molybdopterin synthase small subunit | 2.1 | 0.38 | [Metabolism of coenzymes and prosthetic groups](http://www.bio.nite.go.jp/dogan/GeneSearchResult?GENE_LIST_TYPE=1&type=504&GENOME_LIST=n315G1&CLASS_ID=32.05&WITH_GENE_MAP=1) |
| SA1115 | *ribC* | Riboflavin kinase / FAD synthase ribC | 2.1 | 0.38 | [Metabolism of coenzymes and prosthetic groups](http://www.bio.nite.go.jp/dogan/GeneSearchResult?GENE_LIST_TYPE=1&type=504&GENOME_LIST=n315G1&CLASS_ID=32.05&WITH_GENE_MAP=1) |
| SA2438 |  | Hypothetical protein, similar to N-Carbamoylsarcosine Amidohydrolase | 2.2 | 0.20 | [Metabolism of coenzymes and prosthetic groups](http://www.bio.nite.go.jp/dogan/GeneSearchResult?GENE_LIST_TYPE=1&type=504&GENOME_LIST=n315G1&CLASS_ID=32.05&WITH_GENE_MAP=1) |
| SA1054 |  | Pantothenate metabolism flavoprotein homolog | 2.3 | 0.19 | [Metabolism of coenzymes and prosthetic groups](http://www.bio.nite.go.jp/dogan/GeneSearchResult?GENE_LIST_TYPE=1&type=504&GENOME_LIST=n315G1&CLASS_ID=32.05&WITH_GENE_MAP=1) |
| SA2393 |  | Hypothetical protein, similar to 2-dehydropantoate 2-reductase | 2.9 | 0.00 | [Metabolism of coenzymes and prosthetic groups](http://www.bio.nite.go.jp/dogan/GeneSearchResult?GENE_LIST_TYPE=1&type=504&GENOME_LIST=n315G1&CLASS_ID=32.05&WITH_GENE_MAP=1) |
| SA0328 |  | Hypothetical protein, similar to NADH-dependent FMN reductase | 3.0 | 0.06 | [Metabolism of coenzymes and prosthetic groups](http://www.bio.nite.go.jp/dogan/GeneSearchResult?GENE_LIST_TYPE=1&type=504&GENOME_LIST=n315G1&CLASS_ID=32.05&WITH_GENE_MAP=1) |
| SA1589 | *ribD* | Riboflavin specific deaminase | -4.8 | 0.00 | [Metabolism of coenzymes and prosthetic groups](http://www.bio.nite.go.jp/dogan/GeneSearchResult?GENE_LIST_TYPE=1&type=504&GENOME_LIST=n315G1&CLASS_ID=32.05&WITH_GENE_MAP=1) |
| SA1919 |  | Hypothetical protein, 7imilar to protoporphyrinogen oxidase (hemK) | -4.1 | 0.00 | [Metabolism of coenzymes and prosthetic groups](http://www.bio.nite.go.jp/dogan/GeneSearchResult?GENE_LIST_TYPE=1&type=504&GENOME_LIST=n315G1&CLASS_ID=32.05&WITH_GENE_MAP=1) |
| SA1537 |  | Hypothetical protein, similar to thiamine biosynthesis protein ThiI | -3.0 | 0.00 | [Metabolism of coenzymes and prosthetic groups](http://www.bio.nite.go.jp/dogan/GeneSearchResult?GENE_LIST_TYPE=1&type=504&GENOME_LIST=n315G1&CLASS_ID=32.05&WITH_GENE_MAP=1) |
| SA0820 | *glpQ* | Glycerophosphoryl diester phosphodiesterase | -4.5 | 0.00 | [Metabolism of lipids](http://www.bio.nite.go.jp/dogan/GeneSearchResult?GENE_LIST_TYPE=1&type=504&GENOME_LIST=n315G1&CLASS_ID=32.04&WITH_GENE_MAP=1) |
| SA1548 |  | Hypothetical protein, similar to acylglycerol-3-phosphate O-acyltransfera homolog | -3.3 | 0.00 | [Metabolism of lipids](http://www.bio.nite.go.jp/dogan/GeneSearchResult?GENE_LIST_TYPE=1&type=504&GENOME_LIST=n315G1&CLASS_ID=32.04&WITH_GENE_MAP=1) |
| SA2080 |  | Hypothetical protein, similar to butyryl-CoA dehydrogenase | -3.2 | 0.00 | [Metabolism of lipids](http://www.bio.nite.go.jp/dogan/GeneSearchResult?GENE_LIST_TYPE=1&type=504&GENOME_LIST=n315G1&CLASS_ID=32.04&WITH_GENE_MAP=1) |
| SA1104 | *cdsA* | Phosphatidate cytidylyltransferase | -2.4 | 0.11 | [Metabolism of lipids](http://www.bio.nite.go.jp/dogan/GeneSearchResult?GENE_LIST_TYPE=1&type=504&GENOME_LIST=n315G1&CLASS_ID=32.04&WITH_GENE_MAP=1) |
| SA1075 | *hmrB* | HmrB protein | -2.4 | 0.07 | [Metabolism of lipids](http://www.bio.nite.go.jp/dogan/GeneSearchResult?GENE_LIST_TYPE=1&type=504&GENOME_LIST=n315G1&CLASS_ID=32.04&WITH_GENE_MAP=1) |
| SA1123 |  | Hypothetical protein, similar to 3-oxoacyl- acyl-carrier protein reductase homolog ymfI | -2.3 | 0.07 | [Metabolism of lipids](http://www.bio.nite.go.jp/dogan/GeneSearchResult?GENE_LIST_TYPE=1&type=504&GENOME_LIST=n315G1&CLASS_ID=32.04&WITH_GENE_MAP=1) |
| SA0220 |  | Hypothetical protein, similar to glycerophosphodiester phosphodiesterase | -2.1 | 0.20 | [Metabolism of lipids](http://www.bio.nite.go.jp/dogan/GeneSearchResult?GENE_LIST_TYPE=1&type=504&GENOME_LIST=n315G1&CLASS_ID=32.04&WITH_GENE_MAP=1) |
| SA0534 | *vraB* | Acetyl-CoA c-acetyltransferase | -2.0 | 0.19 | [Metabolism of lipids](http://www.bio.nite.go.jp/dogan/GeneSearchResult?GENE_LIST_TYPE=1&type=504&GENOME_LIST=n315G1&CLASS_ID=32.04&WITH_GENE_MAP=1) |
| SA2080 |  | Hypothetical protein, similar to butyryl-CoA dehydrogenase | -2.0 | 0.19 | [Metabolism of lipids](http://www.bio.nite.go.jp/dogan/GeneSearchResult?GENE_LIST_TYPE=1&type=504&GENOME_LIST=n315G1&CLASS_ID=32.04&WITH_GENE_MAP=1) |
| SA2240 |  | Hypothetical protein, similar to para-nitrobenzyl esterase chain A | 2.0 | 0.38 | [Metabolism of lipids](http://www.bio.nite.go.jp/dogan/GeneSearchResult?GENE_LIST_TYPE=1&type=504&GENOME_LIST=n315G1&CLASS_ID=32.04&WITH_GENE_MAP=1) |
| SA1584 |  | Lysophospholipase homolog | 2.4 | 0.11 | [Metabolism of lipids](http://www.bio.nite.go.jp/dogan/GeneSearchResult?GENE_LIST_TYPE=1&type=504&GENOME_LIST=n315G1&CLASS_ID=32.04&WITH_GENE_MAP=1) |
| SA1542 |  | Hypothetical protein, similar to glycerophosphoryl diester phosphodiesterase | 2.9 | 0.00 | [Metabolism of lipids](http://www.bio.nite.go.jp/dogan/GeneSearchResult?GENE_LIST_TYPE=1&type=504&GENOME_LIST=n315G1&CLASS_ID=32.04&WITH_GENE_MAP=1) |
| SA0022 |  | Hypothetical protein, similar to 5’-nucleotidase | -8.6 | 0.00 | [Metabolism of nucleotides and nucleic acids](http://www.bio.nite.go.jp/dogan/GeneSearchResult?GENE_LIST_TYPE=1&type=504&GENOME_LIST=n315G1&CLASS_ID=32.03&WITH_GENE_MAP=1) |
| SA0373 | *xprT* | Xanthine phosphoribosyltransferase | -8.6 | 0.00 | [Metabolism of nucleotides and nucleic acids](http://www.bio.nite.go.jp/dogan/GeneSearchResult?GENE_LIST_TYPE=1&type=504&GENOME_LIST=n315G1&CLASS_ID=32.03&WITH_GENE_MAP=1) |
| SA0022 |  | Hypothetical protein, similar to 5’-nucleotidase | -8.3 | 0.00 | [Metabolism of nucleotides and nucleic acids](http://www.bio.nite.go.jp/dogan/GeneSearchResult?GENE_LIST_TYPE=1&type=504&GENOME_LIST=n315G1&CLASS_ID=32.03&WITH_GENE_MAP=1) |
| SA1172 |  | Hypothetical protein, similar to GMP reductase | -6.8 | 0.00 | [Metabolism of nucleotides and nucleic acids](http://www.bio.nite.go.jp/dogan/GeneSearchResult?GENE_LIST_TYPE=1&type=504&GENOME_LIST=n315G1&CLASS_ID=32.03&WITH_GENE_MAP=1) |
| SA0016 | *purA* | Adenylosuccinate synthase | -5.4 | 0.00 | [Metabolism of nucleotides and nucleic acids](http://www.bio.nite.go.jp/dogan/GeneSearchResult?GENE_LIST_TYPE=1&type=504&GENOME_LIST=n315G1&CLASS_ID=32.03&WITH_GENE_MAP=1) |
| SA1301 | *ndk* | Nucleoside diphosphate kinase | -5.2 | 0.00 | [Metabolism of nucleotides and nucleic acids](http://www.bio.nite.go.jp/dogan/GeneSearchResult?GENE_LIST_TYPE=1&type=504&GENOME_LIST=n315G1&CLASS_ID=32.03&WITH_GENE_MAP=1) |
| SA0687 | *nrdF* | Ribonucleoside-diphosphate reductase minor subunit | -3.4 | 0.00 | [Metabolism of nucleotides and nucleic acids](http://www.bio.nite.go.jp/dogan/GeneSearchResult?GENE_LIST_TYPE=1&type=504&GENOME_LIST=n315G1&CLASS_ID=32.03&WITH_GENE_MAP=1) |
| SA1013 |  | Hypothetical protein, similar to carbamate kinase | -3.0 | 0.00 | [Metabolism of nucleotides and nucleic acids](http://www.bio.nite.go.jp/dogan/GeneSearchResult?GENE_LIST_TYPE=1&type=504&GENOME_LIST=n315G1&CLASS_ID=32.03&WITH_GENE_MAP=1) |
| SA2078 |  | Hypothetical protein, similar to inosine-adenosine-guanosine-nucleoside hydrolase; IAG-nucleoside hydrolase | -2.9 | 0.00 | [Metabolism of nucleotides and nucleic acids](http://www.bio.nite.go.jp/dogan/GeneSearchResult?GENE_LIST_TYPE=1&type=504&GENOME_LIST=n315G1&CLASS_ID=32.03&WITH_GENE_MAP=1) |
| SA0376 | *guaA* | GMP synthase (glutamine-hydrolyzing) | -2.9 | 0.00 | [Metabolism of nucleotides and nucleic acids](http://www.bio.nite.go.jp/dogan/GeneSearchResult?GENE_LIST_TYPE=1&type=504&GENOME_LIST=n315G1&CLASS_ID=32.03&WITH_GENE_MAP=1) |
| SA1160 | *nuc* | Thermonuclease | -2.7 | 0.07 | [Metabolism of nucleotides and nucleic acids](http://www.bio.nite.go.jp/dogan/GeneSearchResult?GENE_LIST_TYPE=1&type=504&GENOME_LIST=n315G1&CLASS_ID=32.03&WITH_GENE_MAP=1) |
| SA0515 |  | Hypothetical protein, similar to deoxypurine kinase | -2.7 | 0.07 | [Metabolism of nucleotides and nucleic acids](http://www.bio.nite.go.jp/dogan/GeneSearchResult?GENE_LIST_TYPE=1&type=504&GENOME_LIST=n315G1&CLASS_ID=32.03&WITH_GENE_MAP=1) |
| SA1461 | *apt* | Adenine phosphoribosyl transferase | -2.6 | 0.07 | [Metabolism of nucleotides and nucleic acids](http://www.bio.nite.go.jp/dogan/GeneSearchResult?GENE_LIST_TYPE=1&type=504&GENOME_LIST=n315G1&CLASS_ID=32.03&WITH_GENE_MAP=1) |
| SA1929 | *ctrA* | CTP synthase | -2.3 | 0.07 | [Metabolism of nucleotides and nucleic acids](http://www.bio.nite.go.jp/dogan/GeneSearchResult?GENE_LIST_TYPE=1&type=504&GENOME_LIST=n315G1&CLASS_ID=32.03&WITH_GENE_MAP=1) |
| SA0918 | *purC* | Phosphoribosylaminoimidazolesuccinocarboxamide synthetase homolog | -2.3 | 0.13 | [Metabolism of nucleotides and nucleic acids](http://www.bio.nite.go.jp/dogan/GeneSearchResult?GENE_LIST_TYPE=1&type=504&GENOME_LIST=n315G1&CLASS_ID=32.03&WITH_GENE_MAP=1) |
| SA1427 | *pfs* | 5’-Methylthioadenosine nucleosidase/S-adenosylhomocysteine nucleosidase | -2.2 | 0.13 | [Metabolism of nucleotides and nucleic acids](http://www.bio.nite.go.jp/dogan/GeneSearchResult?GENE_LIST_TYPE=1&type=504&GENOME_LIST=n315G1&CLASS_ID=32.03&WITH_GENE_MAP=1) |
| SA0375 | *guaB* | Inositol-monophosphate dehydrogenase | -2.2 | 0.13 | [Metabolism of nucleotides and nucleic acids](http://www.bio.nite.go.jp/dogan/GeneSearchResult?GENE_LIST_TYPE=1&type=504&GENOME_LIST=n315G1&CLASS_ID=32.03&WITH_GENE_MAP=1) |
| SA0511 |  | Hypothetical protein, similar to UDP-glucose 4-epimerase related protein | -2.2 | 0.13 | [Metabolism of nucleotides and nucleic acids](http://www.bio.nite.go.jp/dogan/GeneSearchResult?GENE_LIST_TYPE=1&type=504&GENOME_LIST=n315G1&CLASS_ID=32.03&WITH_GENE_MAP=1) |
| SA0917 | *purK* | Phosphoribosylaminoimidazole carboxylase carbon dioxide-fixation chain PurK homolog | -2.1 | 0.25 | [Metabolism of nucleotides and nucleic acids](http://www.bio.nite.go.jp/dogan/GeneSearchResult?GENE_LIST_TYPE=1&type=504&GENOME_LIST=n315G1&CLASS_ID=32.03&WITH_GENE_MAP=1) |
| SA0686 | *nrdE* | Ribonuceloside diphosphate reductase major subunit | -2.0 | 0.19 | [Metabolism of nucleotides and nucleic acids](http://www.bio.nite.go.jp/dogan/GeneSearchResult?GENE_LIST_TYPE=1&type=504&GENOME_LIST=n315G1&CLASS_ID=32.03&WITH_GENE_MAP=1) |
| SA0453 |  | Hypothetical protein, similar to 4-diphosphocytidyl-2-C-methyl-D-erythritol kinase | -2.0 | 0.19 | [Metabolism of nucleotides and nucleic acids](http://www.bio.nite.go.jp/dogan/GeneSearchResult?GENE_LIST_TYPE=1&type=504&GENOME_LIST=n315G1&CLASS_ID=32.03&WITH_GENE_MAP=1) |
| SA0134 | *drm* | Phosphopentomutase (EC 5.4.2.7) | 2.0 | 0.61 | [Metabolism of nucleotides and nucleic acids](http://www.bio.nite.go.jp/dogan/GeneSearchResult?GENE_LIST_TYPE=1&type=504&GENOME_LIST=n315G1&CLASS_ID=32.03&WITH_GENE_MAP=1) |
| SA0864 |  | GTP pyrophosphokinase | 2.1 | 0.25 | [Metabolism of nucleotides and nucleic acids](http://www.bio.nite.go.jp/dogan/GeneSearchResult?GENE_LIST_TYPE=1&type=504&GENOME_LIST=n315G1&CLASS_ID=32.03&WITH_GENE_MAP=1) |
| SA2409 |  | Hypothetical protein, similar to anaerobic ribonucleotide reductase activator protein | 2.2 | 0.20 | [Metabolism of nucleotides and nucleic acids](http://www.bio.nite.go.jp/dogan/GeneSearchResult?GENE_LIST_TYPE=1&type=504&GENOME_LIST=n315G1&CLASS_ID=32.03&WITH_GENE_MAP=1) |
| SA0816 |  | Hypothetical protein, similar to polyribonucleotide nucleotidyltransferase | 2.7 | 0.06 | [Metabolism of nucleotides and nucleic acids](http://www.bio.nite.go.jp/dogan/GeneSearchResult?GENE_LIST_TYPE=1&type=504&GENOME_LIST=n315G1&CLASS_ID=32.03&WITH_GENE_MAP=1) |
| SA0131 | *pnp* | Purine nucleoside phosphorylase | 7.9 | 0.00 | [Metabolism of nucleotides and nucleic acids](http://www.bio.nite.go.jp/dogan/GeneSearchResult?GENE_LIST_TYPE=1&type=504&GENOME_LIST=n315G1&CLASS_ID=32.03&WITH_GENE_MAP=1) |
| SA0022 |  | Hypothetical protein, similar to 5’-nucleotidase | -8.4 | 0.00 | [Metabolism of nucleotides and nucleic acids](http://www.bio.nite.go.jp/dogan/GeneSearchResult?GENE_LIST_TYPE=1&type=504&GENOME_LIST=n315G1&CLASS_ID=32.03&WITH_GENE_MAP=1) |
| SA0022 |  | Hypothetical protein, similar to 5’-nucleotidase | -5.9 | 0.00 | [Metabolism of nucleotides and nucleic acids](http://www.bio.nite.go.jp/dogan/GeneSearchResult?GENE_LIST_TYPE=1&type=504&GENOME_LIST=n315G1&CLASS_ID=32.03&WITH_GENE_MAP=1) |
| SA1921 | *tdk* | Thymidine kinase | -2.6 | 0.00 | [Metabolism of nucleotides and nucleic acids](http://www.bio.nite.go.jp/dogan/GeneSearchResult?GENE_LIST_TYPE=1&type=504&GENOME_LIST=n315G1&CLASS_ID=32.03&WITH_GENE_MAP=1) |
| SA2297 |  | Hypothetical protein, similar to GTP-pyrophosphokinase | 9.7 | 0.00 | [Metabolism of nucleotides and nucleic acids](http://www.bio.nite.go.jp/dogan/GeneSearchResult?GENE_LIST_TYPE=1&type=504&GENOME_LIST=n315G1&CLASS_ID=32.03&WITH_GENE_MAP=1) |
| SA0881 |  | Hypothetical protein, similar to nucleotidase | 2.1 | 0.25 | [Metabolism of phosphate](http://www.bio.nite.go.jp/dogan/GeneSearchResult?GENE_LIST_TYPE=1&type=504&GENOME_LIST=n315G1&CLASS_ID=32.06&WITH_GENE_MAP=1) |
| SA1237 |  | Hypothetical protein, similar to 5-bromo-4-chloroindolyl phosphate hydrolysis protein xpaC | 2.9 | 0.00 | [Metabolism of phosphate](http://www.bio.nite.go.jp/dogan/GeneSearchResult?GENE_LIST_TYPE=1&type=504&GENOME_LIST=n315G1&CLASS_ID=32.06&WITH_GENE_MAP=1) |
| SA2301 |  | Hypothetical protein, similar to alkaline phosphatase | 3.5 | 0.00 | [Metabolism of phosphate](http://www.bio.nite.go.jp/dogan/GeneSearchResult?GENE_LIST_TYPE=1&type=504&GENOME_LIST=n315G1&CLASS_ID=32.06&WITH_GENE_MAP=1) |
| SA0530 |  | Hypothetical protein, similar to indigoidine systhesis protein | -4.0 | 0.00 | [Miscellaneous](http://www.bio.nite.go.jp/dogan/GeneSearchResult?GENE_LIST_TYPE=1&type=504&GENOME_LIST=n315G1&CLASS_ID=34.07&WITH_GENE_MAP=1) |
| SA1193 | *fmtC* | Oxacillin resistance-related FmtC protein | -2.9 | 0.00 | [Miscellaneous](http://www.bio.nite.go.jp/dogan/GeneSearchResult?GENE_LIST_TYPE=1&type=504&GENOME_LIST=n315G1&CLASS_ID=34.07&WITH_GENE_MAP=1) |
| SA0516 |  | Hypothetical protein, similar to Cu binding protein (Mn oxidation) | -2.7 | 0.00 | [Miscellaneous](http://www.bio.nite.go.jp/dogan/GeneSearchResult?GENE_LIST_TYPE=1&type=504&GENOME_LIST=n315G1&CLASS_ID=34.07&WITH_GENE_MAP=1) |
| SA0516 |  | Hypothetical protein, similar to Cu binding protein (Mn oxidation) | -2.4 | 0.07 | [Miscellaneous](http://www.bio.nite.go.jp/dogan/GeneSearchResult?GENE_LIST_TYPE=1&type=504&GENOME_LIST=n315G1&CLASS_ID=34.07&WITH_GENE_MAP=1) |
| SA0914 |  | Hypothetical protein, similar to chitinase B | 3.0 | 0.00 | [Miscellaneous](http://www.bio.nite.go.jp/dogan/GeneSearchResult?GENE_LIST_TYPE=1&type=504&GENOME_LIST=n315G1&CLASS_ID=34.07&WITH_GENE_MAP=1) |
| SA0231 |  | Hypothetical protein, similar to flavohemoprotein | 3.0 | 0.00 | [Miscellaneous](http://www.bio.nite.go.jp/dogan/GeneSearchResult?GENE_LIST_TYPE=1&type=504&GENOME_LIST=n315G1&CLASS_ID=34.07&WITH_GENE_MAP=1) |
| SA0797 |  | Hypothetical protein, similar to nitrogen fixation protein NifU | 3.5 | 0.00 | [Miscellaneous](http://www.bio.nite.go.jp/dogan/GeneSearchResult?GENE_LIST_TYPE=1&type=504&GENOME_LIST=n315G1&CLASS_ID=34.07&WITH_GENE_MAP=1) |
| SA0482 |  | Hypothetical protein, similar to creatine kinase | 4.4 | 0.00 | [Miscellaneous](http://www.bio.nite.go.jp/dogan/GeneSearchResult?GENE_LIST_TYPE=1&type=504&GENOME_LIST=n315G1&CLASS_ID=34.07&WITH_GENE_MAP=1) |
| SA0914 |  | Hypothetical protein, similar to chitinase B | 5.2 | 0.00 | [Miscellaneous](http://www.bio.nite.go.jp/dogan/GeneSearchResult?GENE_LIST_TYPE=1&type=504&GENOME_LIST=n315G1&CLASS_ID=34.07&WITH_GENE_MAP=1) |
| SA0268 |  | Hypothetical protein | -31.4 | 0.00 | No similarity |
| SA0262 |  | Hypothetical protein | -14.9 | 0.00 | No similarity |
| SA0273 |  | Hypothetical protein | -9.4 | 0.00 | No similarity |
| SA0792 |  | Hypothetical protein | -7.0 | 0.00 | No similarity |
| SA1056 |  | Hypothetical protein | -6.9 | 0.00 | No similarity |
| SA1726 | *scpB* | Hypothetical protein | -6.8 | 0.00 | No similarity |
| SA0276 |  | Hypothetical protein | -6.7 | 0.00 | No similarity |
| SA0663 |  | Hypothetical protein | -6.7 | 0.00 | No similarity |
| SA2126 |  | Hypothetical protein | -6.3 | 0.00 | No similarity |
| SA1619 |  | Hypothetical protein | -5.0 | 0.00 | No similarity |
| SA1017 |  | Hypothetical protein | -4.5 | 0.00 | No similarity |
| SA2173 |  | Hypothetical protein | -4.3 | 0.00 | No similarity |
| SA1621 |  | Hypothetical protein | -4.3 | 0.00 | No similarity |
| SA1620 |  | Hypothetical protein | -3.9 | 0.00 | No similarity |
| SA0164 |  | Hypothetical protein | -3.7 | 0.00 | No similarity |
| SA1665 |  | Hypothetical protein | -3.5 | 0.00 | No similarity |
| SA0285 |  | Hypothetical protein | -3.3 | 0.00 | No similarity |
| SA1944 |  | Hypothetical protein | -3.3 | 0.00 | No similarity |
| SA0889 |  | Hypothetical protein | -3.2 | 0.00 | No similarity |
| SA0396 | *lpl1* | Hypothetical protein [Pathogenicity island SaPIn2] | -2.9 | 0.00 | No similarity |
| SA0090 |  | Hypothetical protein | -2.9 | 0.00 | No similarity |
| SA0403 | *lpl7* | Hypothetical protein [Pathogenicity island SaPIn2] | -2.9 | 0.00 | No similarity |
| SA0397 | *lpl2* | Hypothetical protein [Pathogenicity island SaPIn2] | -2.8 | 0.00 | No similarity |
| SA0539 |  | Hypothetical protein | -2.7 | 0.00 | No similarity |
| SA0292 |  | Hypothetical protein | -2.7 | 0.07 | No similarity |
| SA2141 |  | Hypothetical protein | -2.7 | 0.07 | No similarity |
| SA0404 | *lpl8* | Hypothetical protein [Pathogenicity island SaPIn2] | -2.5 | 0.07 | No similarity |
| SA2352 |  | Hypothetical protein | -2.5 | 0.07 | No similarity |
| SA1590 |  | Hypothetical protein | -2.5 | 0.07 | No similarity |
| SA2497 |  | Hypothetical protein | -2.5 | 0.07 | No similarity |
| SA0651 |  | Hypothetical protein | -2.5 | 0.07 | No similarity |
| SA0364 |  | Hypothetical protein | -2.5 | 0.07 | No similarity |
| SA0535 | *vraC* | Hypothetical protein | -2.5 | 0.07 | No similarity |
| SA1015 |  | Hypothetical protein | -2.5 | 0.07 | No similarity |
| SA2445 |  | Hypothetical protein | -2.4 | 0.07 | No similarity |
| SAS026 |  | Hypothetical protein | -2.3 | 0.13 | No similarity |
| SA0300 |  | Truncated hypothetical protein | -2.3 | 0.07 | No similarity |
| SA0291 |  | Hypothetical protein | -2.3 | 0.11 | No similarity |
| SA2444 |  | Hypothetical protein | -2.3 | 0.07 | No similarity |
| SA0121 |  | Hypothetical protein | -2.3 | 0.07 | No similarity |
| SA0129 |  | Hypothetical protein | -2.2 | 0.13 | No similarity |
| SA2443 |  | Hypothetical protein | -2.2 | 0.13 | No similarity |
| SA2496 |  | Hypothetical protein | -2.2 | 0.13 | No similarity |
| SA0623 |  | Hypothetical protein | -2.1 | 0.20 | No similarity |
| SA0732 |  | Hypothetical protein | -2.0 | 0.19 | No similarity |
| SA1317 |  | Hypothetical protein | -2.0 | 0.25 | No similarity |
| SA0613 |  | Hypothetical protein | 2.0 | 0.38 | No similarity |
| SA0931 |  | Hypothetical protein | 2.0 | 0.38 | No similarity |
| SA0779 |  | Hypothetical protein | 2.0 | 0.61 | No similarity |
| SAS068 |  | Hypothetical protein | 2.1 | 0.38 | No similarity |
| SA0315 |  | Hypothetical protein | 2.2 | 0.20 | No similarity |
| SA0570 |  | Hypothetical protein | 2.3 | 0.19 | No similarity |
| SA2168 |  | Hypothetical protein | 2.3 | 0.20 | No similarity |
| SA2116 |  | Hypothetical protein | 2.3 | 0.19 | No similarity |
| SA0664 |  | Hypothetical protein | 2.3 | 0.25 | No similarity |
| SAS016 |  | Hypothetical protein | 2.4 | 0.13 | No similarity |
| SA0571 |  | Hypothetical protein | 2.4 | 0.19 | No similarity |
| SA2221 |  | Hypothetical protein | 2.5 | 0.06 | No similarity |
| SA1594 |  | Hypothetical protein | 2.5 | 0.06 | No similarity |
| SA1546 |  | Hypothetical protein | 2.5 | 0.11 | No similarity |
| SA2401 |  | Hypothetical protein | 2.5 | 0.11 | No similarity |
| SA0608 |  | Hypothetical protein | 2.6 | 0.06 | No similarity |
| SA1567 |  | Hypothetical protein | 2.6 | 0.06 | No similarity |
| SA1215 |  | Hypothetical protein | 2.6 | 0.11 | No similarity |
| SA1161 |  | Hypothetical protein | 2.8 | 0.00 | No similarity |
| SA1284 |  | Hypothetical protein | 2.8 | 0.06 | No similarity |
| SA1210 |  | Hypothetical protein | 2.9 | 0.00 | No similarity |
| SAS056 |  | Hypothetical protein | 3.2 | 0.00 | No similarity |
| SAS010 |  | Hypothetical protein | 3.4 | 0.00 | No similarity |
| SA2049 |  | Hypothetical protein | 3.4 | 0.00 | No similarity |
| SA2292 |  | Hypothetical protein | 4.0 | 0.00 | No similarity |
| SA1361 |  | Hypothetical protein | 5.0 | 0.00 | No similarity |
| SA1362 |  | Hypothetical protein | 5.2 | 0.00 | No similarity |
| SA2139 |  | Hypothetical protein | 5.3 | 0.00 | No similarity |
| SA0955 |  | Hypothetical protein | 5.4 | 0.00 | No similarity |
| SA2113 |  | Hypothetical protein | 6.3 | 0.00 | No similarity |
| SA1825 |  | Hypothetical protein [Pathogenicity island SaPIn1] | 6.9 | 0.00 | No similarity |
| SA1824 |  | Hypothetical protein [Pathogenicity island SaPIn1] | 7.3 | 0.00 | No similarity |
| SAS037 |  | Hypothetical protein | 7.8 | 0.00 | No similarity |
| SA1703 |  | Hypothetical protein | 7.9 | 0.00 | No similarity |
| SA1823 |  | Hypothetical protein [Pathogenicity island SaPIn1] | 7.9 | 0.00 | No similarity |
| SA2224 |  | Hypothetical protein | 8.0 | 0.00 | No similarity |
| SA1822 |  | Hypothetical protein [Pathogenicity island SaPIn1] | 8.9 | 0.00 | No similarity |
| SA0591 |  | Hypothetical protein | 9.0 | 0.00 | No similarity |
| SA1821 |  | Hypothetical protein [Pathogenicity island SaPIn1] | 9.2 | 0.00 | No similarity |
| SA0883 |  | Hypothetical protein | 9.4 | 0.00 | No similarity |
| SA1476 |  | Hypothetical protein | 10.3 | 0.00 | No similarity |
| SA2343 |  | Hypothetical protein | 102.4 | 0.00 | No similarity |
| SA0746 |  | Staphylococcal nuclease | -30.3 | 0.00 | Pathogenic factors (toxins and colonization factors) |
| SA0977 | *isdA* | Cell surface protein | -17.3 | 0.00 | [Pathogenic factors (toxins and colonization factors)](http://www.bio.nite.go.jp/dogan/GeneSearchResult?GENE_LIST_TYPE=1&type=504&GENOME_LIST=n315G1&CLASS_ID=34.06&WITH_GENE_MAP=1) |
| SA1648 | *seo* | Enterotoxin SeO [Pathogenicity island SaPIn3] | -14.4 | 0.00 | [Pathogenic factors (toxins and colonization factors)](http://www.bio.nite.go.jp/dogan/GeneSearchResult?GENE_LIST_TYPE=1&type=504&GENOME_LIST=n315G1&CLASS_ID=34.06&WITH_GENE_MAP=1) |
| SA2097 |  | Hypothetical protein, similar to secretory antigen precursor SsaA | -13.3 | 0.00 | [Pathogenic factors (toxins and colonization factors)](http://www.bio.nite.go.jp/dogan/GeneSearchResult?GENE_LIST_TYPE=1&type=504&GENOME_LIST=n315G1&CLASS_ID=34.06&WITH_GENE_MAP=1) |
| SA2356 | *isaA* | Immunodominant antigen A | -11.5 | 0.00 | [Pathogenic factors (toxins and colonization factors)](http://www.bio.nite.go.jp/dogan/GeneSearchResult?GENE_LIST_TYPE=1&type=504&GENOME_LIST=n315G1&CLASS_ID=34.06&WITH_GENE_MAP=1) |
| SA1725 | *scpA* | Staphopain, Cysteine Proteinase | -8.8 | 0.00 | Pathogenic factors (toxins and colonization factors) |
| SAS065 | *RNAⅢ* | Delta-hemolysin | -8.3 | 0.00 | [Pathogenic factors (toxins and colonization factors)](http://www.bio.nite.go.jp/dogan/GeneSearchResult?GENE_LIST_TYPE=1&type=504&GENOME_LIST=n315G1&CLASS_ID=34.06&WITH_GENE_MAP=1) |
| SA0276 |  | Conserved hypothetical protein, similar to diarrheal toxin | -8.1 | 0.00 | [Pathogenic factors (toxins and colonization factors)](http://www.bio.nite.go.jp/dogan/GeneSearchResult?GENE_LIST_TYPE=1&type=504&GENOME_LIST=n315G1&CLASS_ID=34.06&WITH_GENE_MAP=1) |
| SA2093 | *ssaA* | Secretory antigen precursor SsaA homolog | -7.8 | 0.00 | [Pathogenic factors (toxins and colonization factors)](http://www.bio.nite.go.jp/dogan/GeneSearchResult?GENE_LIST_TYPE=1&type=504&GENOME_LIST=n315G1&CLASS_ID=34.06&WITH_GENE_MAP=1) |
| SA0620 |  | Secretory antigen SsaA homologue | -5.9 | 0.00 | [Pathogenic factors (toxins and colonization factors)](http://www.bio.nite.go.jp/dogan/GeneSearchResult?GENE_LIST_TYPE=1&type=504&GENOME_LIST=n315G1&CLASS_ID=34.06&WITH_GENE_MAP=1) |
| SA2353 |  | Hypothetical protein, similar to secretory antigen precursor SsaA | -5.8 | 0.00 | [Pathogenic factors (toxins and colonization factors)](http://www.bio.nite.go.jp/dogan/GeneSearchResult?GENE_LIST_TYPE=1&type=504&GENOME_LIST=n315G1&CLASS_ID=34.06&WITH_GENE_MAP=1) |
| SA0901 | *sspA* | Serine protease; V8 protease; glutamyl endopeptidase | -5.4 | 0.00 | [Pathogenic factors (toxins and colonization factors)](http://www.bio.nite.go.jp/dogan/GeneSearchResult?GENE_LIST_TYPE=1&type=504&GENOME_LIST=n315G1&CLASS_ID=34.06&WITH_GENE_MAP=1) |
| SA1000 |  | Hypothetical protein, similar to fibrinogen-binding protein | -4.5 | 0.00 | [Pathogenic factors (toxins and colonization factors)](http://www.bio.nite.go.jp/dogan/GeneSearchResult?GENE_LIST_TYPE=1&type=504&GENOME_LIST=n315G1&CLASS_ID=34.06&WITH_GENE_MAP=1) |
| SA2206 | *sbi* | IgG-binding protein SBI | -4.1 | 0.00 | [Pathogenic factors (toxins and colonization factors)](http://www.bio.nite.go.jp/dogan/GeneSearchResult?GENE_LIST_TYPE=1&type=504&GENOME_LIST=n315G1&CLASS_ID=34.06&WITH_GENE_MAP=1) |
| SA0309 | *geh* | Glycerol ester hydrolase | -3.9 | 0.00 | [Pathogenic factors (toxins and colonization factors)](http://www.bio.nite.go.jp/dogan/GeneSearchResult?GENE_LIST_TYPE=1&type=504&GENOME_LIST=n315G1&CLASS_ID=34.06&WITH_GENE_MAP=1) |
| SA2423 | *clfB* | Clumping factor B | -3.7 | 0.00 | [Pathogenic factors (toxins and colonization factors)](http://www.bio.nite.go.jp/dogan/GeneSearchResult?GENE_LIST_TYPE=1&type=504&GENOME_LIST=n315G1&CLASS_ID=34.06&WITH_GENE_MAP=1) |
| SA0879 | *htrA* | Serine protease HtrA | -3.3 | 0.00 | [Pathogenic factors (toxins and colonization factors)](http://www.bio.nite.go.jp/dogan/GeneSearchResult?GENE_LIST_TYPE=1&type=504&GENOME_LIST=n315G1&CLASS_ID=34.06&WITH_GENE_MAP=1) |
| SA0091 | *plc* | 1-Phosphatidylinositol phosphodiesterase precurosr | -3.2 | 0.00 | [Pathogenic factors (toxins and colonization factors)](http://www.bio.nite.go.jp/dogan/GeneSearchResult?GENE_LIST_TYPE=1&type=504&GENOME_LIST=n315G1&CLASS_ID=34.06&WITH_GENE_MAP=1) |
| SA0520 | *sdrD* | Ser-Asp rich fibrinogen-binding, bone sialoprotein-binding protein | -3.2 | 0.00 | [Pathogenic factors (toxins and colonization factors)](http://www.bio.nite.go.jp/dogan/GeneSearchResult?GENE_LIST_TYPE=1&type=504&GENOME_LIST=n315G1&CLASS_ID=34.06&WITH_GENE_MAP=1) |
| SA1644 | *yent2* | Enterotoxin YENT2 [Pathogenicity island SaPIn3] | -3.0 | 0.00 | [Pathogenic factors (toxins and colonization factors)](http://www.bio.nite.go.jp/dogan/GeneSearchResult?GENE_LIST_TYPE=1&type=504&GENOME_LIST=n315G1&CLASS_ID=34.06&WITH_GENE_MAP=1) |
| SA1898 | *sceD* | Hypothetical protein, imilar to SceD precursor | -3.0 | 0.00 | [Pathogenic factors (toxins and colonization factors)](http://www.bio.nite.go.jp/dogan/GeneSearchResult?GENE_LIST_TYPE=1&type=504&GENOME_LIST=n315G1&CLASS_ID=34.06&WITH_GENE_MAP=1) |
| SA2091 | *fnb* | Hypothetical protein | -3.0 | 0.00 | Pathogenic factors (toxins and colonization factors) |
| SA1647 | *sem* | Enterotoxin SEM [Pathogenicity island SaPIn3] | -2.9 | 0.07 | [Pathogenic factors (toxins and colonization factors)](http://www.bio.nite.go.jp/dogan/GeneSearchResult?GENE_LIST_TYPE=1&type=504&GENOME_LIST=n315G1&CLASS_ID=34.06&WITH_GENE_MAP=1) |
| SA0900 | *sspB* | Cysteine protease precursor | -2.6 | 0.07 | [Pathogenic factors (toxins and colonization factors)](http://www.bio.nite.go.jp/dogan/GeneSearchResult?GENE_LIST_TYPE=1&type=504&GENOME_LIST=n315G1&CLASS_ID=34.06&WITH_GENE_MAP=1) |
| SA1003 |  | Hypothetical protein, similar to fibrinogen-binding protein | -2.5 | 0.07 | [Pathogenic factors (toxins and colonization factors)](http://www.bio.nite.go.jp/dogan/GeneSearchResult?GENE_LIST_TYPE=1&type=504&GENOME_LIST=n315G1&CLASS_ID=34.06&WITH_GENE_MAP=1) |
| SA0270 |  | Hypothetical protein, similar to secretory antigen precursor SsaA | -2.5 | 0.07 | [Pathogenic factors (toxins and colonization factors)](http://www.bio.nite.go.jp/dogan/GeneSearchResult?GENE_LIST_TYPE=1&type=504&GENOME_LIST=n315G1&CLASS_ID=34.06&WITH_GENE_MAP=1) |
| SA0521 | *sdrE* | Ser-Asp rich fibrinogen-binding, bone sialoprotein-binding protein | -2.4 | 0.11 | Pathogenic factors (toxins and colonization factors) |
| SA1007 | *hly* | Alpha-Hemolysin precursor | -2.3 | 0.13 | [Pathogenic factors (toxins and colonization factors)](http://www.bio.nite.go.jp/dogan/GeneSearchResult?GENE_LIST_TYPE=1&type=504&GENOME_LIST=n315G1&CLASS_ID=34.06&WITH_GENE_MAP=1) |
| SA1645 | *yent1* | Enterotoxin Yent1 [Pathogenicity island SaPIn3] | -2.2 | 0.13 | [Pathogenic factors (toxins and colonization factors)](http://www.bio.nite.go.jp/dogan/GeneSearchResult?GENE_LIST_TYPE=1&type=504&GENOME_LIST=n315G1&CLASS_ID=34.06&WITH_GENE_MAP=1) |
| SA1646 | *sei* | Extracellular enterotoxin type I precursor [Pathogenicity island SaPIn3] | -2.1 | 0.20 | [Pathogenic factors (toxins and colonization factors)](http://www.bio.nite.go.jp/dogan/GeneSearchResult?GENE_LIST_TYPE=1&type=504&GENOME_LIST=n315G1&CLASS_ID=34.06&WITH_GENE_MAP=1) |
| SA1751 |  | Truncated map-w protein | -2.0 | 0.20 | [Pathogenic factors (toxins and colonization factors)](http://www.bio.nite.go.jp/dogan/GeneSearchResult?GENE_LIST_TYPE=1&type=504&GENOME_LIST=n315G1&CLASS_ID=34.06&WITH_GENE_MAP=1) |
| SA2463 | *lip* | Triacylglycerol lipase (EC 3.1.1.3) precursor | 2.0 | 0.38 | [Pathogenic factors (toxins and colonization factors)](http://www.bio.nite.go.jp/dogan/GeneSearchResult?GENE_LIST_TYPE=1&type=504&GENOME_LIST=n315G1&CLASS_ID=34.06&WITH_GENE_MAP=1) |
| SA0102 |  | 67 kDa Myosin-crossreactive streptococcal antigen homologue | 2.1 | 0.25 | [Pathogenic factors (toxins and colonization factors)](http://www.bio.nite.go.jp/dogan/GeneSearchResult?GENE_LIST_TYPE=1&type=504&GENOME_LIST=n315G1&CLASS_ID=34.06&WITH_GENE_MAP=1) |
| SA2090 |  | Conserved hypothetical protein | 2.1 | 0.61 | Pathogenic factors (toxins and colonization factors) |
| SA1969 |  | Hypothetical protein, similar to ATP-binding Mrp-like protein | 2.2 | 0.20 | [Pathogenic factors (toxins and colonization factors)](http://www.bio.nite.go.jp/dogan/GeneSearchResult?GENE_LIST_TYPE=1&type=504&GENOME_LIST=n315G1&CLASS_ID=34.06&WITH_GENE_MAP=1) |
| SA2323 |  | Conserved hypothetical protein | 2.4 | 0.11 | [Pathogenic factors (toxins and colonization factors)](http://www.bio.nite.go.jp/dogan/GeneSearchResult?GENE_LIST_TYPE=1&type=504&GENOME_LIST=n315G1&CLASS_ID=34.06&WITH_GENE_MAP=1) |
| SA0742 | *clfA* | Fibrinogen-binding protein A, clumping factor | 2.5 | 0.11 | [Pathogenic factors (toxins and colonization factors)](http://www.bio.nite.go.jp/dogan/GeneSearchResult?GENE_LIST_TYPE=1&type=504&GENOME_LIST=n315G1&CLASS_ID=34.06&WITH_GENE_MAP=1) |
| SA1820 |  | Hypothetical protein, similar to bacteriophage terminase small subunit [Pathogenicity island SaPIn1] | 11.5 | 0.00 | Pathogenic factors (toxins and colonization factors) |
| SA0899 | *sspC* | Cysteine protease | -2.0 | 0.25 | Pathogenic factors(toxins and colonization factors) |
| SA1762 |  | Hypothetical protein [Bacteriophage phiN315] | -3.1 | 0.00 | [Phage-related functions](http://www.bio.nite.go.jp/dogan/GeneSearchResult?GENE_LIST_TYPE=1&type=504&GENOME_LIST=n315G1&CLASS_ID=34.04&WITH_GENE_MAP=1) |
| SA0356 |  | Truncated integrase | -2.7 | 0.00 | [Phage-related functions](http://www.bio.nite.go.jp/dogan/GeneSearchResult?GENE_LIST_TYPE=1&type=504&GENOME_LIST=n315G1&CLASS_ID=34.04&WITH_GENE_MAP=1) |
| SA1765 |  | hypothetical protein [Bacteriophage phiN315] | -2.6 | 0.07 | [Phage-related functions](http://www.bio.nite.go.jp/dogan/GeneSearchResult?GENE_LIST_TYPE=1&type=504&GENOME_LIST=n315G1&CLASS_ID=34.04&WITH_GENE_MAP=1) |
| SA1835 | *int* | Hypothetical protein, imilar to integrase [Pathogenicity island SaPIn1] | -2.6 | 0.00 | [Phage-related functions](http://www.bio.nite.go.jp/dogan/GeneSearchResult?GENE_LIST_TYPE=1&type=504&GENOME_LIST=n315G1&CLASS_ID=34.04&WITH_GENE_MAP=1) |
| SA1766 |  | Hypothetical protein [Bacteriophage phiN315] | -2.2 | 0.19 | [Phage-related functions](http://www.bio.nite.go.jp/dogan/GeneSearchResult?GENE_LIST_TYPE=1&type=504&GENOME_LIST=n315G1&CLASS_ID=34.04&WITH_GENE_MAP=1) |
| SA1095 | *xerC* | Site-specific recombinase XerC homolog | 2.3 | 0.19 | [Phage-related functions](http://www.bio.nite.go.jp/dogan/GeneSearchResult?GENE_LIST_TYPE=1&type=504&GENOME_LIST=n315G1&CLASS_ID=34.04&WITH_GENE_MAP=1) |
| SA1328 | *xerD* | Site-specific recombinase | 2.4 | 0.13 | [Phage-related functions](http://www.bio.nite.go.jp/dogan/GeneSearchResult?GENE_LIST_TYPE=1&type=504&GENOME_LIST=n315G1&CLASS_ID=34.04&WITH_GENE_MAP=1) |
| SA0754 |  | Hypothetical protein, similar to lactococcal prophage ps3 protein 05 | 2.6 | 0.13 | [Phage-related functions](http://www.bio.nite.go.jp/dogan/GeneSearchResult?GENE_LIST_TYPE=1&type=504&GENOME_LIST=n315G1&CLASS_ID=34.04&WITH_GENE_MAP=1) |
| SA0253 | *lrgB* | Antiholin-like protein LrgB | 21.5 | 0.00 | [Phage-related functions](http://www.bio.nite.go.jp/dogan/GeneSearchResult?GENE_LIST_TYPE=1&type=504&GENOME_LIST=n315G1&CLASS_ID=34.04&WITH_GENE_MAP=1) |
| SA0252 | *lrgA* | Murein hydrolase regulator LrgA | 24.2 | 0.00 | [Phage-related functions](http://www.bio.nite.go.jp/dogan/GeneSearchResult?GENE_LIST_TYPE=1&type=504&GENOME_LIST=n315G1&CLASS_ID=34.04&WITH_GENE_MAP=1) |
| SA1836 | *groEL* | GroEL protein | 2.1 | 0.38 | [Protein folding](http://www.bio.nite.go.jp/dogan/GeneSearchResult?GENE_LIST_TYPE=1&type=504&GENOME_LIST=n315G1&CLASS_ID=33.09&WITH_GENE_MAP=1) |
| SA1837 | *groES* | GroES protein | 2.3 | 0.13 | [Protein folding](http://www.bio.nite.go.jp/dogan/GeneSearchResult?GENE_LIST_TYPE=1&type=504&GENOME_LIST=n315G1&CLASS_ID=33.09&WITH_GENE_MAP=1) |
| SA1659 | *prsA* | Peptidyl-prolyl cis/trans isomerase homolog | 2.4 | 0.13 | [Protein folding](http://www.bio.nite.go.jp/dogan/GeneSearchResult?GENE_LIST_TYPE=1&type=504&GENOME_LIST=n315G1&CLASS_ID=33.09&WITH_GENE_MAP=1) |
| SA1409 | *dnaK* | DnaK protein (HSP70) | 2.4 | 0.13 | [Protein folding](http://www.bio.nite.go.jp/dogan/GeneSearchResult?GENE_LIST_TYPE=1&type=504&GENOME_LIST=n315G1&CLASS_ID=33.09&WITH_GENE_MAP=1) |
| SA1360 |  | Xaa-Pro dipeptidase | -3.6 | 0.00 | [Protein modification](http://www.bio.nite.go.jp/dogan/GeneSearchResult?GENE_LIST_TYPE=1&type=504&GENOME_LIST=n315G1&CLASS_ID=33.08&WITH_GENE_MAP=1) |
| SA1063 |  | Protein kinase | -2.7 | 0.00 | [Protein modification](http://www.bio.nite.go.jp/dogan/GeneSearchResult?GENE_LIST_TYPE=1&type=504&GENOME_LIST=n315G1&CLASS_ID=34.06&WITH_GENE_MAP=1) |
| SA1039 | *lsp* | Lipoprotein signal peptidase | -2.2 | 0.11 | [Protein modification](http://www.bio.nite.go.jp/dogan/GeneSearchResult?GENE_LIST_TYPE=1&type=504&GENOME_LIST=n315G1&CLASS_ID=34.06&WITH_GENE_MAP=1) |
| SA1194 | *msrA2* | Peptide methionine sulfoxide reductase homolog | 3.3 | 0.00 | [Protein modification](http://www.bio.nite.go.jp/dogan/GeneSearchResult?GENE_LIST_TYPE=1&type=504&GENOME_LIST=n315G1&CLASS_ID=34.06&WITH_GENE_MAP=1) |
| SA1063 |  | Protein kinase | -2.7 | 0.00 | [Protein modification](http://www.bio.nite.go.jp/dogan/GeneSearchResult?GENE_LIST_TYPE=1&type=504&GENOME_LIST=n315G1&CLASS_ID=33.08&WITH_GENE_MAP=1) |
| SA2446 |  | Hypothetical protein, similar to preprotein translocase secY | -3.4 | 0.00 | [Protein secretion](http://www.bio.nite.go.jp/dogan/GeneSearchResult?GENE_LIST_TYPE=1&type=504&GENOME_LIST=n315G1&CLASS_ID=31.06&WITH_GENE_MAP=1) |
| SA1502 | *rplT* | 50S ribosomal protein L20 | -8.8 | 0.00 | [Protein synthesis](http://www.bio.nite.go.jp/dogan/GeneSearchResult?GENE_LIST_TYPE=1&type=504&GENOME_LIST=n315G1&CLASS_ID=33.07&WITH_GENE_MAP=1) |
| SA0354 | *rpsR* | 30S ribosomal protein S18 | -6.4 | 0.00 | [Protein synthesis](http://www.bio.nite.go.jp/dogan/GeneSearchResult?GENE_LIST_TYPE=1&type=504&GENOME_LIST=n315G1&CLASS_ID=33.07&WITH_GENE_MAP=1) |
| SA0498 | *rplL* | 50S ribosomal protein L7/L12 | -5.5 | 0.00 | [Protein synthesis](http://www.bio.nite.go.jp/dogan/GeneSearchResult?GENE_LIST_TYPE=1&type=504&GENOME_LIST=n315G1&CLASS_ID=33.07&WITH_GENE_MAP=1) |
| SA1503 | *rpmI* | 50S ribosomal protein L35 | -4.8 | 0.00 | [Protein synthesis](http://www.bio.nite.go.jp/dogan/GeneSearchResult?GENE_LIST_TYPE=1&type=504&GENOME_LIST=n315G1&CLASS_ID=33.07&WITH_GENE_MAP=1) |
| SAS093 | *rpmH* | 50S ribosomal protein L34 | -4.7 | 0.00 | [Protein synthesis](http://www.bio.nite.go.jp/dogan/GeneSearchResult?GENE_LIST_TYPE=1&type=504&GENOME_LIST=n315G1&CLASS_ID=33.07&WITH_GENE_MAP=1) |
| SA1504 | *infC* | Translation initiation factor IF-3 infC | -4.2 | 0.00 | [Protein synthesis](http://www.bio.nite.go.jp/dogan/GeneSearchResult?GENE_LIST_TYPE=1&type=504&GENOME_LIST=n315G1&CLASS_ID=33.07&WITH_GENE_MAP=1) |
| SA1920 | *prfA* | Peptide chain release factor 1 | -3.7 | 0.00 | [Protein synthesis](http://www.bio.nite.go.jp/dogan/GeneSearchResult?GENE_LIST_TYPE=1&type=504&GENOME_LIST=n315G1&CLASS_ID=33.07&WITH_GENE_MAP=1) |
| SA0986 | *pheT* | Phe-tRNA synthetase beta chain | -3.6 | 0.00 | [Protein synthesis](http://www.bio.nite.go.jp/dogan/GeneSearchResult?GENE_LIST_TYPE=1&type=504&GENOME_LIST=n315G1&CLASS_ID=33.07&WITH_GENE_MAP=1) |
| SA1081 | *rpsP* | 30S ribosomal protein S16 | -3.4 | 0.00 | [Protein synthesis](http://www.bio.nite.go.jp/dogan/GeneSearchResult?GENE_LIST_TYPE=1&type=504&GENOME_LIST=n315G1&CLASS_ID=33.07&WITH_GENE_MAP=1) |
| SA1414 | *rpsT* | 30S ribosomal protein S20 (BS20) | -3.4 | 0.00 | [Protein synthesis](http://www.bio.nite.go.jp/dogan/GeneSearchResult?GENE_LIST_TYPE=1&type=504&GENOME_LIST=n315G1&CLASS_ID=33.07&WITH_GENE_MAP=1) |
| SAS052 | *rpsD* | 30S ribosomal protein S4 | -3.4 | 0.00 | [Protein synthesis](http://www.bio.nite.go.jp/dogan/GeneSearchResult?GENE_LIST_TYPE=1&type=504&GENOME_LIST=n315G1&CLASS_ID=33.07&WITH_GENE_MAP=1) |
| SA1036 | *ileS* | Ile-tRNA synthetase | -3.0 | 0.00 | [Protein synthesis](http://www.bio.nite.go.jp/dogan/GeneSearchResult?GENE_LIST_TYPE=1&type=504&GENOME_LIST=n315G1&CLASS_ID=33.07&WITH_GENE_MAP=1) |
| SA0985 | *pheS* | Phe-tRNA synthetase alpha chain | -3.0 | 0.00 | [Protein synthesis](http://www.bio.nite.go.jp/dogan/GeneSearchResult?GENE_LIST_TYPE=1&type=504&GENOME_LIST=n315G1&CLASS_ID=33.07&WITH_GENE_MAP=1) |
| SA2039 | *rpmC* | 50S ribosomal protein L29 | -2.9 | 0.00 | [Protein synthesis](http://www.bio.nite.go.jp/dogan/GeneSearchResult?GENE_LIST_TYPE=1&type=504&GENOME_LIST=n315G1&CLASS_ID=33.07&WITH_GENE_MAP=1) |
| SA0486 | *gltX* | Glutamyl-tRNA synthetase | -2.9 | 0.00 | [Protein synthesis](http://www.bio.nite.go.jp/dogan/GeneSearchResult?GENE_LIST_TYPE=1&type=504&GENOME_LIST=n315G1&CLASS_ID=33.07&WITH_GENE_MAP=1) |
| SA2041 | *rpsC* | 30S ribosomal protein S3 | -2.8 | 0.00 | [Protein synthesis](http://www.bio.nite.go.jp/dogan/GeneSearchResult?GENE_LIST_TYPE=1&type=504&GENOME_LIST=n315G1&CLASS_ID=33.07&WITH_GENE_MAP=1) |
| SA0855 | *trpS* | Tryptophanyl-tRNA synthetase | -2.8 | 0.00 | [Protein synthesis](http://www.bio.nite.go.jp/dogan/GeneSearchResult?GENE_LIST_TYPE=1&type=504&GENOME_LIST=n315G1&CLASS_ID=33.07&WITH_GENE_MAP=1) |
| SA1456 | *aspS* | aspartyl-tRNA synthetase | -2.7 | 0.00 | [Protein synthesis](http://www.bio.nite.go.jp/dogan/GeneSearchResult?GENE_LIST_TYPE=1&type=504&GENOME_LIST=n315G1&CLASS_ID=33.07&WITH_GENE_MAP=1) |
| SA0497 | *rplJ* | 50S ribosomal protein L10 (BL5) | -2.6 | 0.07 | [Protein synthesis](http://www.bio.nite.go.jp/dogan/GeneSearchResult?GENE_LIST_TYPE=1&type=504&GENOME_LIST=n315G1&CLASS_ID=33.07&WITH_GENE_MAP=1) |
| SA1446 | *alaS* | Alanyl-tRNA synthetase | -2.6 | 0.07 | [Protein synthesis](http://www.bio.nite.go.jp/dogan/GeneSearchResult?GENE_LIST_TYPE=1&type=504&GENOME_LIST=n315G1&CLASS_ID=33.07&WITH_GENE_MAP=1) |
| SA1717 |  | Glutamyl-tRNAGln amidotransferase subunit C | -2.6 | 0.00 | [Protein synthesis](http://www.bio.nite.go.jp/dogan/GeneSearchResult?GENE_LIST_TYPE=1&type=504&GENOME_LIST=n315G1&CLASS_ID=33.07&WITH_GENE_MAP=1) |
| SA1457 | *hisS* | Histidyl-tRNA synthetase | -2.6 | 0.00 | [Protein synthesis](http://www.bio.nite.go.jp/dogan/GeneSearchResult?GENE_LIST_TYPE=1&type=504&GENOME_LIST=n315G1&CLASS_ID=33.07&WITH_GENE_MAP=1) |
| SAS079 | *rpsN* | 30S ribosomal protein S14 | -2.5 | 0.07 | [Protein synthesis](http://www.bio.nite.go.jp/dogan/GeneSearchResult?GENE_LIST_TYPE=1&type=504&GENOME_LIST=n315G1&CLASS_ID=33.07&WITH_GENE_MAP=1) |
| SA2043 | *rpsS* | 30S ribosomal protein S19 | -2.5 | 0.07 | [Protein synthesis](http://www.bio.nite.go.jp/dogan/GeneSearchResult?GENE_LIST_TYPE=1&type=504&GENOME_LIST=n315G1&CLASS_ID=33.07&WITH_GENE_MAP=1) |
| SA2034 | *rpsH* | 30S ribosomal protein S8 (BS8) | -2.5 | 0.07 | [Protein synthesis](http://www.bio.nite.go.jp/dogan/GeneSearchResult?GENE_LIST_TYPE=1&type=504&GENOME_LIST=n315G1&CLASS_ID=33.07&WITH_GENE_MAP=1) |
| SA2016 | *rpsI* | 30S ribosomal protein S9 (BS10) | -2.5 | 0.07 | [Protein synthesis](http://www.bio.nite.go.jp/dogan/GeneSearchResult?GENE_LIST_TYPE=1&type=504&GENOME_LIST=n315G1&CLASS_ID=33.07&WITH_GENE_MAP=1) |
| SA0496 | *rplA* | 50S ribosomal protein L1 (BL1) | -2.5 | 0.07 | [Protein synthesis](http://www.bio.nite.go.jp/dogan/GeneSearchResult?GENE_LIST_TYPE=1&type=504&GENOME_LIST=n315G1&CLASS_ID=33.07&WITH_GENE_MAP=1) |
| SA0959 |  | GTP-binding elongation factor homolog | -2.5 | 0.07 | [Protein synthesis](http://www.bio.nite.go.jp/dogan/GeneSearchResult?GENE_LIST_TYPE=1&type=504&GENOME_LIST=n315G1&CLASS_ID=33.07&WITH_GENE_MAP=1) |
| SA2044 | *rplB* | 50S ribosomal protein L2 (BL2) | -2.4 | 0.07 | [Protein synthesis](http://www.bio.nite.go.jp/dogan/GeneSearchResult?GENE_LIST_TYPE=1&type=504&GENOME_LIST=n315G1&CLASS_ID=33.07&WITH_GENE_MAP=1) |
| SA0564 | *argS* | Arginyl-tRNA synthetase | -2.4 | 0.07 | [Protein synthesis](http://www.bio.nite.go.jp/dogan/GeneSearchResult?GENE_LIST_TYPE=1&type=504&GENOME_LIST=n315G1&CLASS_ID=33.07&WITH_GENE_MAP=1) |
| SA2045 | *rplW* | 50S ribosomal protein L23 | -2.3 | 0.11 | [Protein synthesis](http://www.bio.nite.go.jp/dogan/GeneSearchResult?GENE_LIST_TYPE=1&type=504&GENOME_LIST=n315G1&CLASS_ID=33.07&WITH_GENE_MAP=1) |
| SA2046 | *rplD* | 50S ribosomal protein L4 | -2.3 | 0.11 | [Protein synthesis](http://www.bio.nite.go.jp/dogan/GeneSearchResult?GENE_LIST_TYPE=1&type=504&GENOME_LIST=n315G1&CLASS_ID=33.07&WITH_GENE_MAP=1) |
| SA2048 | *rpsJ* | 30S ribosomal protein S10 | -2.2 | 0.13 | [Protein synthesis](http://www.bio.nite.go.jp/dogan/GeneSearchResult?GENE_LIST_TYPE=1&type=504&GENOME_LIST=n315G1&CLASS_ID=33.07&WITH_GENE_MAP=1) |
| SA1099 | *rpsB* | 30S ribosomal protein S2 | -2.2 | 0.11 | [Protein synthesis](http://www.bio.nite.go.jp/dogan/GeneSearchResult?GENE_LIST_TYPE=1&type=504&GENOME_LIST=n315G1&CLASS_ID=33.07&WITH_GENE_MAP=1) |
| SA0352 | *rpsF* | 30S ribosomal protein S6 | -2.2 | 0.13 | [Protein synthesis](http://www.bio.nite.go.jp/dogan/GeneSearchResult?GENE_LIST_TYPE=1&type=504&GENOME_LIST=n315G1&CLASS_ID=33.07&WITH_GENE_MAP=1) |
| SA2040 | *rplP* | 50S ribosomal protein L16 | -2.2 | 0.13 | [Protein synthesis](http://www.bio.nite.go.jp/dogan/GeneSearchResult?GENE_LIST_TYPE=1&type=504&GENOME_LIST=n315G1&CLASS_ID=33.07&WITH_GENE_MAP=1) |
| SA1084 | *rplS* | 50S ribosomal protein L19 | -2.2 | 0.20 | [Protein synthesis](http://www.bio.nite.go.jp/dogan/GeneSearchResult?GENE_LIST_TYPE=1&type=504&GENOME_LIST=n315G1&CLASS_ID=33.07&WITH_GENE_MAP=1) |
| SA2047 | *rplC* | 50S ribosomal protein L3 (BL3) | -2.2 | 0.13 | [Protein synthesis](http://www.bio.nite.go.jp/dogan/GeneSearchResult?GENE_LIST_TYPE=1&type=504&GENOME_LIST=n315G1&CLASS_ID=33.07&WITH_GENE_MAP=1) |
| SA1059 |  | Methionyl-tRNA formyltransferase | -2.2 | 0.11 | [Protein synthesis](http://www.bio.nite.go.jp/dogan/GeneSearchResult?GENE_LIST_TYPE=1&type=504&GENOME_LIST=n315G1&CLASS_ID=33.07&WITH_GENE_MAP=1) |
| SA0877 | *prfC* | Peptide chain release factor 3 | -2.2 | 0.13 | [Protein synthesis](http://www.bio.nite.go.jp/dogan/GeneSearchResult?GENE_LIST_TYPE=1&type=504&GENOME_LIST=n315G1&CLASS_ID=33.07&WITH_GENE_MAP=1) |
| SA2037 | *rplN* | 50S ribosomal protein L14 | -2.1 | 0.19 | [Protein synthesis](http://www.bio.nite.go.jp/dogan/GeneSearchResult?GENE_LIST_TYPE=1&type=504&GENOME_LIST=n315G1&CLASS_ID=33.07&WITH_GENE_MAP=1) |
| SA1716 |  | Glutamyl-tRNAGln amidotransferase subunit A | -2.1 | 0.13 | [Protein synthesis](http://www.bio.nite.go.jp/dogan/GeneSearchResult?GENE_LIST_TYPE=1&type=504&GENOME_LIST=n315G1&CLASS_ID=33.07&WITH_GENE_MAP=1) |
| SA1579 | *leuS* | Leucyl-tRNA synthetase | -2.1 | 0.13 | [Protein synthesis](http://www.bio.nite.go.jp/dogan/GeneSearchResult?GENE_LIST_TYPE=1&type=504&GENOME_LIST=n315G1&CLASS_ID=33.07&WITH_GENE_MAP=1) |
| SA0877 | *prfC* | Peptide chain release factor 3 | -2.1 | 0.13 | [Protein synthesis](http://www.bio.nite.go.jp/dogan/GeneSearchResult?GENE_LIST_TYPE=1&type=504&GENOME_LIST=n315G1&CLASS_ID=33.07&WITH_GENE_MAP=1) |
| SA1359 |  | Translation elongation factor EF-P | -2.1 | 0.20 | [Protein synthesis](http://www.bio.nite.go.jp/dogan/GeneSearchResult?GENE_LIST_TYPE=1&type=504&GENOME_LIST=n315G1&CLASS_ID=33.07&WITH_GENE_MAP=1) |
| SAS042 | *rpmG* | 50S ribosomal protein L33 | 3.9 | 0.00 | [Protein synthesis](http://www.bio.nite.go.jp/dogan/GeneSearchResult?GENE_LIST_TYPE=1&type=504&GENOME_LIST=n315G1&CLASS_ID=33.07&WITH_GENE_MAP=1) |
| SA1082 | *rimM* | Probable 16S rRNA processing protein | -4.7 | 0.00 | [RNA modification](http://www.bio.nite.go.jp/dogan/GeneSearchResult?GENE_LIST_TYPE=1&type=504&GENOME_LIST=n315G1&CLASS_ID=33.06&WITH_GENE_MAP=1) |
| SA1083 | *trmD* | tRNA (guanine-N1)-mehtyltransferase | -4.1 | 0.00 | [RNA modification](http://www.bio.nite.go.jp/dogan/GeneSearchResult?GENE_LIST_TYPE=1&type=504&GENOME_LIST=n315G1&CLASS_ID=33.06&WITH_GENE_MAP=1) |
| SA1387 |  | Hypothetical protein, similar to ATP-dependent RNA helicase | -2.6 | 0.00 | [RNA modification](http://www.bio.nite.go.jp/dogan/GeneSearchResult?GENE_LIST_TYPE=1&type=504&GENOME_LIST=n315G1&CLASS_ID=33.06&WITH_GENE_MAP=1) |
| SA1114 | *truB* | tRNA pseudouridine 5S synthase | -2.3 | 0.2 | [RNA modification](http://www.bio.nite.go.jp/dogan/GeneSearchResult?GENE_LIST_TYPE=1&type=504&GENOME_LIST=n315G1&CLASS_ID=33.06&WITH_GENE_MAP=1) |
| SA1466 | *queA* | S-adenosylmethionine tRNA ribosyltransferase | -2.2 | 0.11 | [RNA modification](http://www.bio.nite.go.jp/dogan/GeneSearchResult?GENE_LIST_TYPE=1&type=504&GENOME_LIST=n315G1&CLASS_ID=33.06&WITH_GENE_MAP=1) |
| SA1060 |  | Hypothetical protein, similar to RNA-binding Sun protein | -2.2 | 0.13 | [RNA modification](http://www.bio.nite.go.jp/dogan/GeneSearchResult?GENE_LIST_TYPE=1&type=504&GENOME_LIST=n315G1&CLASS_ID=33.06&WITH_GENE_MAP=1) |
| SA2502 | *rnpA* | Ribonuclease P protein component | -2.1 | 0.11 | [RNA modification](http://www.bio.nite.go.jp/dogan/GeneSearchResult?GENE_LIST_TYPE=1&type=504&GENOME_LIST=n315G1&CLASS_ID=33.06&WITH_GENE_MAP=1) |
| SA1041 | *pyrR* | Pyrimidine operon repressor chainA | -6.1 | 0.00 | [RNA synthesis](http://www.bio.nite.go.jp/dogan/GeneSearchResult?GENE_LIST_TYPE=1&type=504&GENOME_LIST=n315G1&CLASS_ID=33.05&WITH_GENE_MAP=1) |
| SA2092 |  | Hypothetical protein, similar to transcription regulator | -4.7 | 0.00 | [RNA synthesis](http://www.bio.nite.go.jp/dogan/GeneSearchResult?GENE_LIST_TYPE=1&type=504&GENOME_LIST=n315G1&CLASS_ID=33.05&WITH_GENE_MAP=1) |
| SA2060 |  | Hypothetical protein, similar to transcription regulator MarR family | -4.1 | 0.00 | [RNA synthesis](http://www.bio.nite.go.jp/dogan/GeneSearchResult?GENE_LIST_TYPE=1&type=504&GENOME_LIST=n315G1&CLASS_ID=33.05&WITH_GENE_MAP=1) |
| SA2089 | *sarR* | Staphylococcal accessory regulator A homolog | -4.0 | 0.00 | [RNA synthesis](http://www.bio.nite.go.jp/dogan/GeneSearchResult?GENE_LIST_TYPE=1&type=504&GENOME_LIST=n315G1&CLASS_ID=33.05&WITH_GENE_MAP=1) |
| SA0251 | *lytR* | Two-component response regulator | -3.9 | 0.00 | [RNA synthesis](http://www.bio.nite.go.jp/dogan/GeneSearchResult?GENE_LIST_TYPE=1&type=504&GENOME_LIST=n315G1&CLASS_ID=33.05&WITH_GENE_MAP=1) |
| SA1872 | *rsbU* | SigmaB regulation protein RsbU | -3.0 | 0.00 | [RNA synthesis](http://www.bio.nite.go.jp/dogan/GeneSearchResult?GENE_LIST_TYPE=1&type=504&GENOME_LIST=n315G1&CLASS_ID=33.05&WITH_GENE_MAP=1) |
| SA1871 | *rsbV* | Anti-sigmaB factor antagonist | -3.0 | 0.00 | [RNA synthesis](http://www.bio.nite.go.jp/dogan/GeneSearchResult?GENE_LIST_TYPE=1&type=504&GENOME_LIST=n315G1&CLASS_ID=33.05&WITH_GENE_MAP=1) |
| SA2115 |  | Hypothetical protein, similar to transcriptional regulator | -3.0 | 0.00 | [RNA synthesis](http://www.bio.nite.go.jp/dogan/GeneSearchResult?GENE_LIST_TYPE=1&type=504&GENOME_LIST=n315G1&CLASS_ID=33.05&WITH_GENE_MAP=1) |
| SA2223 |  | Hypothetical protein, similar to transcriptional activator | -2.9 | 0.00 | [RNA synthesis](http://www.bio.nite.go.jp/dogan/GeneSearchResult?GENE_LIST_TYPE=1&type=504&GENOME_LIST=n315G1&CLASS_ID=33.05&WITH_GENE_MAP=1) |
| SA1139 | *glpP* | Glycerol uptake operon antiterminator regulatory protein | -2.7 | 0.07 | [RNA synthesis](http://www.bio.nite.go.jp/dogan/GeneSearchResult?GENE_LIST_TYPE=1&type=504&GENOME_LIST=n315G1&CLASS_ID=33.05&WITH_GENE_MAP=1) |
| SA1870 | *rsbW* | Anti-sigmaB factor | -2.5 | 0.07 | [RNA synthesis](http://www.bio.nite.go.jp/dogan/GeneSearchResult?GENE_LIST_TYPE=1&type=504&GENOME_LIST=n315G1&CLASS_ID=33.05&WITH_GENE_MAP=1) |
| SA0641 | *mgrA* | Transcriptional regulator MgrA | -2.3 | 0.11 | [RNA synthesis](http://www.bio.nite.go.jp/dogan/GeneSearchResult?GENE_LIST_TYPE=1&type=504&GENOME_LIST=n315G1&CLASS_ID=33.05&WITH_GENE_MAP=1) |
| SA1872 | *rsbU* | SigmaB regulation protein RsbU | -3.0 | 0.07 | [RNA synthesis](http://www.bio.nite.go.jp/dogan/GeneSearchResult?GENE_LIST_TYPE=1&type=504&GENOME_LIST=n315G1&CLASS_ID=33.05&WITH_GENE_MAP=1) |
| SA1869 | *sigB* | Sigma factor B | -2.3 | 0.07 | [RNA synthesis](http://www.bio.nite.go.jp/dogan/GeneSearchResult?GENE_LIST_TYPE=1&type=504&GENOME_LIST=n315G1&CLASS_ID=33.05&WITH_GENE_MAP=1) |
| SA1844 | *agrA* | Accessory gene regulator A | -2.2 | 0.19 | [RNA synthesis](http://www.bio.nite.go.jp/dogan/GeneSearchResult?GENE_LIST_TYPE=1&type=504&GENOME_LIST=n315G1&CLASS_ID=33.05&WITH_GENE_MAP=1) |
| SA2174 |  | Hypothetical protein, similar to transcriptional regulator | -2.2 | 0.13 | [RNA synthesis](http://www.bio.nite.go.jp/dogan/GeneSearchResult?GENE_LIST_TYPE=1&type=504&GENOME_LIST=n315G1&CLASS_ID=33.05&WITH_GENE_MAP=1) |
| SA1676 |  | Hypothetical protein, similar to regulatory protein (pfoS/R) | -2.2 | 0.13 | [RNA synthesis](http://www.bio.nite.go.jp/dogan/GeneSearchResult?GENE_LIST_TYPE=1&type=504&GENOME_LIST=n315G1&CLASS_ID=33.05&WITH_GENE_MAP=1) |
| SA0108 | *sarS* | Staphylococcal accessory regulator A homologue | -2.0 | 0.19 | [RNA synthesis](http://www.bio.nite.go.jp/dogan/GeneSearchResult?GENE_LIST_TYPE=1&type=504&GENOME_LIST=n315G1&CLASS_ID=33.05&WITH_GENE_MAP=1) |
| SA2123 |  | Hypothetical protein, 15imilar to transcription regulator LysR family | -2.0 | 0.25 | [RNA synthesis](http://www.bio.nite.go.jp/dogan/GeneSearchResult?GENE_LIST_TYPE=1&type=504&GENOME_LIST=n315G1&CLASS_ID=33.05&WITH_GENE_MAP=1) |
| SA1256 | *msrB* | Methionine sulfoxide reductase B | 2.1 | 0.25 | RNA synthesis |
| SA2103 | *LytR* | Hypothetical protein, similar to lyt divergon expression attenuator LytR | 2.1 | 0.25 | [RNA synthesis](http://www.bio.nite.go.jp/dogan/GeneSearchResult?GENE_LIST_TYPE=1&type=504&GENOME_LIST=n315G1&CLASS_ID=33.05&WITH_GENE_MAP=1) |
| SA1329 | *fur* | Ferric uptake regulator homolog | 2.1 | 0.38 | [RNA synthesis](http://www.bio.nite.go.jp/dogan/GeneSearchResult?GENE_LIST_TYPE=1&type=504&GENOME_LIST=n315G1&CLASS_ID=33.05&WITH_GENE_MAP=1) |
| SA1998 | *lacR* | Lactose phosphotransferase system repressor | 2.1 | 0.38 | [RNA synthesis](http://www.bio.nite.go.jp/dogan/GeneSearchResult?GENE_LIST_TYPE=1&type=504&GENOME_LIST=n315G1&CLASS_ID=33.05&WITH_GENE_MAP=1) |
| SA1174 | *lexA* | SOS regulatory LexA protein | 2.1 | 0.40 | [RNA synthesis](http://www.bio.nite.go.jp/dogan/GeneSearchResult?GENE_LIST_TYPE=1&type=504&GENOME_LIST=n315G1&CLASS_ID=33.05&WITH_GENE_MAP=1) |
| SA2308 |  | Hypothetical protein, similar to transcription regulator MarR family | 2.1 | 0.38 | [RNA synthesis](http://www.bio.nite.go.jp/dogan/GeneSearchResult?GENE_LIST_TYPE=1&type=504&GENOME_LIST=n315G1&CLASS_ID=33.05&WITH_GENE_MAP=1) |
| SA0142 |  | Hypothetical protein, similar to DNA-binding protein | 2.1 | 0.20 | [RNA synthesis](http://www.bio.nite.go.jp/dogan/GeneSearchResult?GENE_LIST_TYPE=1&type=504&GENOME_LIST=n315G1&CLASS_ID=33.05&WITH_GENE_MAP=1) |
| SA2379 |  | Hypothetical protein, similar to transcriptional regulator tetR-family | 2.2 | 0.19 | [RNA synthesis](http://www.bio.nite.go.jp/dogan/GeneSearchResult?GENE_LIST_TYPE=1&type=504&GENOME_LIST=n315G1&CLASS_ID=33.05&WITH_GENE_MAP=1) |
| SA0142 |  | Hypothetical protein, similar to DNA-binding protein | 2.2 | 0.40 | [RNA synthesis](http://www.bio.nite.go.jp/dogan/GeneSearchResult?GENE_LIST_TYPE=1&type=504&GENOME_LIST=n315G1&CLASS_ID=33.05&WITH_GENE_MAP=1) |
| SA2108 |  | Hypothetical protein, imilar to transcription regulator, RpiR family | 2.2 | 0.19 | [RNA synthesis](http://www.bio.nite.go.jp/dogan/GeneSearchResult?GENE_LIST_TYPE=1&type=504&GENOME_LIST=n315G1&CLASS_ID=33.05&WITH_GENE_MAP=1) |
| SA2105 |  | conserved hypothetical protein | 2.2 | 0.25 | [RNA synthesis](http://www.bio.nite.go.jp/dogan/GeneSearchResult?GENE_LIST_TYPE=1&type=504&GENOME_LIST=n315G1&CLASS_ID=33.05&WITH_GENE_MAP=1) |
| SA0261 |  | Hypothetical protein, similar to rbs operon repressor RbsR | 2.3 | 0.13 | [RNA synthesis](http://www.bio.nite.go.jp/dogan/GeneSearchResult?GENE_LIST_TYPE=1&type=504&GENOME_LIST=n315G1&CLASS_ID=33.05&WITH_GENE_MAP=1) |
| SA1678 | *fur* | Transcription regulator Fur family homolog | 2.5 | 0.11 | [RNA synthesis](http://www.bio.nite.go.jp/dogan/GeneSearchResult?GENE_LIST_TYPE=1&type=504&GENOME_LIST=n315G1&CLASS_ID=33.05&WITH_GENE_MAP=1) |
| SA0590 |  | Hypothetical protein, similar to iron dependent repressor | 2.5 | 0.06 | [RNA synthesis](http://www.bio.nite.go.jp/dogan/GeneSearchResult?GENE_LIST_TYPE=1&type=504&GENOME_LIST=n315G1&CLASS_ID=33.05&WITH_GENE_MAP=1) |
| SA1411 | *hrcA* | Heat-inducible transcriptional repressor | 2.8 | 0.06 | [RNA synthesis](http://www.bio.nite.go.jp/dogan/GeneSearchResult?GENE_LIST_TYPE=1&type=504&GENOME_LIST=n315G1&CLASS_ID=33.05&WITH_GENE_MAP=1) |
| SA1191 | *glcT* | Transcription antiterminator | 3.0 | 0.06 | [RNA synthesis](http://www.bio.nite.go.jp/dogan/GeneSearchResult?GENE_LIST_TYPE=1&type=504&GENOME_LIST=n315G1&CLASS_ID=33.05&WITH_GENE_MAP=1) |
| SA0627 |  | Hypothetical protein, similar to LysR family transcriptional regulator | 3.0 | 0.00 | [RNA synthesis](http://www.bio.nite.go.jp/dogan/GeneSearchResult?GENE_LIST_TYPE=1&type=504&GENOME_LIST=n315G1&CLASS_ID=33.05&WITH_GENE_MAP=1) |
| SA2062 | *sarV* | Staphylococcal accessory regulator A homolog | 3.2 | 0.00 | RNA synthesis |
| SA1701 | *vraS* | Two-component sensor histidine kinase | 3.6 | 0.00 | RNA synthesis |
| SA2458 | *icaR* | Ica operon transcriptional regulator IcaR | 3.6 | 0.00 | [RNA synthesis](http://www.bio.nite.go.jp/dogan/GeneSearchResult?GENE_LIST_TYPE=1&type=504&GENOME_LIST=n315G1&CLASS_ID=33.05&WITH_GENE_MAP=1) |
| SA1999 |  | Hypothetical protein, similar to regulatory protein, SIR2 family | 3.7 | 0.00 | [RNA synthesis](http://www.bio.nite.go.jp/dogan/GeneSearchResult?GENE_LIST_TYPE=1&type=504&GENOME_LIST=n315G1&CLASS_ID=33.05&WITH_GENE_MAP=1) |
| SA2364 |  | Hypothetical protein, similar to transcription regulator acrR | 4.1 | 0.00 | [RNA synthesis](http://www.bio.nite.go.jp/dogan/GeneSearchResult?GENE_LIST_TYPE=1&type=504&GENOME_LIST=n315G1&CLASS_ID=33.05&WITH_GENE_MAP=1) |
| SA1700 | *vraR* | Two-component response regulator | 4.2 | 0.00 | [RNA synthesis](http://www.bio.nite.go.jp/dogan/GeneSearchResult?GENE_LIST_TYPE=1&type=504&GENOME_LIST=n315G1&CLASS_ID=33.05&WITH_GENE_MAP=1) |
| SA1947 | *czrA* | repressor protein | 4.4 | 0.00 | [RNA synthesis](http://www.bio.nite.go.jp/dogan/GeneSearchResult?GENE_LIST_TYPE=1&type=504&GENOME_LIST=n315G1&CLASS_ID=33.05&WITH_GENE_MAP=1) |
| SA2002 |  | Hypothetical protein, similar to transcription regulator MerR family | 4.7 | 0.00 | [RNA synthesis](http://www.bio.nite.go.jp/dogan/GeneSearchResult?GENE_LIST_TYPE=1&type=504&GENOME_LIST=n315G1&CLASS_ID=33.05&WITH_GENE_MAP=1) |
| SA0480 | *ctsR* | Transcription repressor of class III stress genes homologue | 5.6 | 0.00 | [RNA synthesis](http://www.bio.nite.go.jp/dogan/GeneSearchResult?GENE_LIST_TYPE=1&type=504&GENOME_LIST=n315G1&CLASS_ID=33.05&WITH_GENE_MAP=1) |
| SA2295 | *gntR* | Gluconate operon transcriptional repressor | 6.8 | 0.00 | [RNA synthesis](http://www.bio.nite.go.jp/dogan/GeneSearchResult?GENE_LIST_TYPE=1&type=504&GENOME_LIST=n315G1&CLASS_ID=33.05&WITH_GENE_MAP=1) |
| SA2296 |  | Hypothetical protein, etracy to transcriptional regulator, MerR family | 8.4 | 0.00 | [RNA synthesis](http://www.bio.nite.go.jp/dogan/GeneSearchResult?GENE_LIST_TYPE=1&type=504&GENOME_LIST=n315G1&CLASS_ID=33.05&WITH_GENE_MAP=1) |
| SA0250 | *lytS* | Two-component sensor histidine kinase | -1.9 | 0.25 | Sensors (signal transduction) |
| SA0269 |  | Hypothetical protein | -23.1 | 0.00 | similar to unknown proteins |
| SA2332 |  | Hypothetical protein, similar to secretory antigen precursor SsaA | -14.2 | 0.00 | similar to unknown proteins |
| SA0274 |  | Conserved hypothetical protein | -14.1 | 0.00 | Similar to unknown proteins |
| SA0275 |  | Conserved hypothetical protein | -11.2 | 0.00 | similar to unknown proteins |
| SA0213 |  | Conserved hypothetical protein | -9.2 | 0.00 | similar to unknown proteins |
| SA0976 | *isdB* | Conserved hypothetical protein | -7.4 | 0.00 | similar to unknown proteins |
| SA1849 |  | Conserved hypothetical protein | -6.3 | 0.00 | similar to unknown proteins |
| SA1265 |  | Conserved hypothetical protein | -6.1 | 0.00 | similar to unknown proteins |
| SA1850 |  | Conserved hypothetical protein | -6.0 | 0.00 | similar to unknown proteins |
| SA0165 |  | Hypothetical protein, similar to alpha-helical coiled-coil protein SrpF | -5.4 | 0.00 | similar to unknown proteins |
| SA0890 |  | Conserved hypothetical protein | -5.2 | 0.00 | similar to unknown proteins |
| SA0949 |  | Conserved hypothetical protein | -4.9 | 0.00 | similar to unknown proteins |
| SA0308 |  | Conserved hypothetical protein | -4.3 | 0.00 | similar to unknown proteins |
| SA2133 |  | Conserved hypothetical protein | -4.0 | 0.00 | similar to unknown proteins |
| SA2133 |  | Conserved hypothetical protein | -4.0 | 0.00 | similar to unknown proteins |
| SA1275 |  | Conserved hypothetical protein | -3.8 | 0.00 | similar to unknown proteins |
| SA0478 |  | Conserved hypothetical protein | -3.7 | 0.00 | similar to unknown proteins |
| SA1295 |  | Conserved hypothetical protein | -3.7 | 0.00 | similar to unknown proteins |
| SA0739 |  | Conserved hypothetical protein | -3.6 | 0.00 | similar to unknown proteins |
| SA1705 |  | Conserved hypothetical protein | -3.5 | 0.00 | similar to unknown proteins |
| SA2133 |  | Conserved hypothetical protein | -3.5 | 0.00 | similar to unknown proteins |
| SA2407 |  | Conserved hypothetical protein | -3.4 | 0.00 | similar to unknown proteins |
| SA0518 |  | Conserved hypothetical protein | -3.3 | 0.00 | similar to unknown proteins |
| SA0983 | *isdG* | Conserved hypothetical protein | -3.3 | 0.00 | similar to unknown proteins |
| SA0966 |  | Conserved hypothetical protein | -3.3 | 0.00 | similar to unknown proteins |
| SA2448 |  | Conserved hypothetical protein | -3.2 | 0.00 | similar to unknown proteins |
| SA0517 |  | Conserved hypothetical protein | -3.2 | 0.00 | similar to unknown proteins |
| SA1294 |  | Conserved hypothetical protein | -3.1 | 0.00 | similar to unknown proteins |
| SA0840 |  | Conserved hypothetical protein | -3.1 | 0.00 | similar to unknown proteins |
| SA0499 |  | Conserved hypothetical protein | -3.0 | 0.00 | similar to unknown proteins |
| SA0906 |  | Conserved hypothetical protein | -3.0 | 0.00 | similar to unknown proteins |
| SAS001 |  | Conserved hypothetical protein | -2.9 | 0.00 | similar to unknown proteins |
| SA0982 | *srtB* | NPQTN specific sortase B | -2.9 | 0.00 | similar to unknown proteins |
| SA0509 |  | Conserved hypothetical protein | -2.9 | 0.00 | similar to unknown proteins |
| SA2163 |  | Hypothetical protein | -2.8 | 0.00 | similar to unknown proteins |
| SA1867 |  | Conserved hypothetical protein | -2.8 | 0.07 | similar to unknown proteins |
| SA0089 |  | Hypothetical protein, similar to DNA helicase | -2.8 | 0.00 | similar to unknown proteins |
| SA1536 |  | Conserved hypothetical protein | -2.8 | 0.00 | similar to unknown proteins |
| SA0452 | *veg* | VEG protein homologue | -2.8 | 0.00 | similar to unknown proteins |
| SA0023 | *orfX* | Conserved hypothetical protein orfX | -2.8 | 0.00 | [Similar to unknown proteins](http://www.bio.nite.go.jp/dogan/GeneSearchResult?GENE_LIST_TYPE=1&type=504&GENOME_LIST=n315G1&CLASS_ID=35.01&WITH_GENE_MAP=1) |
| SA1062 |  | Conserved hypothetical protein | -2.7 | 0.00 | similar to unknown proteins |
| SA1426 |  | Conserved hypothetical protein | -2.7 | 0.00 | similar to unknown proteins |
| SA0556 |  | Conserved hypothetical protein | -2.6 | 0.07 | similar to unknown proteins |
| SA2193 |  | Conserved hypothetical protein | -2.6 | 0.07 | similar to unknown proteins |
| SA1425 |  | Conserved hypothetical protein | -2.6 | 0.07 | similar to unknown proteins |
| SA1723 |  | Conserved hypothetical protein | -2.5 | 0.07 | similar to unknown proteins |
| SA1422 |  | Conserved hypothetical protein | -2.5 | 0.07 | similar to unknown proteins |
| SA0947 |  | Conserved hypothetical protein | -2.5 | 0.07 | similar to unknown proteins |
| SA2276 |  | Conserved hypothetical protein | -2.5 | 0.07 | similar to unknown proteins |
| SA1578 |  | Conserved hypothetical protein | -2.4 | 0.07 | similar to unknown proteins |
| SA0341 |  | Hypothetical protein, similar to low temperature requirement A protein | -2.4 | 0.07 | similar to unknown proteins |
| SA0789 |  | Conserved hypothetical protein | -2.4 | 0.07 | similar to unknown proteins |
| SA1296 |  | Conserved hypothetical protein | -2.4 | 0.07 | similar to unknown proteins |
| SA1040 |  | Conserved hypothetical protein | -2.4 | 0.07 | similar to unknown proteins |
| SA2106 |  | Hypothetical protein, similar to protein of pXO2-46 | -2.4 | 0.07 | similar to unknown proteins |
| SA0683 |  | Conserved hypothetical protein | -2.3 | 0.11 | similar to unknown proteins |
| SA1061 |  | Conserved hypothetical protein | -2.3 | 0.07 | similar to unknown proteins |
| SA0979 | *isdD* | Conserved hypothetical protein | -2.3 | 0.11 | similar to unknown proteins |
| SA1240 |  | Conserved hypothetical protein | -2.3 | 0.07 | similar to unknown proteins |
| SA0694 |  | Conserved hypothetical protein | -2.3 | 0.07 | similar to unknown proteins |
| SA0814 |  | Conserved hypothetical protein | -2.3 | 0.07 | similar to unknown proteins |
| SA0609 |  | Conserved hypothetical protein | -2.2 | 0.19 | similar to unknown proteins |
| SA0301 |  | Conserved hypothetical protein | -2.2 | 0.13 | similar to unknown proteins |
| SA1576 |  | Conserved hypothetical protein | -2.2 | 0.13 | similar to unknown proteins |
| SA0126 |  | Hypothetical protein, similar to capsular polysaccharide synthesis protein 14H | -2.2 | 0.11 | similar to unknown proteins |
| SA0307 |  | Conserved hypothetical protein | -2.1 | 0.19 | similar to unknown proteins |
| SA1867 |  | Conserved hypothetical protein | -2.1 | 0.13 | similar to unknown proteins |
| SA0870 |  | Conserved hypothetical protein | -2.1 | 0.13 | similar to unknown proteins |
| SA2474 |  | Conserved hypothetical protein | -2.1 | 0.20 | similar to unknown proteins |
| SA1612 |  | Conserved hypothetical protein | -2.1 | 0.19 | similar to unknown proteins |
| SA2478 |  | Conserved hypothetical protein | -2.1 | 0.19 | similar to unknown proteins |
| SA1378 |  | Conserved hypothetical protein | -2.1 | 0.13 | similar to unknown proteins |
| SA1130 |  | Conserved hypothetical protein | -2.0 | 0.20 | similar to unknown proteins |
| SA0447 |  | Conserved hypothetical protein | -2.0 | 0.20 | similar to unknown proteins |
| SA0667 |  | Conserved hypothetical protein | -2.0 | 0.20 | similar to unknown proteins |
| SA0649 |  | Conserved hypothetical protein | -2.0 | 0.20 | similar to unknown proteins |
| SA2313 |  | Conserved hypothetical protein | -2.0 | 0.25 | similar to unknown proteins |
| SA1335 |  | Conserved hypothetical protein | -2.0 | 0.19 | similar to unknown proteins |
| SA0477 |  | Conserved hypothetical protein | -2.0 | 0.20 | similar to unknown proteins |
| SA1389 |  | Conserved hypothetical protein | -2.0 | 0.19 | similar to unknown proteins |
| SA0903 |  | Conserved hypothetical protein | 2.0 | 0.61 | similar to unknown proteins |
| SA1064 |  | Conserved hypothetical protein | 2.0 | 0.38 | similar to unknown proteins |
| SA2160 |  | Conserved hypothetical protein | 2.0 | 0.40 | similar to unknown proteins |
| SA0606 |  | Conserved hypothetical protein | 2.0 | 0.4 | similar to unknown proteins |
| SA1975 |  | Conserved hypothetical protein | 2.0 | 0.38 | similar to unknown proteins |
| SA1900 |  | Conserved hypothetical protein | 2.0 | 0.40 | similar to unknown proteins |
| SA2238 |  | Conserved hypothetical protein | 2.1 | 0.38 | similar to unknown proteins |
| SA1345 |  | Conserved hypothetical protein | 2.1 | 0.25 | similar to unknown proteins |
| SA0824 |  | Conserved hypothetical protein | 2.1 | 0.25 | similar to unknown proteins |
| SA1018 |  | Conserved hypothetical protein | 2.1 | 0.38 | similar to unknown proteins |
| SA0833 |  | Conserved hypothetical protein | 2.1 | 0.40 | similar to unknown proteins |
| SA0832 |  | Conserved hypothetical protein | 2.1 | 0.38 | similar to unknown proteins |
| SA0012 |  | Conserved hypothetical protein | 2.1 | 0.25 | [Similar to unknown proteins](http://www.bio.nite.go.jp/dogan/GeneSearchResult?GENE_LIST_TYPE=1&type=504&GENOME_LIST=n315G1&CLASS_ID=35.01&WITH_GENE_MAP=1) |
| SA2440 |  | Hypothetical protein | 2.1 | 0.40 | similar to unknown proteins |
| SA2225 |  | Conserved hypothetical protein | 2.2 | 0.25 | similar to unknown proteins |
| SA1293 |  | Conserved hypothetical protein | 2.2 | 0.25 | similar to unknown proteins |
| SA1167 |  | Conserved hypothetical protein | 2.2 | 0.20 | similar to unknown proteins |
| SA0968 |  | Conserved hypothetical protein | 2.2 | 0.2 | similar to unknown proteins |
| SA1327 |  | Conserved hypothetical protein | 2.2 | 0.25 | similar to unknown proteins |
| SA2360 |  | Conserved hypothetical protein | 2.2 | 0.25 | similar to unknown proteins |
| SA0314 |  | Conserved hypothetical protein | 2.2 | 0.20 | similar to unknown proteins |
| SA2439 |  | Conserved hypothetical protein | 2.2 | 0.20 | similar to unknown proteins |
| SA0720 |  | Conserved hypothetical protein | 2.2 | 0.25 | similar to unknown proteins |
| SA1154 |  | Conserved hypothetical protein | 2.2 | 0.38 | similar to unknown proteins |
| SA1680 |  | Conserved hypothetical protein | 2.2 | 0.38 | similar to unknown proteins |
| SA0967 |  | Conserved hypothetical protein | 2.3 | 0.19 | similar to unknown proteins |
| SA0327 |  | Conserved hypothetical protein | 2.3 | 0.19 | similar to unknown proteins |
| SA1037 |  | Conserved hypothetical protein | 2.4 | 0.19 | similar to unknown proteins |
| SA0326 |  | Conserved hypothetical protein | 2.4 | 0.13 | similar to unknown proteins |
| SA1532 |  | Conserved hypothetical protein | 2.4 | 0.19 | similar to unknown proteins |
| SA0893 |  | Conserved hypothetical protein | 2.5 | 0.13 | similar to unknown proteins |
| SA2128 |  | Conserved hypothetical protein | 2.5 | 0.13 | similar to unknown proteins |
| SA0722 |  | Conserved hypothetical protein | 2.5 | 0.06 | similar to unknown proteins |
| SA1393 |  | Conserved hypothetical protein | 2.5 | 0.11 | similar to unknown proteins |
| SA2220 |  | Conserved hypothetical protein | 2.5 | 0.06 | similar to unknown proteins |
| SA0607 |  | Conserved hypothetical protein | 2.5 | 0.19 | similar to unknown proteins |
| SA2196 |  | Conserved hypothetical protein | 2.5 | 0.11 | similar to unknown proteins |
| SA1841 |  | Hypothetical protein | 2.6 | 0.06 | similar to unknown proteins |
| SA2491 |  | Conserved hypothetical protein | 2.6 | 0.19 | similar to unknown proteins |
| SA1957 |  | Conserved hypothetical protein | 2.7 | 0.06 | similar to unknown proteins |
| SA0968 |  | Conserved hypothetical protein | 2.8 | 0.06 | similar to unknown proteins |
| SA2365 |  | Hypothetical protein, similar to short chain oxidoreductase | 2.8 | 0.06 | similar to unknown proteins |
| SA2403 |  | Conserved hypothetical protein | 2.8 | 0.06 | similar to unknown proteins |
| SA2378 |  | Conserved hypothetical protein | 2.8 | 0.06 | similar to unknown proteins |
| SA1453 |  | Conserved hypothetical protein | 2.8 | 0.06 | similar to unknown proteins |
| SA1176 |  | Conserved hypothetical protein | 2.8 | 0.11 | similar to unknown proteins |
| SA1568 |  | Conserved hypothetical protein | 2.8 | 0.06 | similar to unknown proteins |
| SA1292 |  | Conserved hypothetical protein | 2.9 | 0.00 | similar to unknown proteins |
| SA0621 |  | Conserved hypothetical protein | 2.9 | 0.00 | similar to unknown proteins |
| SA1316 |  | Conserved hypothetical protein | 2.9 | 0.06 | similar to unknown proteins |
| SA1712 |  | Conserved hypothetical protein | 2.9 | 0.00 | similar to unknown proteins |
| SA1281 |  | Conserved hypothetical protein | 3.0 | 0.00 | similar to unknown proteins |
| SA1254 |  | Conserved hypothetical protein | 3.0 | 0.00 | similar to unknown proteins |
| SA0860 |  | Conserved hypothetical protein | 3.0 | 0.00 | similar to unknown proteins |
| SA1236 |  | Conserved hypothetical protein | 3.0 | 0.00 | similar to unknown proteins |
| SA1682 |  | Conserved hypothetical protein | 3.0 | 0.00 | similar to unknown proteins |
| SA0626 |  | Conserved hypothetical protein | 3.1 | 0.00 | similar to unknown proteins |
| SA1453 |  | Conserved hypothetical protein | 3.2 | 0.00 | similar to unknown proteins |
| SA0862 |  | Conserved hypothetical protein | 3.3 | 0.00 | similar to unknown proteins |
| SA0863 |  | Conserved hypothetical protein | 3.3 | 0.00 | similar to unknown proteins |
| SA0413 |  | Conserved hypothetical protein | 3.3 | 0.00 | similar to unknown proteins |
| SA0861 |  | Conserved hypothetical protein | 3.3 | 0.00 | similar to unknown proteins |
| SA0170 |  | Conserved hypothetical protein | 3.4 | 0.00 | similar to unknown proteins |
| SA1057 |  | Conserved hypothetical protein | 3.4 | 0.00 | similar to unknown proteins |
| SA2262 |  | Conserved hypothetical protein | 3.4 | 0.00 | similar to unknown proteins |
| SA1020 |  | Conserved hypothetical protein | 3.4 | 0.00 | similar to unknown proteins |
| SA1431 |  | Conserved hypothetical protein | 3.4 | 0.00 | similar to unknown proteins |
| SA1613 |  | Conserved hypothetical protein | 3.6 | 0.00 | similar to unknown proteins |
| SA1451 |  | Conserved hypothetical protein | 3.6 | 0.00 | similar to unknown proteins |
| SA1544 |  | Hypothetical protein, similar to soluble hydrogenase 42 kD subunit | 3.7 | 0.00 | similar to unknown proteins |
| SA2195 |  | Conserved hypothetical protein | 3.8 | 0.00 | similar to unknown proteins |
| SA0306 |  | Conserved hypothetical protein | 3.8 | 0.00 | similar to unknown proteins |
| SA0860 |  | Conserved hypothetical protein | 3.9 | 0.00 | similar to unknown proteins |
| SA0481 |  | Conserved hypothetical protein | 4.2 | 0.00 | similar to unknown proteins |
| SA0801 |  | Conserved hypothetical protein | 4.2 | 0.00 | similar to unknown proteins |
| SA1020 |  | Conserved hypothetical protein | 4.3 | 0.00 | similar to unknown proteins |
| SA0612 |  | Conserved hypothetical protein | 4.6 | 0.00 | similar to unknown proteins |
| SA0412 |  | Conserved hypothetical protein | 4.7 | 0.00 | similar to unknown proteins |
| SA1990 |  | Conserved hypothetical protein | 4.7 | 0.00 | similar to unknown proteins |
| SA1543 |  | Conserved hypothetical protein | 5.0 | 0.00 | similar to unknown proteins |
| SA2161 |  | Hypothetical protein, similar to attachment to host cells and virulence | 5.1 | 0.00 | similar to unknown proteins |
| SA2138 |  | Conserved hypothetical protein | 5.1 | 0.00 | similar to unknown proteins |
| SA1686 |  | Conserved hypothetical protein | 5.3 | 0.00 | similar to unknown proteins |
| SA0380 |  | Conserved hypothetical protein [Pathogenicity island SaPIn2] | 5.9 | 0.00 | similar to unknown proteins |
| SA1702 |  | Conserved hypothetical protein | 6.2 | 0.00 | similar to unknown proteins |
| SA0230 |  | Conserved hypothetical protein | 6.4 | 0.00 | similar to unknown proteins |
| SA0703 |  | Conserved hypothetical protein | 6.7 | 0.00 | similar to unknown proteins |
| SA0381 |  | Conserved hypothetical protein [Pathogenicity island SaPIn2] | 7.3 | 0.00 | similar to unknown proteins |
| SA1235 |  | Conserved hypothetical protein | 7.5 | 0.00 | similar to unknown proteins |
| SA0800 |  | Conserved hypothetical protein | 8.0 | 0.00 | similar to unknown proteins |
| SA2325 |  | Conserved hypothetical protein | 11.6 | 0.00 | similar to unknown proteins |
| SA2329 | *cidA* | Hypothetical protein,similar to transcription regulator | -2.0 | 0.00 | Similar to unknown proteins |
| SA1417 | *comEB* | Late competence operon required for DNA binding and uptake comEB | 2.9 | 0.06 | [Transformation / competence](http://www.bio.nite.go.jp/dogan/GeneSearchResult?GENE_LIST_TYPE=1&type=504&GENOME_LIST=n315G1&CLASS_ID=31.10&WITH_GENE_MAP=1) |
| SA0857 |  | Hypothetical protein, similar to negative regulator of genetic competence MecA | 4.2 | 0.00 | [Transformation / competence](http://www.bio.nite.go.jp/dogan/GeneSearchResult?GENE_LIST_TYPE=1&type=504&GENOME_LIST=n315G1&CLASS_ID=31.10&WITH_GENE_MAP=1) |
| SA2408 | *cudT* | Choline transporter | -39.6 | 0.00 | [Transport / binding proteins and lipoproteins](http://www.bio.nite.go.jp/dogan/GeneSearchResult?GENE_LIST_TYPE=1&type=504&GENOME_LIST=n315G1&CLASS_ID=31.02&WITH_GENE_MAP=1) |
| SA2074 | *modA* | Probable molybdate-binding protein | -20.1 | 0.00 | [Transport / binding proteins and lipoproteins](http://www.bio.nite.go.jp/dogan/GeneSearchResult?GENE_LIST_TYPE=1&type=504&GENOME_LIST=n315G1&CLASS_ID=31.02&WITH_GENE_MAP=1) |
| SA0272 |  | Hypothetical protein, similar to transmembrane protein Tmp7 | -16.2 | 0.00 | [Transport / binding proteins and lipoproteins](http://www.bio.nite.go.jp/dogan/GeneSearchResult?GENE_LIST_TYPE=1&type=504&GENOME_LIST=n315G1&CLASS_ID=31.02&WITH_GENE_MAP=1) |
| SA0267 |  | Hypothetical protein | -14.9 | 0.00 | Transport / binding proteins and lipoproteins |
| SA2303 |  | Hypothetical protein, etracy to membrane spanning protein | -12.9 | 0.00 | [Transport / binding proteins and lipoproteins](http://www.bio.nite.go.jp/dogan/GeneSearchResult?GENE_LIST_TYPE=1&type=504&GENOME_LIST=n315G1&CLASS_ID=31.02&WITH_GENE_MAP=1) |
| SA2135 |  | Hypothetical protein, similar to sodium/glutamate symporter | -12.8 | 0.00 | [Transport / binding proteins and lipoproteins](http://www.bio.nite.go.jp/dogan/GeneSearchResult?GENE_LIST_TYPE=1&type=504&GENOME_LIST=n315G1&CLASS_ID=31.02&WITH_GENE_MAP=1) |
| SA0325 | *glpT* | Glycerol-3-phosphate transporter | -10.8 | 0.00 | Transport / binding proteins and lipoproteins |
| SA2302 |  | Hypothetical protein, similar to ABC transporter | -10.3 | 0.00 | [Transport / binding proteins and lipoproteins](http://www.bio.nite.go.jp/dogan/GeneSearchResult?GENE_LIST_TYPE=1&type=504&GENOME_LIST=n315G1&CLASS_ID=31.02&WITH_GENE_MAP=1) |
| SA0374 | *pbuX* | Xanthine permease | -9.1 | 0.00 | [Transport / binding proteins and lipoproteins](http://www.bio.nite.go.jp/dogan/GeneSearchResult?GENE_LIST_TYPE=1&type=504&GENOME_LIST=n315G1&CLASS_ID=31.02&WITH_GENE_MAP=1) |
| SA2303 |  | Hypothetical protein, etracy to membrane spanning protein | -7.4 | 0.00 | [Transport / binding proteins and lipoproteins](http://www.bio.nite.go.jp/dogan/GeneSearchResult?GENE_LIST_TYPE=1&type=504&GENOME_LIST=n315G1&CLASS_ID=31.02&WITH_GENE_MAP=1) |
| SA0168 |  | Hypothetical protein, similar to probable permease of ABC transporter | -7.3 | 0.00 | [Transport / binding proteins and lipoproteins](http://www.bio.nite.go.jp/dogan/GeneSearchResult?GENE_LIST_TYPE=1&type=504&GENOME_LIST=n315G1&CLASS_ID=31.02&WITH_GENE_MAP=1) |
| SA0266 |  | Conserved hypothetical protein | -7.3 | 0.00 | [Transport / binding proteins and lipoproteins](http://www.bio.nite.go.jp/dogan/GeneSearchResult?GENE_LIST_TYPE=1&type=504&GENOME_LIST=n315G1&CLASS_ID=31.02&WITH_GENE_MAP=1) |
| SA0325 | *glpT* | Glycerol-3-phosphate transporter | -7.2 | 0.00 | [Transport / binding proteins and lipoproteins](http://www.bio.nite.go.jp/dogan/GeneSearchResult?GENE_LIST_TYPE=1&type=504&GENOME_LIST=n315G1&CLASS_ID=31.02&WITH_GENE_MAP=1) |
| SA2073 | *modB* | Probable molybdenum transport permease | -7.1 | 0.00 | [Transport / binding proteins and lipoproteins](http://www.bio.nite.go.jp/dogan/GeneSearchResult?GENE_LIST_TYPE=1&type=504&GENOME_LIST=n315G1&CLASS_ID=31.02&WITH_GENE_MAP=1) |
| SA0138 |  | Hypothetical protein, similar to alkylphosphonate ABC tranporter | -6.8 | 0.00 | [Transport / binding proteins and lipoproteins](http://www.bio.nite.go.jp/dogan/GeneSearchResult?GENE_LIST_TYPE=1&type=504&GENOME_LIST=n315G1&CLASS_ID=31.02&WITH_GENE_MAP=1) |
| SA0796 | *dltD* | Poly(glycerophosphate chain) D-alanine transfer protein | -6.7 | 0.00 | [Transport / binding proteins and lipoproteins](http://www.bio.nite.go.jp/dogan/GeneSearchResult?GENE_LIST_TYPE=1&type=504&GENOME_LIST=n315G1&CLASS_ID=31.02&WITH_GENE_MAP=1) |
| SA2072 | *modC* | Molybdenum transport ATP-binding protein ModC | -6.4 | 0.00 | [Transport / binding proteins and lipoproteins](http://www.bio.nite.go.jp/dogan/GeneSearchResult?GENE_LIST_TYPE=1&type=504&GENOME_LIST=n315G1&CLASS_ID=31.02&WITH_GENE_MAP=1) |
| SA0950 | *potA* | Spermidine/putrescine ABC transporter, ATP-binding protein homolog | -6.4 | 0.00 | [Transport / binding proteins and lipoproteins](http://www.bio.nite.go.jp/dogan/GeneSearchResult?GENE_LIST_TYPE=1&type=504&GENOME_LIST=n315G1&CLASS_ID=31.02&WITH_GENE_MAP=1) |
| SA0368 |  | Hypothetical protein, similar to proton/sodium-glutamate symport protein | -6.0 | 0.00 | [Transport / binding proteins and lipoproteins](http://www.bio.nite.go.jp/dogan/GeneSearchResult?GENE_LIST_TYPE=1&type=504&GENOME_LIST=n315G1&CLASS_ID=31.02&WITH_GENE_MAP=1) |
| SA1183 | *opuD* | Glycine betaine transporter | -6.0 | 0.00 | Transport / binding proteins and lipoproteins |
| SA1183 | *opuD* | Glycine betaine transporter | -5.6 | 0.00 | [Transport / binding proteins and lipoproteins](http://www.bio.nite.go.jp/dogan/GeneSearchResult?GENE_LIST_TYPE=1&type=504&GENOME_LIST=n315G1&CLASS_ID=31.02&WITH_GENE_MAP=1) |
| SA0479 | *nupC* | Pyrimidine nucleoside transport protein | -5.2 | 0.00 | [Transport / binding proteins and lipoproteins](http://www.bio.nite.go.jp/dogan/GeneSearchResult?GENE_LIST_TYPE=1&type=504&GENOME_LIST=n315G1&CLASS_ID=31.02&WITH_GENE_MAP=1) |
| SA0420 |  | Hypothetical protein, etracy to ABC transporter ATP-binding protein | -5.0 | 0.00 | [Transport / binding proteins and lipoproteins](http://www.bio.nite.go.jp/dogan/GeneSearchResult?GENE_LIST_TYPE=1&type=504&GENOME_LIST=n315G1&CLASS_ID=31.02&WITH_GENE_MAP=1) |
| SA0136 |  | Hypothetical protein, similar to phosphonates transport permease | -5.0 | 0.00 | [Transport / binding proteins and lipoproteins](http://www.bio.nite.go.jp/dogan/GeneSearchResult?GENE_LIST_TYPE=1&type=504&GENOME_LIST=n315G1&CLASS_ID=31.02&WITH_GENE_MAP=1) |
| SA2132 |  | Hypothetical protein, etracy to ABC transporter (ATP-binding protein) | -4.8 | 0.00 | [Transport / binding proteins and lipoproteins](http://www.bio.nite.go.jp/dogan/GeneSearchResult?GENE_LIST_TYPE=1&type=504&GENOME_LIST=n315G1&CLASS_ID=31.02&WITH_GENE_MAP=1) |
| SA2254 |  | Oligopeptide transporter putative membrane permease domain | -4.8 | 0.00 | [Transport / binding proteins and lipoproteins](http://www.bio.nite.go.jp/dogan/GeneSearchResult?GENE_LIST_TYPE=1&type=504&GENOME_LIST=n315G1&CLASS_ID=31.02&WITH_GENE_MAP=1) |
| SA0111 | *sirA* | Lipoprotein | -4.7 | 0.00 | [Transport / binding proteins and lipoproteins](http://www.bio.nite.go.jp/dogan/GeneSearchResult?GENE_LIST_TYPE=1&type=504&GENOME_LIST=n315G1&CLASS_ID=31.02&WITH_GENE_MAP=1) |
| SA2253 |  | Oligopeptide transporter putative membrane permease domain | -4.7 | 0.00 | [Transport / binding proteins and lipoproteins](http://www.bio.nite.go.jp/dogan/GeneSearchResult?GENE_LIST_TYPE=1&type=504&GENOME_LIST=n315G1&CLASS_ID=31.02&WITH_GENE_MAP=1) |
| SA0137 |  | Hypothetical protein, similar to transport system protein | -4.7 | 0.00 | [Transport / binding proteins and lipoproteins](http://www.bio.nite.go.jp/dogan/GeneSearchResult?GENE_LIST_TYPE=1&type=504&GENOME_LIST=n315G1&CLASS_ID=31.02&WITH_GENE_MAP=1) |
| SA0166 |  | Hypothetical protein, similar to nitrate transporter | -4.6 | 0.00 | [Transport / binding proteins and lipoproteins](http://www.bio.nite.go.jp/dogan/GeneSearchResult?GENE_LIST_TYPE=1&type=504&GENOME_LIST=n315G1&CLASS_ID=31.02&WITH_GENE_MAP=1) |
| SA1224 |  | ABC transporter (ATP-binding protein) homolog | -4.5 | 0.00 | [Transport / binding proteins and lipoproteins](http://www.bio.nite.go.jp/dogan/GeneSearchResult?GENE_LIST_TYPE=1&type=504&GENOME_LIST=n315G1&CLASS_ID=31.02&WITH_GENE_MAP=1) |
| SA1239 | *braB* | Branched-chain amino acid carrier protein | -4.4 | 0.00 | [Transport / binding proteins and lipoproteins](http://www.bio.nite.go.jp/dogan/GeneSearchResult?GENE_LIST_TYPE=1&type=504&GENOME_LIST=n315G1&CLASS_ID=31.02&WITH_GENE_MAP=1) |
| SA0206 | *msmX* | Multiple sugar-binding transport ATP-binding protein | -4.4 | 0.00 | [Transport / binding proteins and lipoproteins](http://www.bio.nite.go.jp/dogan/GeneSearchResult?GENE_LIST_TYPE=1&type=504&GENOME_LIST=n315G1&CLASS_ID=31.02&WITH_GENE_MAP=1) |
| SA0167 |  | Hypothetical protein, similar to membrane lipoprotein SrpL | -4.1 | 0.00 | [Transport / binding proteins and lipoproteins](http://www.bio.nite.go.jp/dogan/GeneSearchResult?GENE_LIST_TYPE=1&type=504&GENOME_LIST=n315G1&CLASS_ID=31.02&WITH_GENE_MAP=1) |
| SA0421 |  | Hypothetical protein, etracy to ABC transporter permease protein | -4.1 | 0.00 | [Transport / binding proteins and lipoproteins](http://www.bio.nite.go.jp/dogan/GeneSearchResult?GENE_LIST_TYPE=1&type=504&GENOME_LIST=n315G1&CLASS_ID=31.02&WITH_GENE_MAP=1) |
| SA0422 |  | Hypothetical protein, similar to lactococcal lipoprotein | -4.0 | 0.00 | [Transport / binding proteins and lipoproteins](http://www.bio.nite.go.jp/dogan/GeneSearchResult?GENE_LIST_TYPE=1&type=504&GENOME_LIST=n315G1&CLASS_ID=31.02&WITH_GENE_MAP=1) |
| SA0135 |  | Hypothetical protein, similar to phosphonates transport permease | -3.9 | 0.00 | [Transport / binding proteins and lipoproteins](http://www.bio.nite.go.jp/dogan/GeneSearchResult?GENE_LIST_TYPE=1&type=504&GENOME_LIST=n315G1&CLASS_ID=31.02&WITH_GENE_MAP=1) |
| SA0566 |  | Hypothetical protein, similar to iron-binding protein | -3.9 | 0.00 | [Transport / binding proteins and lipoproteins](http://www.bio.nite.go.jp/dogan/GeneSearchResult?GENE_LIST_TYPE=1&type=504&GENOME_LIST=n315G1&CLASS_ID=31.02&WITH_GENE_MAP=1) |
| SA2252 |  | Oligopeptide transporter putative ATPase domain | -3.9 | 0.00 | [Transport / binding proteins and lipoproteins](http://www.bio.nite.go.jp/dogan/GeneSearchResult?GENE_LIST_TYPE=1&type=504&GENOME_LIST=n315G1&CLASS_ID=31.02&WITH_GENE_MAP=1) |
| SA0794 | *dltB* | DltB membrane protein | -3.8 | 0.00 | [Transport / binding proteins and lipoproteins](http://www.bio.nite.go.jp/dogan/GeneSearchResult?GENE_LIST_TYPE=1&type=504&GENOME_LIST=n315G1&CLASS_ID=31.02&WITH_GENE_MAP=1) |
| SA2411 |  | Hypothetical protein, similar to magnesium citrate secondary transporter | -3.8 | 0.00 | [Transport / binding proteins and lipoproteins](http://www.bio.nite.go.jp/dogan/GeneSearchResult?GENE_LIST_TYPE=1&type=504&GENOME_LIST=n315G1&CLASS_ID=31.02&WITH_GENE_MAP=1) |
| SA2194 |  | Hypothetical protein, similar to Zn-binding lipoprotein adcA | -3.6 | 0.00 | [Transport / binding proteins and lipoproteins](http://www.bio.nite.go.jp/dogan/GeneSearchResult?GENE_LIST_TYPE=1&type=504&GENOME_LIST=n315G1&CLASS_ID=31.02&WITH_GENE_MAP=1) |
| SA1732 |  | Hypothetical protein, similar to sodium-dependent transporter | -3.5 | 0.00 | [Transport / binding proteins and lipoproteins](http://www.bio.nite.go.jp/dogan/GeneSearchResult?GENE_LIST_TYPE=1&type=504&GENOME_LIST=n315G1&CLASS_ID=31.02&WITH_GENE_MAP=1) |
| SA0106 | *lctP* | L-lactate permease homologue | -3.3 | 0.00 | [Transport / binding proteins and lipoproteins](http://www.bio.nite.go.jp/dogan/GeneSearchResult?GENE_LIST_TYPE=1&type=504&GENOME_LIST=n315G1&CLASS_ID=31.02&WITH_GENE_MAP=1) |
| SA0100 |  | Conserved hypothetical protein | -3.3 | 0.00 | [Transport / binding proteins and lipoproteins](http://www.bio.nite.go.jp/dogan/GeneSearchResult?GENE_LIST_TYPE=1&type=504&GENOME_LIST=n315G1&CLASS_ID=31.02&WITH_GENE_MAP=1) |
| SA0180 |  | Hypothetical protein, etracy to branched-chain amino acid transport system carrier protein | -3.1 | 0.00 | [Transport / binding proteins and lipoproteins](http://www.bio.nite.go.jp/dogan/GeneSearchResult?GENE_LIST_TYPE=1&type=504&GENOME_LIST=n315G1&CLASS_ID=31.02&WITH_GENE_MAP=1) |
| SA0980 | *isdE* | Hypothetical protein, similar to ferrichrome ABC transporter | -3.1 | 0.00 | [Transport / binding proteins and lipoproteins](http://www.bio.nite.go.jp/dogan/GeneSearchResult?GENE_LIST_TYPE=1&type=504&GENOME_LIST=n315G1&CLASS_ID=31.02&WITH_GENE_MAP=1) |
| SA0200 |  | Hypothetical protein, similar to dipeptide transporter protein dppC | -3.1 | 0.00 | [Transport / binding proteins and lipoproteins](http://www.bio.nite.go.jp/dogan/GeneSearchResult?GENE_LIST_TYPE=1&type=504&GENOME_LIST=n315G1&CLASS_ID=31.02&WITH_GENE_MAP=1) |
| SA1190 | *alsT* | Amino acid carrier protein (sodium/alanine symporter) | -2.9 | 0.00 | [Transport / binding proteins and lipoproteins](http://www.bio.nite.go.jp/dogan/GeneSearchResult?GENE_LIST_TYPE=1&type=504&GENOME_LIST=n315G1&CLASS_ID=31.02&WITH_GENE_MAP=1) |
| SA0303 |  | Hypothetical protein, similar to sodium-coupled permease | -2.9 | 0.00 | [Transport / binding proteins and lipoproteins](http://www.bio.nite.go.jp/dogan/GeneSearchResult?GENE_LIST_TYPE=1&type=504&GENOME_LIST=n315G1&CLASS_ID=31.02&WITH_GENE_MAP=1) |
| SA1547 | *ptaA* | PTS system, N-acetylglucosamine-specific IIABC component | -2.9 | 0.00 | [Transport / binding proteins and lipoproteins](http://www.bio.nite.go.jp/dogan/GeneSearchResult?GENE_LIST_TYPE=1&type=504&GENOME_LIST=n315G1&CLASS_ID=31.02&WITH_GENE_MAP=1) |
| SA2131 |  | Conserved hypothetical protein | -2.8 | 0.00 | [Transport / binding proteins and lipoproteins](http://www.bio.nite.go.jp/dogan/GeneSearchResult?GENE_LIST_TYPE=1&type=504&GENOME_LIST=n315G1&CLASS_ID=31.02&WITH_GENE_MAP=1) |
| SA2251 |  | Oligopeptide transporter putative ATPase domain | -2.8 | 0.00 | [Transport / binding proteins and lipoproteins](http://www.bio.nite.go.jp/dogan/GeneSearchResult?GENE_LIST_TYPE=1&type=504&GENOME_LIST=n315G1&CLASS_ID=31.02&WITH_GENE_MAP=1) |
| SA2061 |  | Hypothetical protein | -2.7 | 0.00 | [Transport / binding proteins and lipoproteins](http://www.bio.nite.go.jp/dogan/GeneSearchResult?GENE_LIST_TYPE=1&type=504&GENOME_LIST=n315G1&CLASS_ID=31.02&WITH_GENE_MAP=1) |
| SA1718 | *putP* | High affinity etracy permease | -2.7 | 0.00 | [Transport / binding proteins and lipoproteins](http://www.bio.nite.go.jp/dogan/GeneSearchResult?GENE_LIST_TYPE=1&type=504&GENOME_LIST=n315G1&CLASS_ID=31.02&WITH_GENE_MAP=1) |
| SA0010 |  | Hypothetical protein, similar to amino acid permease | -2.7 | 0.07 | Transport / binding proteins and lipoproteins |
| SA0981 | *isdF* | Hypothetical protein, similar to ferrichrome ABC transporter | -2.7 | 0.07 | [Transport / binding proteins and lipoproteins](http://www.bio.nite.go.jp/dogan/GeneSearchResult?GENE_LIST_TYPE=1&type=504&GENOME_LIST=n315G1&CLASS_ID=31.02&WITH_GENE_MAP=1) |
| SA0207 |  | Hypothetical protein, similar to maltose/maltodextrin-binding protein | -2.7 | 0.00 | [Transport / binding proteins and lipoproteins](http://www.bio.nite.go.jp/dogan/GeneSearchResult?GENE_LIST_TYPE=1&type=504&GENOME_LIST=n315G1&CLASS_ID=31.02&WITH_GENE_MAP=1) |
| SA2156 |  | L-lactate permease lctP homolog | -2.6 | 0.07 | [Transport / binding proteins and lipoproteins](http://www.bio.nite.go.jp/dogan/GeneSearchResult?GENE_LIST_TYPE=1&type=504&GENOME_LIST=n315G1&CLASS_ID=31.02&WITH_GENE_MAP=1) |
| SA1140 | *glpF* | Glycerol uptake facilitator | -2.5 | 0.07 | [Transport / binding proteins and lipoproteins](http://www.bio.nite.go.jp/dogan/GeneSearchResult?GENE_LIST_TYPE=1&type=504&GENOME_LIST=n315G1&CLASS_ID=31.02&WITH_GENE_MAP=1) |
| SA1580 |  | Multidrug resistance protein homolog | -2.4 | 0.07 | Transport / binding proteins and lipoproteins |
| SA1341 |  | Hypothetical protein, similar to export protein SpcT protein | -2.4 | 0.11 | Transport / binding proteins and lipoproteins |
| SA2050 |  | Conserved hypothetical protein | -2.4 | 0.07 | Transport / binding proteins and lipoproteins |
| SA2322 |  | Conserved hypothetical protein | -2.4 | 0.07 | Transport / binding proteins and lipoproteins |
| SA0953 | *potD* | Spermidine/putrescine-binding protein precursor homolog | -2.4 | 0.07 | [Transport / binding proteins and lipoproteins](http://www.bio.nite.go.jp/dogan/GeneSearchResult?GENE_LIST_TYPE=1&type=504&GENOME_LIST=n315G1&CLASS_ID=31.02&WITH_GENE_MAP=1) |
| SA2216 |  | Hypothetical protein, etracy to ABC transporter, ATP-binding protein | -2.4 | 0.07 | [Transport / binding proteins and lipoproteins](http://www.bio.nite.go.jp/dogan/GeneSearchResult?GENE_LIST_TYPE=1&type=504&GENOME_LIST=n315G1&CLASS_ID=31.02&WITH_GENE_MAP=1) |
| SA2222 |  | Hypothetical protein, similar to bicyclomycin resistance protein TcaB | -2.3 | 0.11 | Transport / binding proteins and lipoproteins |
| SA2241 |  | Hypothetical protein, similar to chloramphenicol resistance protein | -2.2 | 0.13 | Transport / binding proteins and lipoproteins |
| SA0201 | *rlp* | RGD-containing lipoprotein | -2.2 | 0.13 | [Transport / binding proteins and lipoproteins](http://www.bio.nite.go.jp/dogan/GeneSearchResult?GENE_LIST_TYPE=1&type=504&GENOME_LIST=n315G1&CLASS_ID=31.02&WITH_GENE_MAP=1) |
| SA0295 |  | Hypothetical protein, similar to outer membrane protein precursor | -2.2 | 0.11 | [Transport / binding proteins and lipoproteins](http://www.bio.nite.go.jp/dogan/GeneSearchResult?GENE_LIST_TYPE=1&type=504&GENOME_LIST=n315G1&CLASS_ID=31.02&WITH_GENE_MAP=1) |
| SA0880 |  | Hypothetical protein, similar to Na^+^-transporting ATP synthase | -2.1 | 0.19 | Transport / binding proteins and lipoproteins |
| SA0684 |  | Conserved hypothetical protein | -2.1 | 0.19 | Transport / binding proteins and lipoproteins |
| SA0804 |  | Conserved hypothetical protein | -2.1 | 0.2 | Transport / binding proteins and lipoproteins |
| SA2475 |  | Conserved hypothetical protein | -2.1 | 0.25 | [Transport / binding proteins and lipoproteins](http://www.bio.nite.go.jp/dogan/GeneSearchResult?GENE_LIST_TYPE=1&type=504&GENOME_LIST=n315G1&CLASS_ID=31.02&WITH_GENE_MAP=1) |
| SA1042 | *pyrP* | Uracil permease | -2.0 | 0.61 | [Transport / binding proteins and lipoproteins](http://www.bio.nite.go.jp/dogan/GeneSearchResult?GENE_LIST_TYPE=1&type=504&GENOME_LIST=n315G1&CLASS_ID=31.02&WITH_GENE_MAP=1) |
| SA0600 |  | Hypothetical protein, similar to pyrimidine nucleoside transporter | -2.0 | 0.25 | [Transport / binding proteins and lipoproteins](http://www.bio.nite.go.jp/dogan/GeneSearchResult?GENE_LIST_TYPE=1&type=504&GENOME_LIST=n315G1&CLASS_ID=31.02&WITH_GENE_MAP=1) |
| SA0682 |  | Hypothetical protein, similar to di-tripepride ABC transporter | -2.0 | 0.25 | [Transport / binding proteins and lipoproteins](http://www.bio.nite.go.jp/dogan/GeneSearchResult?GENE_LIST_TYPE=1&type=504&GENOME_LIST=n315G1&CLASS_ID=31.02&WITH_GENE_MAP=1) |
| SA0845 | *oppB* | Oligopeptide transport system permease protein | -2.0 | 0.20 | [Transport / binding proteins and lipoproteins](http://www.bio.nite.go.jp/dogan/GeneSearchResult?GENE_LIST_TYPE=1&type=504&GENOME_LIST=n315G1&CLASS_ID=31.02&WITH_GENE_MAP=1) |
| SA2217 |  | Hypothetical protein, etracy to lipoprotein inner membrane ABC-transporter | -2.0 | 0.25 | [Transport / binding proteins and lipoproteins](http://www.bio.nite.go.jp/dogan/GeneSearchResult?GENE_LIST_TYPE=1&type=504&GENOME_LIST=n315G1&CLASS_ID=31.02&WITH_GENE_MAP=1) |
| SA0217 |  | Hypothetical protein, similar to periplasmic-iron-binding protein BitC | -2.0 | 0.20 | [Transport / binding proteins and lipoproteins](http://www.bio.nite.go.jp/dogan/GeneSearchResult?GENE_LIST_TYPE=1&type=504&GENOME_LIST=n315G1&CLASS_ID=31.02&WITH_GENE_MAP=1) |
| SA0099 |  | Hypothetical protein, similar to transmembrane efflux pump protein | 2.0 | 0.38 | [Transport / binding proteins and lipoproteins](http://www.bio.nite.go.jp/dogan/GeneSearchResult?GENE_LIST_TYPE=1&type=504&GENOME_LIST=n315G1&CLASS_ID=31.02&WITH_GENE_MAP=1) |
| SA2203 |  | Hypothetical protein, etracy to multidrug resistance protein | 2.1 | 0.40 | [Transport / binding proteins and lipoproteins](http://www.bio.nite.go.jp/dogan/GeneSearchResult?GENE_LIST_TYPE=1&type=504&GENOME_LIST=n315G1&CLASS_ID=31.02&WITH_GENE_MAP=1) |
| SA2054 |  | Conserved hypothetical protein | 2.1 | 0.61 | Transport / binding proteins and lipoproteins |
| SA2236 | *opuCB* | Probable glycine betaine/carnitine/choline ABC transporter (membrane p) opuCB | 2.1 | 0.38 | [Transport / binding proteins and lipoproteins](http://www.bio.nite.go.jp/dogan/GeneSearchResult?GENE_LIST_TYPE=1&type=504&GENOME_LIST=n315G1&CLASS_ID=31.02&WITH_GENE_MAP=1) |
| SA2237 | *opuCA* | Glycine betaine/carnitine/choline ABC transporter (ATP-bindin) opuCA | 2.2 | 0.20 | [Transport / binding proteins and lipoproteins](http://www.bio.nite.go.jp/dogan/GeneSearchResult?GENE_LIST_TYPE=1&type=504&GENOME_LIST=n315G1&CLASS_ID=31.02&WITH_GENE_MAP=1) |
| SA2487 |  | Hypothetical protein, similar to rarD protein | 2.5 | 0.13 | Transport / binding proteins and lipoproteins |
| SA2396 |  | Hypothetical protein, similar to amino acid transporter | 2.6 | 0.11 | [Transport / binding proteins and lipoproteins](http://www.bio.nite.go.jp/dogan/GeneSearchResult?GENE_LIST_TYPE=1&type=504&GENOME_LIST=n315G1&CLASS_ID=31.02&WITH_GENE_MAP=1) |
| SA0891 |  | Hypothetical protein, similar to ferrichrome ABC transporter | 2.6 | 0.11 | [Transport / binding proteins and lipoproteins](http://www.bio.nite.go.jp/dogan/GeneSearchResult?GENE_LIST_TYPE=1&type=504&GENOME_LIST=n315G1&CLASS_ID=31.02&WITH_GENE_MAP=1) |
| SA0172 |  | Hypothetical protein, similar to integral membrane protein LmrP | 2.7 | 0.06 | Transport / binding proteins and lipoproteins |
| SA0956 |  | Hypothetical protein, similar to Mn^2+^-transport protein | 2.8 | 0.00 | [Transport / binding proteins and lipoproteins](http://www.bio.nite.go.jp/dogan/GeneSearchResult?GENE_LIST_TYPE=1&type=504&GENOME_LIST=n315G1&CLASS_ID=31.02&WITH_GENE_MAP=1) |
| SA2344 | *copA* | Copper-transporting ATPase copA | 2.8 | 0.00 | Transport / binding proteins and lipoproteins |
| SA0294 |  | Hypothetical protein, similar to branched-chain amino acid uptake carrier | 2.9 | 0.00 | [Transport / binding proteins and lipoproteins](http://www.bio.nite.go.jp/dogan/GeneSearchResult?GENE_LIST_TYPE=1&type=504&GENOME_LIST=n315G1&CLASS_ID=31.02&WITH_GENE_MAP=1) |
| SA2081 |  | Hypothetical protein, similar to urea transporter | 3.1 | 0.00 | [Transport / binding proteins and lipoproteins](http://www.bio.nite.go.jp/dogan/GeneSearchResult?GENE_LIST_TYPE=1&type=504&GENOME_LIST=n315G1&CLASS_ID=31.02&WITH_GENE_MAP=1) |
| SA1948 | *czrB* | Cation-efflux system membrane protein homolog | 3.1 | 0.00 | [Transport / binding proteins and lipoproteins](http://www.bio.nite.go.jp/dogan/GeneSearchResult?GENE_LIST_TYPE=1&type=504&GENOME_LIST=n315G1&CLASS_ID=31.02&WITH_GENE_MAP=1) |
| SA0593 | *tagH* | Teichoic acid translocation ATP-binding protein | 3.2 | 0.00 | Transport / binding proteins and lipoproteins |
| SA2149 |  | Hypothetical protein, etracy to ABC transporter, ATP-binding protein | 3.3 | 0.00 | [Transport / binding proteins and lipoproteins](http://www.bio.nite.go.jp/dogan/GeneSearchResult?GENE_LIST_TYPE=1&type=504&GENOME_LIST=n315G1&CLASS_ID=31.02&WITH_GENE_MAP=1) |
| SA2150 |  | Conserved hypothetical protein | 3.6 | 0.00 | Transport / binding proteins and lipoproteins |
| SA0585 |  | Conserved hypothetical protein | 3.8 | 0.00 | Transport / binding proteins and lipoproteins |
| SA0132 |  | Hypothetical protein, similar to etracycline resistance protein | 4.2 | 0.00 | [Transport / binding proteins and lipoproteins](http://www.bio.nite.go.jp/dogan/GeneSearchResult?GENE_LIST_TYPE=1&type=504&GENOME_LIST=n315G1&CLASS_ID=31.02&WITH_GENE_MAP=1) |
| SA2293 | *gntP* | Gluconate permease | 4.9 | 0.00 | [Transport / binding proteins and lipoproteins](http://www.bio.nite.go.jp/dogan/GeneSearchResult?GENE_LIST_TYPE=1&type=504&GENOME_LIST=n315G1&CLASS_ID=31.02&WITH_GENE_MAP=1) |
| SA0229 |  | Hypothetical protein, similar to nickel ABC transporter nickel-binding protein | 6.8 | 0.00 | [Transport / binding proteins and lipoproteins](http://www.bio.nite.go.jp/dogan/GeneSearchResult?GENE_LIST_TYPE=1&type=504&GENOME_LIST=n315G1&CLASS_ID=31.02&WITH_GENE_MAP=1) |
| SA2339 |  | Hypothetical protein, similar to antibiotic transport-associated protein | 7.1 | 0.00 | [Transport / binding proteins and lipoproteins](http://www.bio.nite.go.jp/dogan/GeneSearchResult?GENE_LIST_TYPE=1&type=504&GENOME_LIST=n315G1&CLASS_ID=31.02&WITH_GENE_MAP=1) |
| SA0952 | *potC* | Spermidine/putrescine ABC transporter homolog | -7.0 | 0.00 | [Transport / binding proteins and lipoproteins](http://www.bio.nite.go.jp/dogan/GeneSearchResult?GENE_LIST_TYPE=1&type=504&GENOME_LIST=n315G1&CLASS_ID=31.02&WITH_GENE_MAP=1) |
| SA0951 | *potB* | Spermidine/putrescine ABC transporter homolog | -6.3 | 0.00 | [Transport / binding proteins and lipoproteins](http://www.bio.nite.go.jp/dogan/GeneSearchResult?GENE_LIST_TYPE=1&type=504&GENOME_LIST=n315G1&CLASS_ID=31.02&WITH_GENE_MAP=1) |
| SA2172 | *gltT* | Proton/sodium-glutamate symport protein | -5.4 | 0.00 | [Transport / binding proteins and lipoproteins](http://www.bio.nite.go.jp/dogan/GeneSearchResult?GENE_LIST_TYPE=1&type=504&GENOME_LIST=n315G1&CLASS_ID=31.02&WITH_GENE_MAP=1) |
| SA0198 | *oppF* | Oligopeptide transport ATP-binding protein | -4.6 | 0.00 | [Transport / binding proteins and lipoproteins](http://www.bio.nite.go.jp/dogan/GeneSearchResult?GENE_LIST_TYPE=1&type=504&GENOME_LIST=n315G1&CLASS_ID=31.02&WITH_GENE_MAP=1) |
| SA0183 | *glcA* | PTS enzyme II (EC 2.7.1.69), glucose-specific, factor IIA homologue | -4.4 | 0.00 | [Transport / binding proteins and lipoproteins](http://www.bio.nite.go.jp/dogan/GeneSearchResult?GENE_LIST_TYPE=1&type=504&GENOME_LIST=n315G1&CLASS_ID=31.02&WITH_GENE_MAP=1) |
| SA0110 | *sirB* | Lipoprotein | -3.7 | 0.00 | [Transport / binding proteins and lipoproteins](http://www.bio.nite.go.jp/dogan/GeneSearchResult?GENE_LIST_TYPE=1&type=504&GENOME_LIST=n315G1&CLASS_ID=31.02&WITH_GENE_MAP=1) |
| SA1182 | *mscL* | Large-conductance mechanosensitive channel | -2.7 | 0.07 | [Transport / binding proteins and lipoproteins](http://www.bio.nite.go.jp/dogan/GeneSearchResult?GENE_LIST_TYPE=1&type=504&GENOME_LIST=n315G1&CLASS_ID=31.02&WITH_GENE_MAP=1) |
| SA2176 | *narK* | Nitrite extrusion protein | -2.1 | 0.19 | [Transport / binding proteins and lipoproteins](http://www.bio.nite.go.jp/dogan/GeneSearchResult?GENE_LIST_TYPE=1&type=504&GENOME_LIST=n315G1&CLASS_ID=31.02&WITH_GENE_MAP=1) |
| SA1255 |  | PTS system, glucose-specific enzyme II, A component | 2.3 | 0.19 | [Transport / binding proteins and lipoproteins](http://www.bio.nite.go.jp/dogan/GeneSearchResult?GENE_LIST_TYPE=1&type=504&GENOME_LIST=n315G1&CLASS_ID=31.02&WITH_GENE_MAP=1) |
| SA2148 |  | Hypothetical protein, similar to membrane protein | 2.4 | 0.19 | [Transport / binding proteins and lipoproteins](http://www.bio.nite.go.jp/dogan/GeneSearchResult?GENE_LIST_TYPE=1&type=504&GENOME_LIST=n315G1&CLASS_ID=31.02&WITH_GENE_MAP=1) |

*^a^* ORF no are derived from strain N315.

*^b^* Genes with expression changes upon treatment with 8 µg/ml sodium houttuyfonate.

*^c^* Fold change refers to expression increases or decreases for upregulated or downregulated genes, respectively.

*^d^* Microarray data were analyzed using SAM, significantly differentially regulated genes after filtering at 5% FDR and fold change greater than 2.

**Table S2.** Genes involved in known as related autolysis regulators affected by SH.

| **N315 SA**  **no.** | **Gene** | **Description** | **Fold change***^a,b^* | ***q* value (%)** |
| --- | --- | --- | --- | --- |
| SA0702 | *llm/tagO* | Lipophilic protein affecting bacterial lysis rate and methicillin resistance level | 1.0 | 38.61 |
| SA0909 | *fmtA* | Autolysis and methicillin resistant-related protein | Absent |  |
| SA2062 | *sarV* | Staphylococcal accessory regulator A homolog | 3.2^**^ | 0.00 |
| SA1964 | *fmtB* | FmtB protein | 1.6 | 4.22 |
| SA1902 | *murA* | UDP-N-acetylglucosamine 1-carboxyvinyl transferase 1 | -1.1 | 25.38 |
| SA0693 | *murB* | UDP-N-acetylenolpyruvoylglucosamine reductase | -1.0 | 34.30 |
| SA1562 | *murC* | DNA translocase stage III sporulation protein homolog | -1.3 | 9.33 |
| SA1026 | *murD* | UDP-N-acetylmuramoylalanine–D-glutamate ligase | -1.3 | 11.69 |
| SA0876 | *murE* | UDP-N-acetylmuramoylalanyl-D-glutamate–2,6-diaminopimelate ligase | -1.5^*^ | 2.69 |
| SA1886 | *murF* | UDP-N-acetylmuramoylalanyl-D-glutamyl-2,6-diaminopimelate-D-alanyl-D-alanyl ligase | -1.1 | 25.38 |
| SA1926 | *murZ* | UDP-N-acetylglucosamine 1-carboxylvinyl transferase 2 | 1.4 | 9.33 |
| SA1251 | *murG* | Undecaprenyl-PP-MurNAc-pentapeptide-UDPGlcNAc GlcNAc transferase | -1.2 | 16.15 |
| SA0997 | *murI* | Glutamate racemase | 1.5 | 3.21 |
| SA1025 | *mraY* | Phospho-N-muramic acid-pentapeptide translocase | -2.0^**^ | 0.20 |
| SA1898 | *sceD* | Hypothetical protein, simialr to SceD precursor | -3.0^**^ | 0.00 |
| SA2356 | *isaA* | Immunodominant antigen A | -11.5^**^ | 0.00 |
| SA2093 | *ssaA* | Secretory antigen precursor SsaA homolog | -7.8^**^ | 0.00 |
| SA0620 |  | Secretory antigen SsaA homologue | -5.9^**^ | 0.00 |
| SA0710 |  | Hypothetical protein | -1.1 | 37.74 |
| SA2097 |  | Hypothetical protein, similar to secretory antigen precursor SsaA | -13.3^**^ | 0.00 |
| SA2353 |  | Hypothetical protein, similar to secretory antigen precursor SsaA | -5.8^**^ | 0.00 |
| SA1700 | *vraR* | Two-component response regulator | 4.2^**^ | 0.00 |
| SA1701 | *vraS* | Two-component sensor histidine kinase | 3.6^**^ | 0.00 |
| SA1869 | *sigB* | Sigma factor B | -2.3^**^ | 0.07 |
| SA1872 | *rsbU* | Sigma B regulation protein RsbU | -3.0^**^ | 0.00 |
| SA1871 | *rsbV* | Anti-sigma B factor antagonist | -3.0^**^ | 0.00 |
| SA1870 | *rsbW* | Serine-protein kinase RsbW | -2.5^**^ | 0.07 |
| SA1984 | *asp23* | Alkaline shock protein 23, Asp23 | 1.3 | 20.98 |
| SA0108 | *sarS* | Staphylococcal accessory regulator A homologue | -2.0^**^ | 0.19 |
| SA0018 | *yycG* | Two-component sensor histidine kinase | 1.1 | 35.03 |
| SA0017 | *yycF* | Response regulator | 1.2 | 25.38 |
| SA1323 | *srrA* | Staphylococcal respiratory response protein SrrA | -1.2 | 23.22 |
| SA1322 | *srrB* | Staphylococcal respiratory response protein SrrB | -1.4 | 4.75 |
| SA0661 | *saeR* | Response regulator | 1.1 | 37.04 |
| SA0660 | *saeS* | Histidine protein kinase | 1.1 | 38.61 |
| SA1583 | *rot* | Repressor of toxins Rot | 1.3 | 23.22 |
| SA2287 | *sarU* | Staphylococcal accessory regulator A homolog | Absent |  |
| SA2286 | *sarT* | Staphylococcal accessory regulator A homolog | Absent |  |
| SA2089 | *sarR* | Staphylococcal accessory regulator R | Absent |  |
| SA1843 | *agrC* | Accessory gene regulator C | Absent |  |
| SA0901 | *sspA* | Cysteine protease/V8 protease | -5.4^**^ | 0.00 |
| SA0900 | *sspB* | Cysteine protease precursor | -2.6^**^ | 0.07 |
| SA0899 | *sspC* | Cysteine protease | -2.0^**^ | 0.25 |
| SA1725 | *scpA* | Staphopain, cysteine proteinase | -8.8^**^ | 0.00 |
| SA1726 | *scpB* | Hypothetical protein | -6.8^**^ | 0.00 |
| SA0879 | *htrA* | Serine protease HtrA | -3.3^**^ | 0.00 |
| SA0723 | *clpP* | Proteases ClpP | 3.5^**^ | 0.00 |

*^a^* － indicates reduction and ＋ indicates increase.

*^b^* Microarray data were analyzed using SAM, ^**^ significantly differentially regulated genes after filtering at 5% FDR and fold change greater than 2; ^*^ Significantly differentially regulated genes after filtering at 5% FDR and fold change greater than 1.5.
